# Radical Anions of Porphyrin Molecular Wires: Delocalization and Dynamics

**DOI:** 10.1021/jacs.4c14161

**Published:** 2024-12-30

**Authors:** Janko Hergenhahn, Jake M. Holmes, Jie-Ren Deng, Henrik Gotfredsen, Robert M. J. Jacobs, Sebastian M. Kopp, Christiane R. Timmel, Harry L. Anderson

**Affiliations:** † Centre for Advanced Electron Spin Resonance, Department of Chemistry, University of Oxford, Oxford OX1 3QR, U.K.; ‡ Chemistry Research Laboratory, Department of Chemistry, University of Oxford, Oxford OX1 3TA, U.K.

## Abstract

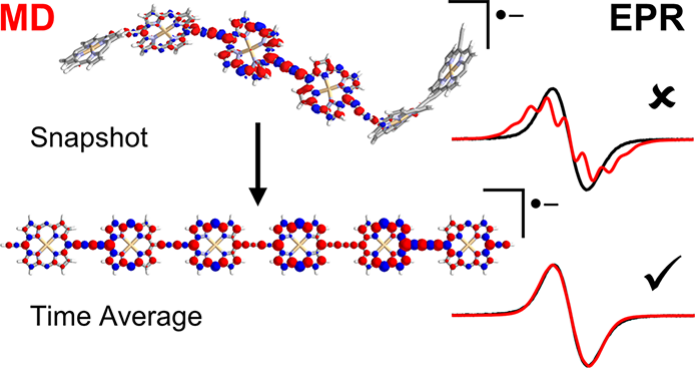

The delocalization length of charge carriers in organic semiconductors influences their mobility and is an important factor in the design of functional materials. Here, we have studied the radical anions of a series of linear and cyclic butadiyne-linked porphyrin oligomers using CW-EPR, ^1^H Mims ENDOR and NIR/MIR spectroelectrochemistry together with DFT calculations and multiscale molecular modeling. Low-temperature hyperfine EPR spectroscopy and optical data show that polarons are delocalized nonuniformly over about four porphyrins with most of the spin density on just two units even in the cyclic structures, in which all porphyrin sites are identical. Room temperature CW-EPR spectra indicate a larger spatial distribution of spin density on the EPR time scale. We introduce a combined molecular dynamics simulations and DFT approach to demonstrate that dynamic migration of delocalized polarons can occur in porphyrin oligomers and that this fully accounts for the apparent spin density distribution at room temperature. This method is a powerful tool in both the study and development of molecular wires and molecular electronics.

## Introduction

Organic semiconductors (OSC) have received much attention owing to their wide-reaching potential applications in photovoltaic devices, biological sensors and field-effect transistors.^1−3^ They span a range of charge-transport regimes depending on their charge-carrier mobility, with the two limiting cases being band-like ballistic transport for strongly delocalized carriers and slow hopping for localized carriers.^4,5^ The latter arises due to the lattice distortions and reorganization energy associated with the introduction of the charge carrier. The resulting quasi-particle comprised of charge carrier and lattice-distortions is called a polaron and controlling the extent of delocalization of polarons is important for tuning their mobility and therefore the conductivity of a material.^6,7^ Numerous classes of OSCs have been studied and most of them either act preferentially as p-type materials (hole polaron accumulation) or require specially controlled conditions (absence of both O_2_ and moisture) and low energy LUMOs in order to act as n-type material (electron polaron accumulation).^8^ Materials that are capable of n-type transport can often act as n- and p-type (ambipolar), which is an attractive property for applications such as light-emitting field-effect transistors.^8,9^ Therefore, establishing materials with similar mobilities of hole and electron polarons is an important area of exploration for OSC applications.^10^

Porphyrin systems have been explored as OSC materials with a focus on use as p-type layers^11−13^ and they provide insights into structure–property relationships through their synthetic versatility with scope for varying metals, linkers and anchoring groups.^14−16^ They have also been explored as promising candidates in the related area of single molecule field-effect transistors.^15,17,18^ Ethynylene-linked porphyrin chains have previously been investigated both in their oxidized and reduced forms: Rawson et al. used room-temperature CW-EPR and optical spectroscopy to study ethynylene-linked oligomers up to a length of seven porphyrin units and found electron polarons to be spread over the entire length of the molecules.^19^ Moise et al. reported a detailed EPR study on the cationic ethynylene-linked chains and demonstrated delocalization of charge carriers over at least five porphyrin units.^20^ In general, the relative mobility of hole or electron polarons in OSCs is highly system dependent.^10^ For example, Miller et al. found that electron polarons were more delocalized than hole polarons in materials such as poly(3-decylthiophene) (11.5 repeat units vs 8.7 repeat units respectively) but significantly less delocalized in others such as the donor–acceptor polymer F8BT (1 repeat unit vs 4 repeat units).^21,22^

**Figure 1:**
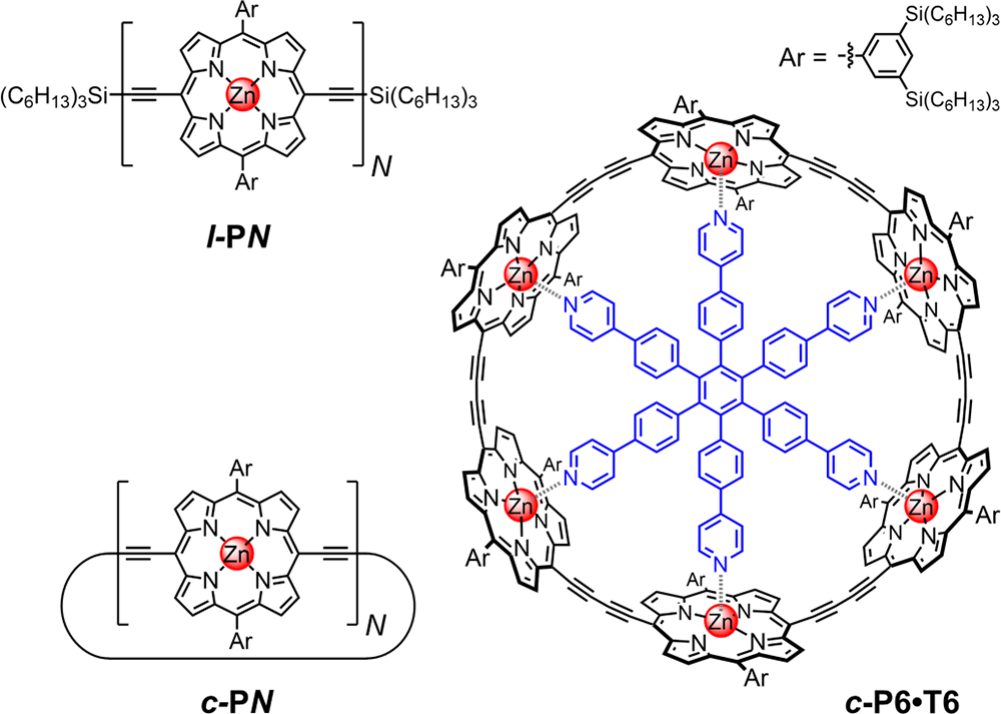
Structures of linear oligomers ***l*-P*N*** (*N* = 1–8), cyclic oligomers ***c*-P*N*** (*N* = 6, 12) and nanoring template complex ***c*-P6•T6**.

In this study, we explore the radical anions of butadiyne-linked porphyrin oligomers (see Figure 1), which were synthesized as previously reported.^23−26^ We employ a combination of spectroscopic and computational methods to determine the spatial distribution of the polarons and their dynamic behavior. We expand on the understanding of dynamic polaron migration by showing that the apparent delocalization from room-temperature EPR spectra can be fully accounted for by a dynamic hopping model. In addition, we revisit previous work by Peeks, Tait et al. on the radical cations of the same systems^27^ and explore the differences between hole and electron polarons in porphyrin molecular wires.

## Results and Discussion

### EPR Spectroscopy of the Porphyrin Monomer Anion

**Figure 2:**
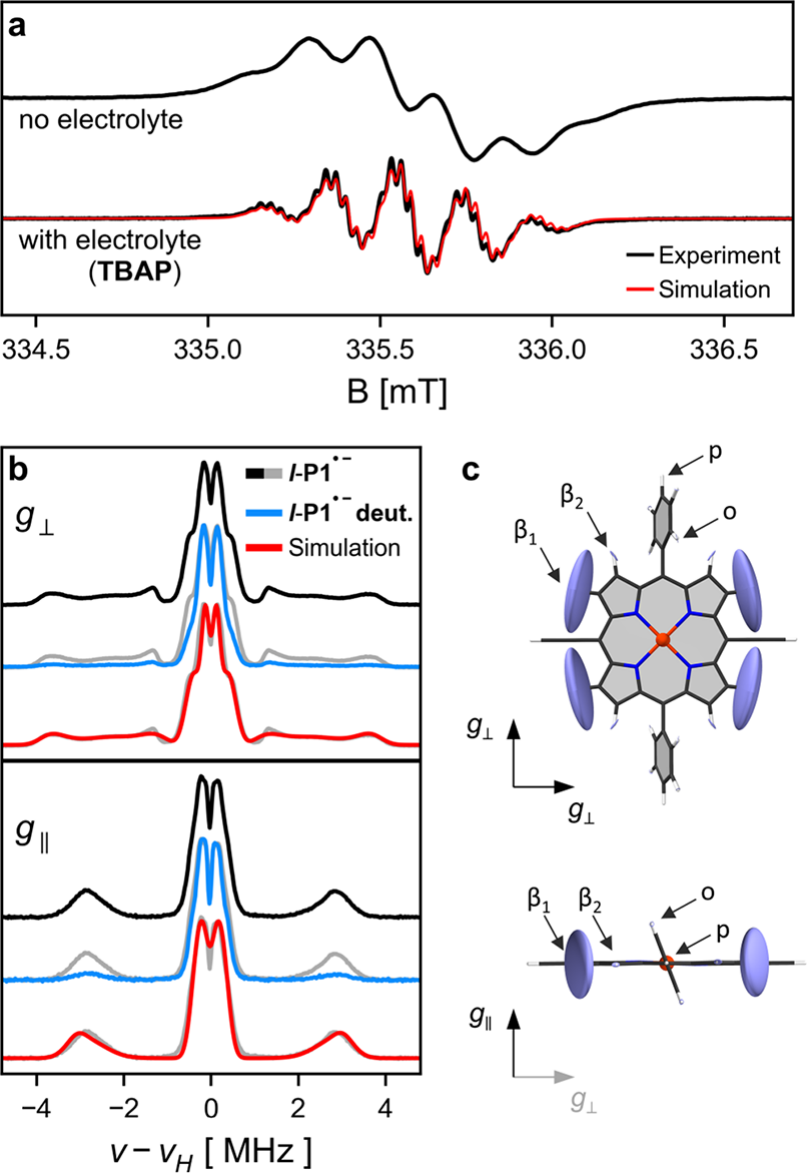
(a) CW-EPR Spectrum of ***l*-P1^•–^** recorded at 298 K at X-band frequencies in THF with and without electrolyte (TBAP). Parameters for simulation (red) are given in the SI. (b) ^1^H ENDOR spectra of ***l*-P1^•–^** recorded at 80 K in toluene-*d*_8_ at Q-band frequencies at fields corresponding to *g*_⊥_ and *g*_∥_. Spectra recorded on the partially deuterated structure (β_1_-H deuterated) are in blue and simulations based on DFT calculations in red. (c) DFT calculated hydrogen hyperfine coupling tensors with atom labels (o: *ortho*-H, p: *para*-H). Note that tensors for β_2_-, o-, and p-hydrogen atoms are very small and solubilizing chains have been replaced with hydrogen atoms.

Radical anions of various porphyrin derivatives and oligomers have previously been studied by continuous-wave EPR spectroscopy.^19,28,29^ We found that reduction of the porphyrin monomer ***l*-P1** with the one-electron reducing agent decamethyl cobaltocene CoCp_2_^*^ in THF yielded the previously reported CW-EPR spectrum^19^ of ***l*-P1^•–^** shown in Figure 2a (recorded at room temperature). This spectrum has not been interpreted in detail before and has an unexpected shape: the hyperfine pattern consists of six resolved, evenly spaced transitions (Figure 2a, top), although an even number of hyperfine coupling interactions (as would be expected in a symmetric molecule of point group D_2h_) can only give rise to an odd number of transitions. Addition of tetrabutylammonium hexafluorophosphate (TBAP), which acts as an inert electrolyte, substantially changes the spectral shape, resulting in a hyperfine pattern consisting of five major transitions and additional resolved hyperfine features (Figure 2a, bottom). This difference indicates that in the absence of TBAP a tight ion pair is formed between the porphyrin anion and the CoCp_2_^*+^ counterion, which is suppressed by the addition of electrolyte. The spectrum in the presence of electrolyte can now be simulated with four equivalent hydrogen nuclei with *A*_iso_ = 5.25 MHz and four equivalent nitrogen nuclei with *A*_iso_ = 0.80 MHz. Recording and simulating the correct, undistorted monomer spectrum is crucial for the quantification interpretation of trends for the longer oligomers.

The hyperfine couplings to hydrogen atoms play an important role when probing the extent of delocalization in the longer oligomers because they are the largest hyperfine interactions in these systems.^19^ They can be explored in more detail by ^1^H ENDOR spectroscopy and Figure 2b shows the ^1^H Mims ENDOR spectra of ***l*-P1^•–^** recorded at 80 K at *g*_⊥_ (in plane of the porphyrin) and *g*_∥_ (perpendicular to the plane of the porphyrin). Assignment of spectral features to certain hydrogen nuclei is possible from DFT calculations and spectral simulations using EasySpin.^30^ The largest hyperfine coupling arises from the β_1_-H adjacent to the acetylene linkers (Figure 2c). They are visibly separated from the remaining signal and in the case of *g*_⊥_ give rise to very broad features due to significant anisotropy. The slightly distorted shape of these wide signals arises from blind spots present in single-τ-value ^1^H Mims ENDOR spectra, which have been accounted for by summation of ^1^H ENDOR spectra recorded with different τ values and which are replicated in the spectral simulations. The isotropic value of these hyperfine couplings is 5.31 MHz in good agreement with the main hyperfine features in the room-temperature CW-EPR results. The central feature in the ^1^H ENDOR spectra is due to β_2_-H and *ortho* and *para* hydrogens on the phenyl groups. Their intensities had to be rescaled significantly in the simulations (see SI, Section 3.4) and in particular features close to 0 MHz had much too small intensities, presumably because THS solubilizing chains are replaced by hydrogen atoms in the DFT calculations.

^1^H ENDOR spectroscopy on a partially deuterated porphyrin monomer provides additional support for the assignment of hydrogen atoms. Deuteration of the β-H in the plane of the porphyrin results in removal of the spectral features at large hyperfine coupling values that have been assigned to β_1_-H (see Figure 2b). In addition, deuteration of these hydrogen atoms was found to increase *T*_m_ relaxation times by up to a factor of 2 (see SI, Section 4.2).

### EPR Spectroscopy of Porphyrin Oligomer Anions

**Figure 3:**
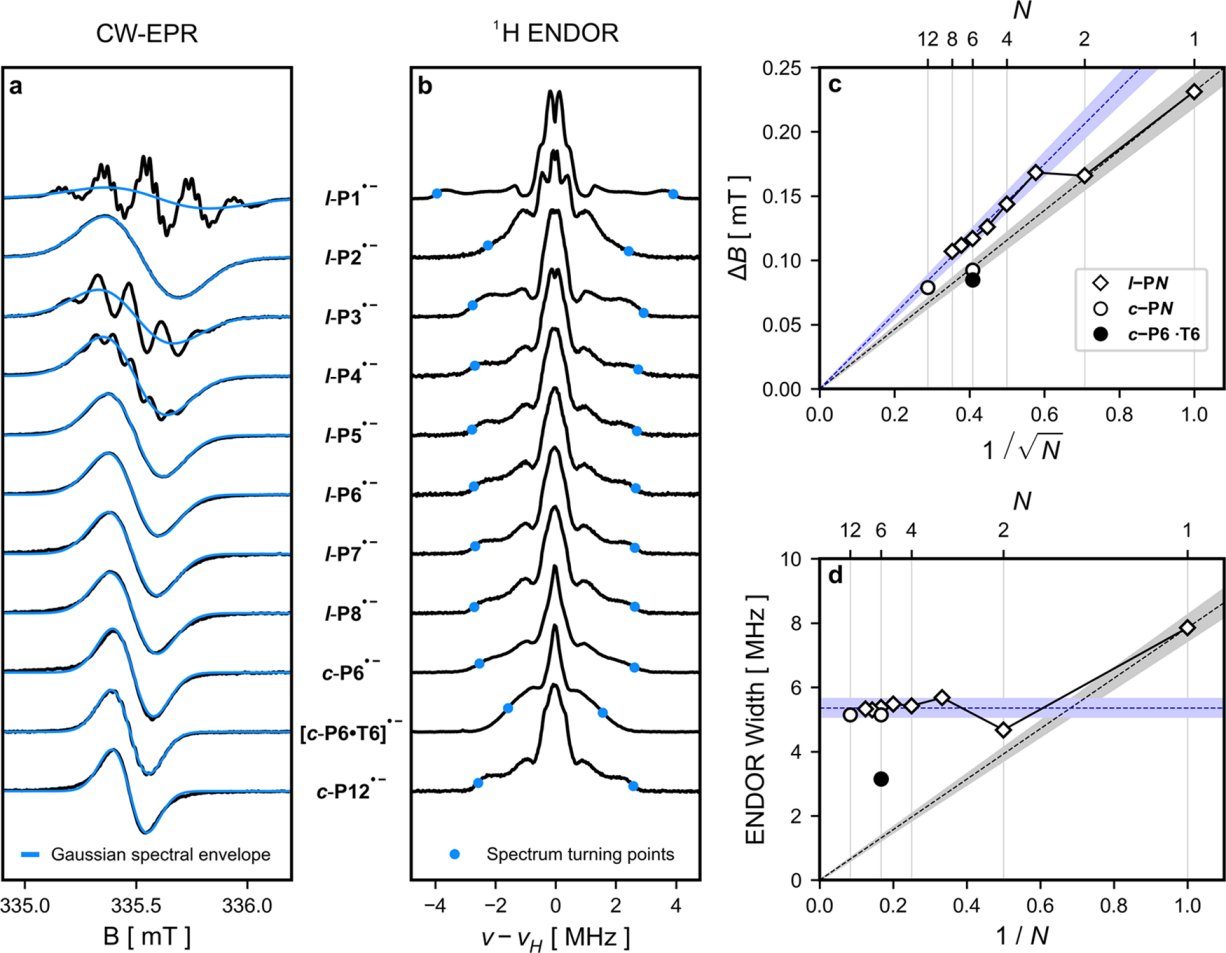
(a) CW-EPR spectra recorded at 298 K in THF with 10 mM TBAP at X-band frequencies (black) and fitted Gaussian derivative spectral envelope (blue). (b) ^1^H ENDOR spectra recorded at 80 K in toluene-*d*_8_ at Q-band frequencies at the field position with maximum signal intensity corresponding to *g*_⊥_. Blue markers indicate turning points of spectra. (c) Trend in CW-EPR spectral envelopes. (d) Trend in ^1^H ENDOR spectral widths. Black dashed lines show trend for full and uniform delocalization and blue dashed lines are fitted to the experimental trends for ***l*-P*N*^•–^** (*N* ≥ 3). Shaded areas indicate 95% confidence interval.

The room-temperature CW-EPR spectra of the series of linear oligomers ***l*-P*N*^•–^** (*N* = 1–8) and cyclic structures ***c*-P*N*^•–^** (*N* = 6, 12) are shown in Figure 3a. All spectra were recorded in THF with TBAP present (0.01 M) to suppress counterion effects, and a comparison of the spectra with and without electrolyte can be found in the Supporting Information. In order to avoid overreduction, samples were prepared by addition of substoichiometric amounts of CoCp_2_^*^. A general trend visible in these oligomeric systems is an increase in the number but a decrease in the magnitude of hyperfine couplings as the number of repeat units increases. This leads to broadened EPR spectra, and from ***l*-P5^•–^** onward hyperfine splittings are not resolved except for the highly symmetric, rigid **[*c*-P6•T6]^•–^** complex. The spectrum of ***l*-P2^•–^** appears to be anomalous as it does not display resolved hyperfine splittings but it is consistent with DFT calculations (see next section, Simulation and Modeling of Spin Densities).

The magnitude of the isotropic component of the hyperfine couplings is directly proportional to the amount of spin density at a given nucleus and therefore full and uniform delocalization of spin density over *N* repeat units is expected to decrease *A*_iso_ by a factor of 1/*N* relative to the monomer.^7^ For systems where the hyperfine couplings are not resolved, information about their relative size can be extracted from the width of the resulting spectral envelope. In the limit of complete and uniform delocalization on the time scale of the EPR measurement the width of the spectral envelope can be described by the Norris relationship:^20,31^
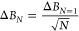
1where Δ*B*_*N*_ is the spectral envelope width of an oligomer with *N* repeat units and Δ*B*_*N*=1_ is the spectral envelope width of the monomer. The spectral envelopes of the CW-EPR spectra have been fitted as Gaussian derivatives and are plotted in blue in Figure 3a. The resulting spectral widths (Figure 3c) show that ***l*-P2^•–^** has  the spectral width of ***l*-P1^•–^** in agreement with the Norris equation, but overall, the linear oligomers do not follow the expected trend. However, after a discontinuity between ***l*-P2^•–^** and ***l*-P3^•–^**, there is an alternative linear trend that is consistent with the spectral width of ***l*-P3^•–^** as the base unit in the Norris equation:
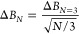
2In contrast, the spectral widths of the cyclic structures ***c*-P6^•–^** and **[*c*-P6•T6]^•–^** are in good agreement with the Norris prediction. Their spectral widths are very similar but **[*c*-P6•T6]^•–^** has resolved hyperfine splittings corresponding to 24 (=6 × 4) equivalent hydrogen nuclei (see SI, Section 1.3). The spectral width for ***c*-P12^•–^** roughly fits the linear trend set by the longer linear oligomers.

To study the behavior of the polarons in the absence of dynamic effects, we investigated the systems in frozen solution at 80 K.^32^ Broadening of the CW-EPR spectra due to *g*-anisotropy prevents any conclusions being drawn from those spectra (see SI, Section 1.6) and therefore, we again made use of ^1^H ENDOR spectroscopy (Figure 3b). The large number of hyperfine couplings present in these systems generally prevents definitive assignment of hyperfine couplings. However, similar to what is observed in the monomer, the largest hyperfine interactions that give rise to the broad shoulders in the spectra tend to be those to the β_1_-H atoms. The overall width of the ^1^H ENDOR spectra provide information about the largest hyperfine coupling present but due to the large anisotropy of the hyperfine interaction the widths cannot be obtained simply from the full width at half-maximum as done in previous studies.^7,27,33^ Instead, they were obtained from the turning points of the signals by picking the first maximum of the first derivatives of the ^1^H ENDOR spectra (see SI, Section 2.4).

Similar to the trend seen in the CW-EPR spectra, the low-temperature ^1^H ENDOR spectra show an approximate halving in the magnitude of the hyperfine couplings between ***l*-P1^•–^** and ***l*-P2^•–^** followed by a subsequent increase in ***l*-P3^•–^**. However, while the room-temperature CW-EPR spectra of the linear oligomers then showed a continuous decrease in the spectral width, the ^1^H ENDOR spectra approach a terminal width and no significant change is observed from ***l*-P4^•–^** onward. The nanoring anions ***c*-P6^•–^** and ***c*-P12^•–^** have similar ^1^H ENDOR spectral widths to the longer linear systems indicating the same polaron species. The more organized template complex **[*c*-P6•T6]^•–^** on the other hand has a narrower ^1^H ENDOR spectrum, which indicates a greater extent of delocalization of the polaron in this system.

Discrepancies between room-temperature CW-EPR and low-temperature ^1^H ENDOR trends have been interpreted previously in terms of a change in dynamic behavior of the polaron.^27,32,34^ At low temperatures, the polaron is coherently delocalized over a short distance and static, whereas at higher temperatures thermally activated hopping leads to a time-averaged spread of spin density over larger distances. Therefore, the terminal spectral width of the ^1^H ENDOR spectrum of ***l*-P4^•–^** together with the alternative Norris trend in the CW-EPR spectra originating from ***l*-P3^•–^** suggests that the polaron in the radical anions is delocalized over about three to four porphyrins in the linear and template-free cyclic porphyrin systems. The exact extent of delocalization of the polaron and its dynamic behavior are explored in more detail in the following section using theoretical modeling of the spin densities.

### Simulation and Modeling of Spin Densities

**Figure 4:**
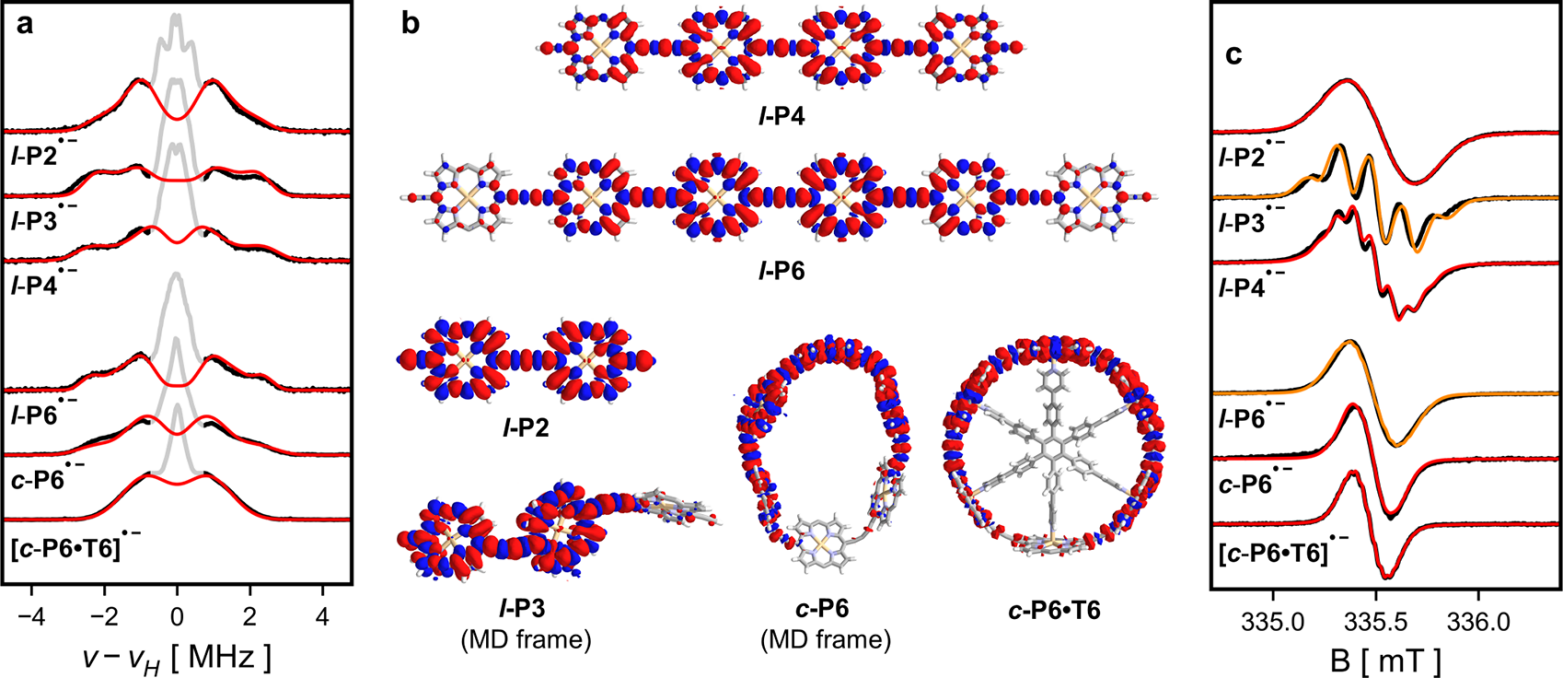
(a) Experimental ^1^H ENDOR of selected systems recorded at 80 K in toluene-*d*_8_ at Q-band frequencies (black) and simulations (red) from DFT calculated hyperfine couplings. Regions plotted in gray were omitted in the fitting. (b) Spin density plots of structures corresponding to simulations in (a). (c) Experimental CW-EPR spectra recorded at 298 K in THF with 10 mM TBAP at X-band frequencies (black) and simulations based on DFT calculations (red). CW-EPR simulations for ***l*-P3^•–^** and ***l*-P6^•–^** use hyperfine coupling values obtained from MD simulations (orange).

The experimental data can be explained using DFT calculations and Figure 4 shows simulations of CW-EPR and ^1^H ENDOR spectra for selected systems. The choice of DFT functional is crucial for the correct description of the spin density, as self-interaction errors (SIE) can significantly impact the delocalization lengths of electrons.^35^ For this reason the range separated functional lc-ωPBE was chosen since it has low amounts of SIE and has been shown to give good agreement with experiments for similar systems.^19,27,36^ In addition, it only has a single empirical parameter ω,^37^ which determines over which range exact exchange is used, and tuning it to the experimental data provides a systematic way to computationally investigate delocalization lengths. Different spin densities were calculated on the optimized structures for values of ω between 0.075 and 0.225. Values for isotropic hyperfine constants and hyperfine tensors were then taken directly from DFT calculations and spectra were simulated using EasySpin.^30^ The simulations of ^1^H ENDOR spectra were limited to β hydrogen atoms, which contain the most relevant information about the extent of delocalization and can be assigned to the largest spectral features (see SI for details of the simulations and simulations of full spectrum, Section 3).

Given the conflicting experimental trends, it is not possible to simulate both the room-temperature CW-EPR and low-temperature ^1^H ENDOR spectra of larger systems using the same spin density. Good agreement with low-temperature ^1^H ENDOR measurements was found for ω values of 0.200 or 0.225 and the corresponding spin densities are shown in Figure 4b. For ***l*-P*N*^•–^** structures with *N* ≥ 4, the spin density is delocalized nonuniformly over about four porphyrin units with most of the spin density (>75%) spread over only two porphyrins. The only structure with a larger extent of delocalization is the template-bound ring **[*c*-P6•T6]^•–^**, in which the spin density is spread more evenly over three porphyrins.

In contrast, the room-temperature CW-EPR spectra could only be simulated with more delocalized spin density distributions obtained from DFT calculations with ω ≤ 0.100. An increase in apparent time-averaged delocalization at room temperature has previously been assumed to arise from dynamic polaron migration along the porphyrin chains,^27,34^ but this has not been explored in detail so far. Ab initio molecular dynamics (MD) simulations can provide insights into these dynamic effects,^5,38^ but reaching the time scale relevant for EPR spectroscopy is prohibitively expensive for such large structures. To investigate the dynamic effects in a computationally efficient way, we carried out multiscale simulations in two steps: a molecular dynamics trajectory was created at a lower semiempirical level of theory (xTB) to sample a range of geometries and afterward higher level of theory (DFT) single point calculations were computed on structures chosen at equally spaced time intervals. The single point calculations were done using lc-ωPBE with ω = 0.200 as it gave a good description of static properties for low-temperature ^1^H ENDOR spectra. The molecule ***l*-P6^•–^** was chosen for this simulation as it is large enough to display appreciable dynamic migration effects given a polaron delocalization length of ≤4 porphyrins but also not as computationally demanding as the longest oligomers. This simulation methodology has been previously used to study the structural dynamics of localized spin labels.^39,40^ Here, we demonstrate the suitability of this approach for the investigation of synthetic nanomaterials with delocalized spin distributions.

**Figure 5:**
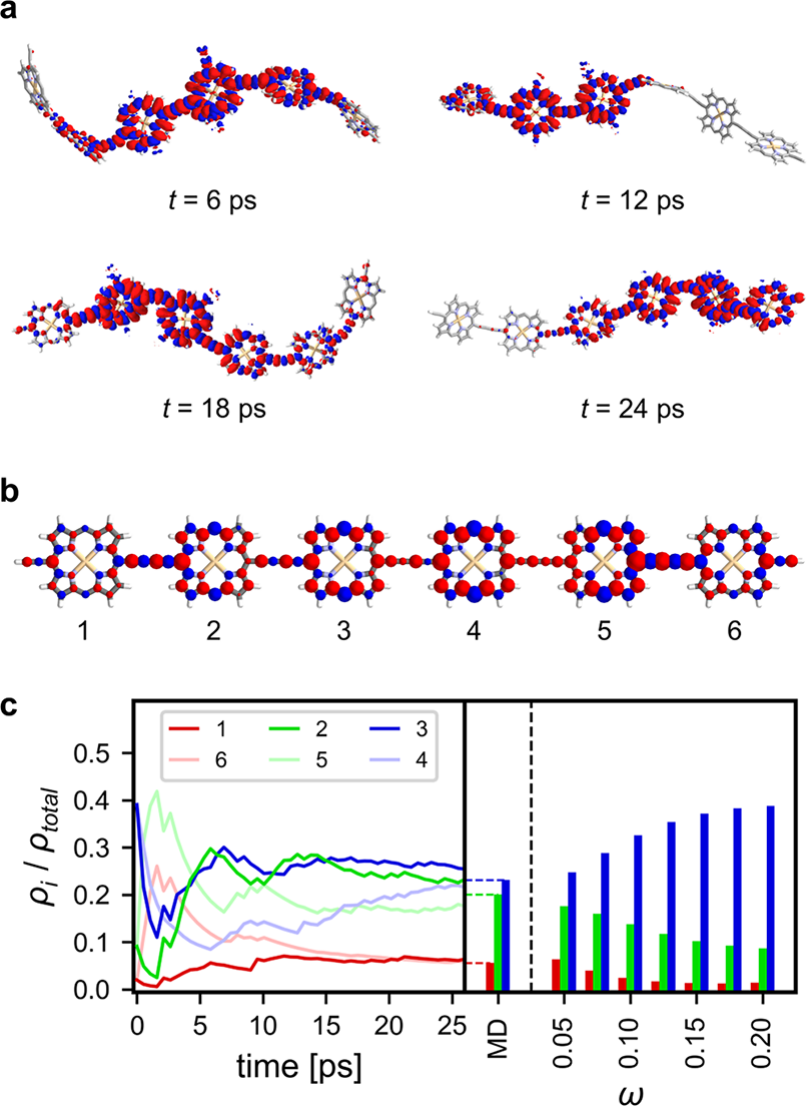
(a) Structures and spin density distributions at selected time points during an MD simulation of ***l*-P6^•–^**. (b) Average Mulliken spin populations over the time scale of the simulation. (c) Left: Plot of the running averages of the relative spin densities on each porphyrin unit. Right: Bar chart of relative spin densities calculated either by MD or from lc-ωPBE with different values of ω. Optimized geometry of ***l*-P6^•–^** was used as the starting point of the MD simulation.

**Figure 6:**
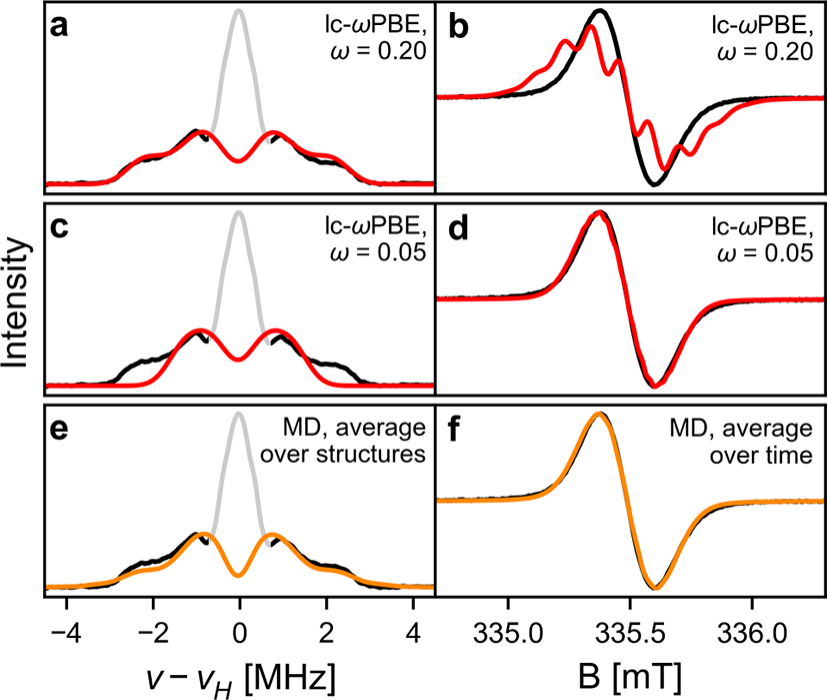
^1^H ENDOR (a,c,e) and CW-EPR (b,d,f) spectra of ***l*-P6^•–^** (black) and simulations based on single-point DFT calculations (red) and MD simulations (orange). For the ^1^H ENDOR simulation in plot (e), spectra were simulated for each structure sampled in the MD simulation individually and then averaged. CW-EPR simulation in plot (f) used the average hyperfine couplings over the course of the MD simulation for each nucleus.

During the MD simulations, spin density visibly moves along the entire chain (see Figure 5a and SI, Section 3.2) confirming the dynamic behavior of the polarons even in these moderate sized structures. The relative amounts of spin densities on a given porphyrin ρ_*i*_ are plotted as a running average in Figure 5c. The resulting time-averaged spin density is much more spread out over the chain (Figure 5b, cf. ***l*-P6^•–^** in Figure 4b) and the hyperfine coupling constants derived from the MD simulation give a good fit to the experimental CW-EPR spectrum (Figure 6f). In addition, the average of the simulated ^1^H ENDOR spectra using hyperfine interactions of the individual frames also is in good agreement with the experimental low-temperature ^1^H ENDOR spectrum (Figure 6e). Despite the dynamic polaron motion, end groups still have noticeably less spin density than the central porphyrin units, which is consistent with the deviation of the CW-EPR spectral envelope widths of ***l*-P*N*^•–^** from the ideal Norris trend. Incomplete delocalization on the terminal porphyrin units is also supported experimentally by the difference between ***l*-P6^•–^** and the cyclic systems ***c*-P6^•–^** and **[*c*-P6•T6]^•–^**. The latter follow the expected Norris trend since all porphyrin units are equivalent. The cyclic structure ***c*-P12^•–^** deviates from the Norris trend, which may be due to incomplete time averaging on the EPR time scale or because of slow tumbling, both caused by the large size of this system.

The average spin density of the MD simulation of ***l*-P6^•–^** approaches the one calculated on the optimized structure using lc-ωPBE with ω = 0.05 (Figure 5c). While this spin density cannot account for the frozen solution ^1^H ENDOR spectrum (Figure 6c), it gives a similarly good fit for the CW-EPR spectral shape of ***l*-P6^•–^** (Figure 6d). This shows that lc-ωPBE calculations with lower values of ω lead to spin densities that implicitly capture the dynamic averaging effect. They can therefore provide a computationally cheaper way to describe the average spin densities of ***l*-P*N*^•–^** and simulate CW-EPR spectra (as shown in Figure 4), although one needs to be careful not to interpret this as real coherent delocalization over larger distances.

While using the DFT optimized structure and adjusting the value of ω in the range separated functional gives a reasonable description of the spin density in ***l*-P6^•–^**, this does not necessarily work well for all systems. For example, the best ^1^H ENDOR simulation fits tended to be less good for odd numbered porphyrin oligomers compared to even numbered ones (see SI, Section 3.4). Considering the strong similarity between the experimental ^1^H ENDOR spectra of even and odd numbered porphyrin oligomers, this suggests that structural dynamics may break the symmetry of the systems, which is visible in the MD simulations. This mainly changes the position and symmetry of the polaron species but not the overall spatial extent: comparing the spin densities of the optimized structure of ***l*-P6^•–^** with those of structures sampled during the MD simulations shows that there is only a small decrease in the delocalization length (see SI, Section 3.5). This symmetry breaking effect is seen particularly well in ***l*-P3^•–^**. Slight structural distortions cause the spin density to mainly spread over two of the three porphyrin units (see Figure 4b), which results in hyperfine couplings in much better agreement with experimental spectra.

Dynamic effects play a role even in systems with a fully delocalized spin density such as ***l*-P2^•–^**. Surprisingly, the room-temperature CW-EPR spectrum and low-temperature ^1^H ENDOR spectrum of ***l*-P2^•–^** could not be simulated with the same spin density. MD simulations of ***l*-P2^•–^** show that even in the dimer the spin density is affected by structural dynamics. Although the two porphyrin units remain almost coplanar (dihedral angle <40°), the spin density shifts between them, leading to an averaging of hyperfine coupling constants (see SI, Section 3.3). The room-temperature CW-EPR spectrum of ***l*-P2^•–^** does not show any resolved hyperfine splittings, even though ***l*-P4^•–^**, which has the same symmetry, does. This is consistent with DFT calculations which show that the central eight β_1_-H in ***l*-P4^•–^** have almost identical coupling constants (4 H with *A*_iso_ = 2.24 MHz and 4 H with *A*_iso_ = 2.30 MHz) whereas the corresponding hydrogen atoms in ***l*-P2^•–^** have a larger difference (4 H with *A*_iso_ = 2.38 MHz and 4 H with *A*_iso_ = 2.74 MHz). Given the same line width, this larger inhomogeneity of hyperfine coupling constants leads to a broadening out of the splittings seen in the CW-EPR (see SI, Section 3.3).

### Optical Spectroscopy

**Figure 7:**
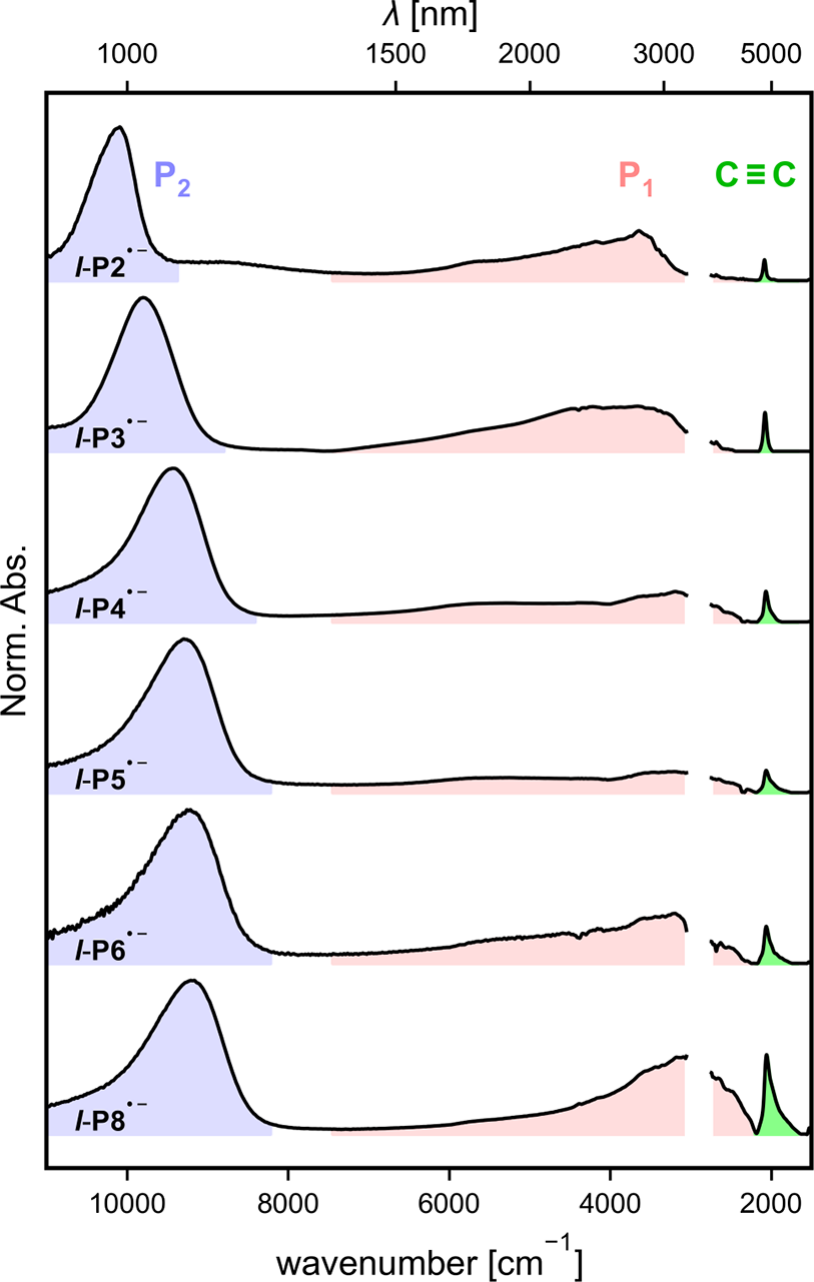
NIR/MIR spectra of ***l*-P*N*^•–^** radical anions, recorded in THF containing 0.1 M TBAP at 298 K. Gap between 2750 and 3050 cm^–1^ is due to overlap with vibrational modes of the solvent.

Further evidence of the extent of delocalization of the charge carriers was obtained from room-temperature near-infrared (NIR) optical spectroscopy data. Unlike CW-EPR spectroscopy, optical measurements probe the electronic structure on a faster time scale than dynamic polaron migration and therefore provide information on their coherent delocalization length. Absorption spectra of ***l*-P*N*** were obtained while varying the applied electrochemical potential in THF with 0.1 M TBAP as supporting electrolyte. Square-wave voltammetry in the same solvent system was performed prior to spectro-electrochemical measurements to determine the first reduction potentials, which decrease with oligomer length (see SI, Section 5). The potential was swept to a state corresponding to one charge per porphyrin and yielded a 2D plot of overlapping spectra of different oxidation states (see SI, Section 6). These were then deconvoluted to obtain distinct spectra of the individual oxidation states. Figure 7 shows the spectra for the monoanions after subtraction of the spectra of solutions before reduction to remove solvent peaks. The spectrum of ***l*-P1^•–^** is omitted from Figure 7 as it does not have any absorption bands in the NIR region at λ ≥ 1000 nm.

**Figure 8:**
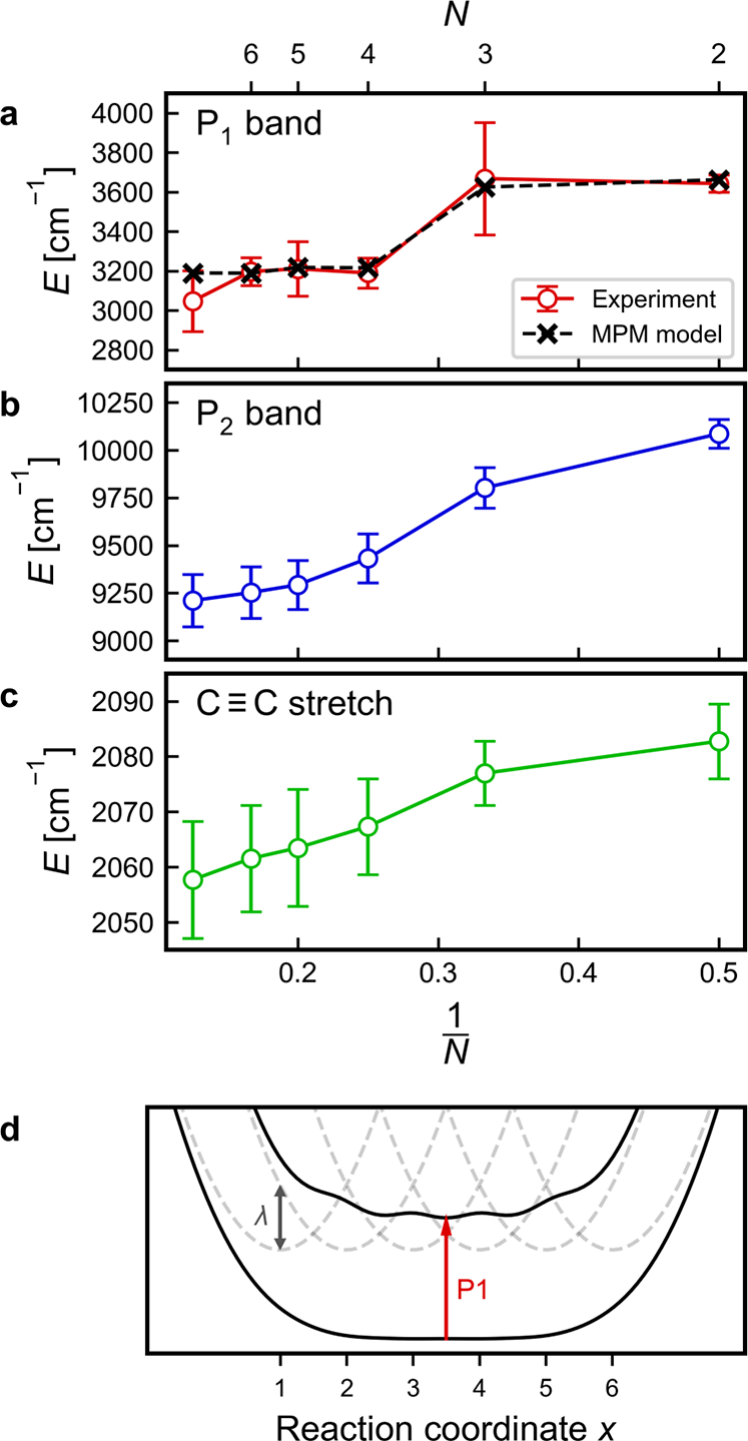
(a–c) Trends in transition energies for characteristic bands in NIR/MIR spectra. Error bars show 95% confidence interval. (d) Potential curves of the MPM model for six sites showing the ground and first excited states in black and the uncoupled diabatic potentials in gray.

The NIR spectra show two intense absorption bands, labelled P_1_ and P_2_, with maxima in the regions around 3000–5000 cm^–1^ and around 8000–10,500 cm^–1^, respectively, similar to those seen in the radical cations of butadiyne-linked porphyrin oligomers and radical anions of ethynylene-linked porphyrin oligomers.^19,27^ These arise predominantly from SOMO → LUMO and HOMO → SOMO transitions for P_1_ and P_2_, respectively.^27,41^ Both of these bands clearly shift to lower energies in ***l*-P*N*^•–^** for larger *N* consistent with a decrease in the optical excitation gap due to increased delocalization. The transition energies are plotted against 1/*N* in Figure 8a–c with error bars showing a 95% confidence interval. These plots would show a linear relationship for a simple particle in a box model. The P_1_ transition energy saturates at ***l*-P4^•–^** (Figure 8a) with no further shift seen in ***l*-P5^•–^** and ***l*-P6^•–^** which indicates an effective conjugation length of four porphyrins in good agreement with ENDOR measurements. The P_1_ transition energy of ***l*-P3^•–^** is very similar to that of ***l*-P2^•–^** although the uncertainty is large due to the peak being very broad. The P_2_ band has not fully saturated yet even for ***l*-P8^•–^** (see Figure 8b). However, the P_1_ band is expected to be a better indicator of the polaron delocalization as TD-DFT calculations predict it to be a more pure SOMO transition than P_2_ (see SI, Section 6.3).

Marcus–Hush theory and the multiparabolic model (MPM)^27,42−44^ provide a simple model to describe charge delocalization in mixed-valence compounds in which each site of a multisite system is described by a harmonic potential well that couples to its adjacent sites. The potential wells are defined along a reaction coordinate *x* that describes the positions of the different sites along a chain with multiple sites. The extent of delocalization depends on the magnitude of the coupling between sites *H*_ab_ and the reorganization energy λ. The ratio between them can be used to classify systems into different mixed-valence regimes. The overall Hamiltonian matrix *H*(*x*) can be written as
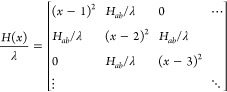
3and diagonalization of *H*(*x*) at every position along *x* yields the adiabatic potential curves for the ground and excited states. This is illustrated for the case of ***l*-P6^•–^** in Figure 8d. The difference between the energy minima of the ground state and the first excited state corresponds to the lowest optical excitation energy. Therefore, in the case of the radical anions of ***l*-P*N*^•–^**, the P_1_ band can be used to determine the ratio *H*_ab_/λ. The multiparabolic model was fitted to the experimental data as shown in Figure 8a. The best fit (RMSD = 24.3 cm^–1^) was found for a ratio *H*_ab_/λ of 0.83, which places the radical anions in the Robin-Day class III of mixed-valence compounds (full mixing of potential energy curves, *H*_ab_/λ > 0.5) for coupling between neighboring porphyrin centers. This classification is also supported by the full delocalization of spin density seen in the dimer ***l*-P2^•–^**.

In addition to the P_1_ and P_2_ bands, the spectra show greatly amplified intensities of the C≡C stretch compared to the neutral oligomers due to mixing of electronic and vibrational transitions.^45^ They follow a similar trend to the optical transitions in shifting to lower energies for longer oligomers (Figure 8c) which can be interpreted as an increasing cumulene character of the butadiyne linker. Just like the P_2_ band, it does not reach the saturation in the investigated oligomers (see Figure 8b).

### Comparison between Anions *l***-P***N*^•–^ and Cations *l***-P***N*^•+^

The optical spectra of ***l*-P*N*^•–^** have a very similar structure to their cationic analogues ***l*-P*N*^•+^**.^27^ This result is in agreement with Arnold et al., who showed that there is a remarkable similarity between the NIR spectra of the anion and cation of a closely related butadiyne-linked porphyrin dimer due to the energetically symmetric electronic structure of the frontier-orbitals.^41^ Here we show that this homology continues for longer butadiyne-linked oligomers, which have a similarly symmetric electronic structure to the dimer (see SI, Sections 3.6 and 6.3). In addition, the ratio of *H*_ab_/λ = 0.83 obtained from a simple multiparabolic model for ***l*-P*N*^•–^** places them in Robin-Day class III similar to the cations.

**Figure 9:**
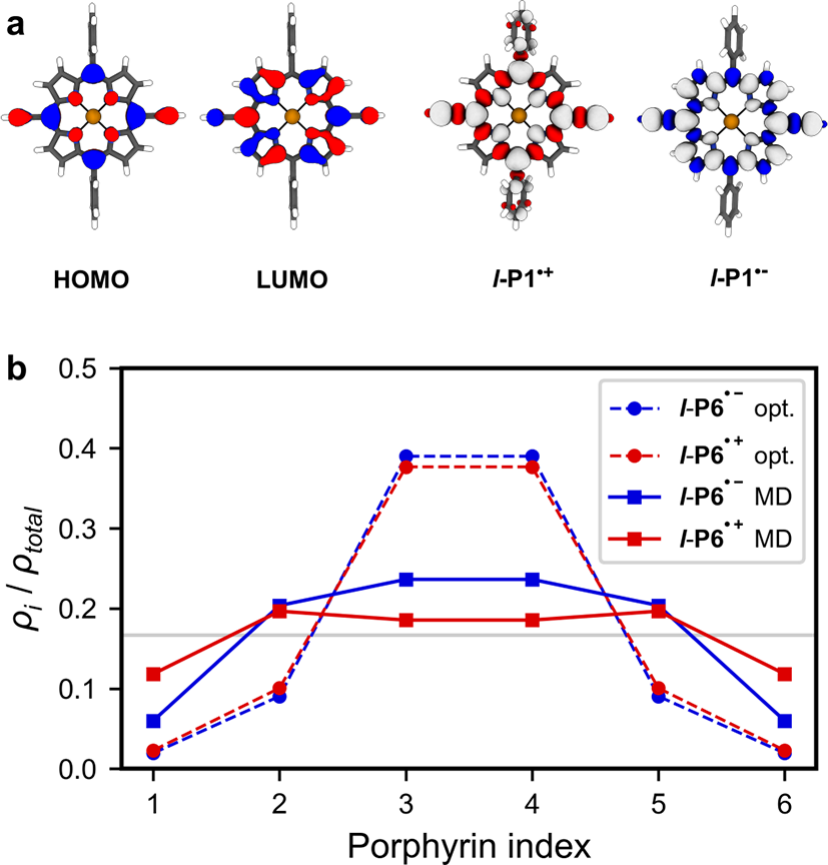
(a) Frontier orbitals of ***l*-P1** (isosurfaces at ±0.03) and spin densities of the cation ***l*-P1^•+^** and anion ***l*-P1^•–^**. (b) Relative spin densities on each porphyrin in the radical anion and cation of ***l*-P6** for optimized structures and molecular dynamics time average. Gray line shows spin density for full and uniform delocalization. Porphyrin indices are defined as shown in Figure 5b.

Considering the close similarity of the optical data for ***l*-P*N*^•–^** and ***l*-P*N*^•+^**, the difference in their EPR spectra is remarkable. Compared to their cationic counterparts, the ^1^H ENDOR spectra of ***l*-P*N*^•–^** give rise to significantly more fine-structure and larger values of hyperfine couplings. These differences in the hyperfine couplings can be inferred from the frontier orbitals of the neutral porphyrin monomer that give rise to the SOMO in the radical anions and cations (see Figure 9a). The HOMO of a porphyrin with D_2h_ symmetry has negligible density on the outer carbon atoms of the porphyrin ring, resulting in small hydrogen hyperfine coupling constants to the β_1_-H atoms in ***l*-P*N*^•+^**. In contrast, the LUMO has less spin density in the center and more spin density on the outer ring, giving the observed large hydrogen hyperfine couplings and smaller nitrogen hyperfine coupling interactions in ***l*-P*N*^•–^**. However, the overall trends in ^1^H ENDOR widths are comparable for ***l*-P*N*^•–^** and ***l*-P*N*^•+^** indicating similar delocalization lengths of the static polarons and for ***l*-P6** the anion and cation have almost identical spatial extents of the spin density distributions (Figure 9b).

Similarly to the anionic systems, a continuous narrowing of the spectral envelope widths of the CW-EPR spectra was found for the cations ***l*-P*N*^•+^**.^27^ As for the anionic systems, we applied our Molecular Dynamics approach introduced above to model the dynamic spin density behavior of the porphyrin cations. The results illustrate that the trend in the room-temperature CW-EPR spectra of the cations also arises due to dynamic polaron migration. However, while the anionic systems ***l*-P*N*^•–^** studied here have a discontinuity in the spectral envelope widths, the line widths of the CW-EPR spectra of the cations ***l*-P*N*^•+^** follow the expected Norris relationship for all oligomers. As described above, the discontinuity seen in the trend of the anions ***l*-P*N*^•–^** is likely to arise from incomplete delocalization of spin density onto terminal groups, but the time averaged spin density for ***l*-P6^•+^** derived from molecular dynamics simulations does have more spin density on the terminal porphyrin units and less on the central two porphyrins compared to ***l*-P6^•–^** (see Figure 9b). The spin density in ***l*-P6^•+^** therefore more closely resembles uniform spin density distribution over the entire porphyrin chain on the EPR time scale and consequently gives a better match with the Norris equation (see SI, Section 3.3). The origin of the apparent differences in CW-EPR trends for ***l*-P*N*^•–^** and ***l*-P*N*^•+^** can therefore be explained using our approach of combining molecular dynamics simulations and DFT calculations.

## Conclusions

In this study, we investigated the extent of delocalization in the radical anions of butadiyne-linked porphyrin oligomers through a range of spectroscopic and computational techniques. The widths of ^1^H ENDOR spectra start to deviate from the expected trend for full and uniform delocalization after the dimer ***l*-P2^•–^** and fully saturate from the tetramer ***l*-P4^•–^**. Along with DFT calculations, these results indicate that the polaron is delocalized nonuniformly over four porphyrins with most of the spin density distributed over just two repeat units.

Dynamic polaron migration, or hopping, has previously been proposed as the reason for the apparent increased delocalization in room-temperature CW-EPR spectra.^27,34,46^ Here we have employed multiscale simulations using semiempirical xTB molecular dynamics together with DFT single point calculations to confirm this behavior. We furthermore validate the power of this approach by applying it to the previously studied hole polarons of butadiyne-linked porphyrin oligomers.

In addition to the linear oligomers, three cyclic systems were investigated. The template-free structures ***c*-P6^•–^** and ***c*-P12^•–^** have very similar ^1^H ENDOR spectra to the longer linear oligomers indicating the same extent of delocalization of the polaron species. In contrast, **[*c*-P6•T6]^•–^** has a narrower spectral width consistent with a more delocalized spin density with about equal spread of spin density over three porphyrins due to lower conformational disorder.

Optical spectroscopy on the linear oligomers shows two intense absorption bands in the NIR region. The lowest energy absorption band P_1_ saturates from ***l*-P4^•–^** indicating a polaron delocalization length of four porphyrin units in good agreement with the EPR results.

The electron polarons in butadiyne-linked porphyrin oligomers show a similar spatial extent of delocalization compared to the hole polarons^27^ studied previously. Despite this, various differences arise in CW-EPR and ^1^H ENDOR spectra, which can only be fully explained by careful spectral simulation using density functional theory calculations and molecular dynamics simulations. The delocalization lengths of anionic and cationic polarons in butadiyne-linked porphyrin oligomers are longer than those for polarons in most other types of conjugated polymers, but they are shorter than polarons in edge-fused porphyrin nanoribbons.^33^ These results establish butadiyne-linked porphyrin oligomers as ambipolar materials and show that the extent of delocalization depends more on the identity of the interporphyrin-linkers than the sign of the charge of the polaron in these systems.

## Supporting Information Available

Details on spectroscopy, theoretical calculations, data analysis and simulation methods, additional UV–Vis, IR, and EPR spectra (PDF)
