# Supplementary material for: Radical Anions of Porphyrin Molecular Wires: Delocalization and Dynamics
Source: J Am Chem Soc. 2024 Dec 30;147(1):978–87. doi: 10.1021/jacs.4c14161 (PMC11726548; doi:10.1021/jacs.4c14161)
Supplement: Supplementary file 1 — ja4c14161_si_001.pdf [file ja4c14161_si_001.pdf]

Supporting Information

Radical Anions of Porphyrin Molecular Wires: Delocalization and Dynamics

Janko Hergenhahn,[†,‡] Jake M. Holmes,[‡] Jie-Ren Deng,[‡] Henrik Gotfredsen,[‡] Robert M. J. Jacobs,[‡] Sebastian M. Kopp,[*,†,‡] Christiane R. Timmel,[*,†,‡] and Harry L. Anderson[*,‡]

[†]Centre for Advanced Electron Spin Resonance, Department of Chemistry, University of Oxford, Oxford, OX1 3QR, UK
[‡]Chemistry Research Laboratory, Department of Chemistry, University of Oxford, Oxford, OX1 3TA, UK

Contents

1. Continuous wave (CW) EPR 3
   1.1. Methodology 3
   1.2. Spectra of *l*-P$N^{\bullet-}$ in different solvent systems 3
   1.3. Best fit spectral simulations 4
   1.4. Gaussian envelope fit 5
   1.5. Voigt profile fit 6
   1.6. Low temperature CW-EPR 8
2. Pulsed EPR spectroscopy 9
   2.1. Methodology 9
   2.2. Echo detected field sweep spectra 9
   2.3. ENDOR spectra at $\boldsymbol{g}\ \perp$ and $\boldsymbol{g}\ \parallel$ 11
   2.4. ENDOR derivatives used to estimate spectral widths 12
3. Modelling and Simulation 14
   3.1. Methodology 14
   3.2. Simulations 15
   3.3. CW-EPR 17
   3.4. ENDOR 21
   3.5. Quantifying Delocalization 25
   3.6. Electronic structure of the neutral molecules 26
4. Radical anions of partially deuterated porphyrins 27
   4.1. Synthesis 27
      4.1.1. Synthesis of *d*-H_2P1_{THS} 27
      4.1.2. Synthesis of *d*-$ZnP1_{THS}$ 28
      4.1.3. Synthesis of *d*-$ZnP1_{THS}$-Br_2 28
      4.1.4. Synthesis of *d-ZnP1*$_{THS}$-$CPDIPS_2$ 29
      4.1.5. Synthesis of *d*-*lb*-ZnPN_{THS}-e_1 30
      4.1.6. Synthesis of *d*-*lb*-ZnPN_{THS}-CPDIPS 31
   4.2. EPR measurements 36
5. Square wave voltammetry 38
6. Spectroelectrochemistry 39
   6.1. Methodology 39
   6.2. Spectra 40
   6.3. Comparison of transitions in *l*-P$N^{\bullet-}$ and *l*-P$N^{\bullet+}$ 43
7. References 46

1. Continuous wave (CW) EPR

1.1. Methodology

Samples of ***l*-P*N*** (*N*=1-8), ***c*-P6**, ***c*-P12** and ***c*-P6•T6** were synthesized as reported previously. [1, 2, 3, 4] Samples for CW-EPR measurements were prepared under an inert nitrogen atmosphere inside a glove box (MBraun LabStar Glovebox) by addition of 0.5 equivalents of **CoCp*$_2$** to a solution of porphyrin oligomer (200 µM). The **CoCp*$_2$** was sublimated in a sublimation flask on a Schlenk line prior to cycling it into the glove box where tetrahydrofuran (THF) was added to make an approximately 10 mM solution. The concentration of the solution was then validated on an aliquot of the solution by quantitative NMR with t-butyl benzene as a reference. Different solvent systems were tested for the CW-EPR measurements including toluene, THF and THF with 10 mM concentration of $[Bu_4N][PF_6]$ as inert electrolyte. Solvents were filtered over dry alumina and purged of oxygen before cycling them into the glove box. Samples were kept under inert atmosphere by using X-band EPR tubes with a J.Young cap.

CW spectra were recorded at X-band frequencies on a Bruker Biospin EMX spectrometer with a high sensitivity Bruker probe head at room temperature. Modulation amplitudes were adjusted until the smallest features could be resolved and no longer changed in shape and ranged between 0.005 mT and 0.02 mT. Similarly, powers were adjusted until no broadening effects were observed. This was the case for ***l*-P1**$^{\bullet-}$ at 2.0 mW (corresponding to a power attenuation of 20 dB on our spectrometer) and ranged between 0.1 and 0.2 mW for all longer systems (corresponding to 30 to 33 dB). The temperature for low temperature CW-EPR measurements was controlled with an Oxford Instruments digital temperature controller ITC503s and an Oxford Instruments helium-flow cryostat ESR900 using liquid nitrogen as cryogen. Simulations of EPR spectra were done in MATLAB using the garlic function from the EasySpin toolbox.

1.2. Spectra of *l*-P*N*$^{\bullet-}$ in different solvent systems

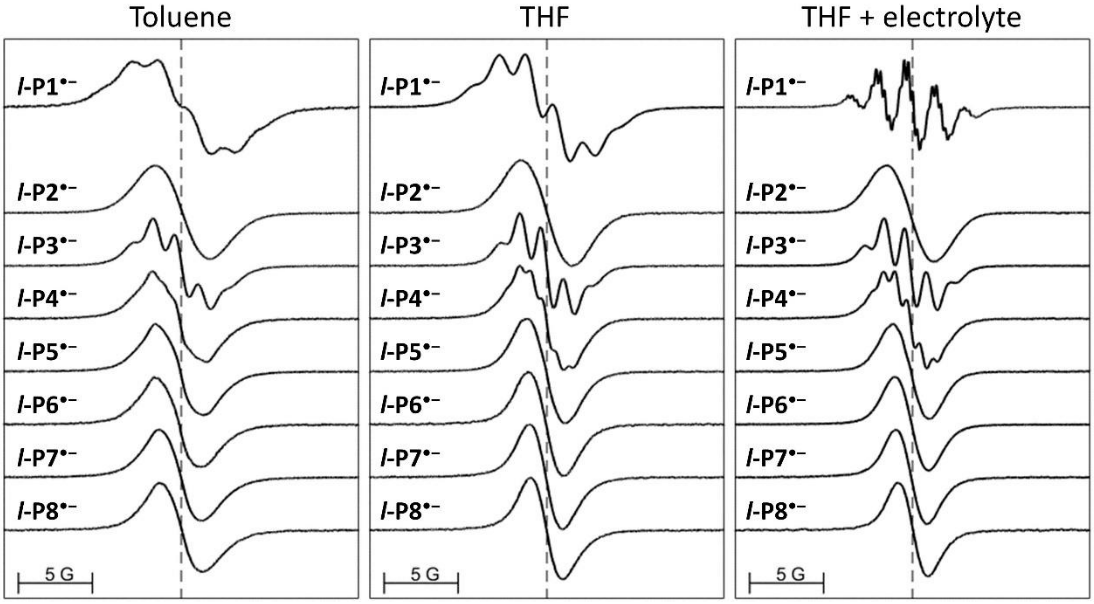

Figure S1-1. CW-EPR spectra recorded at 298 K at X-band frequencies under different solvent conditions. Spectra have been aligned to centre for better comparison.

1.3. Best fit spectral simulations

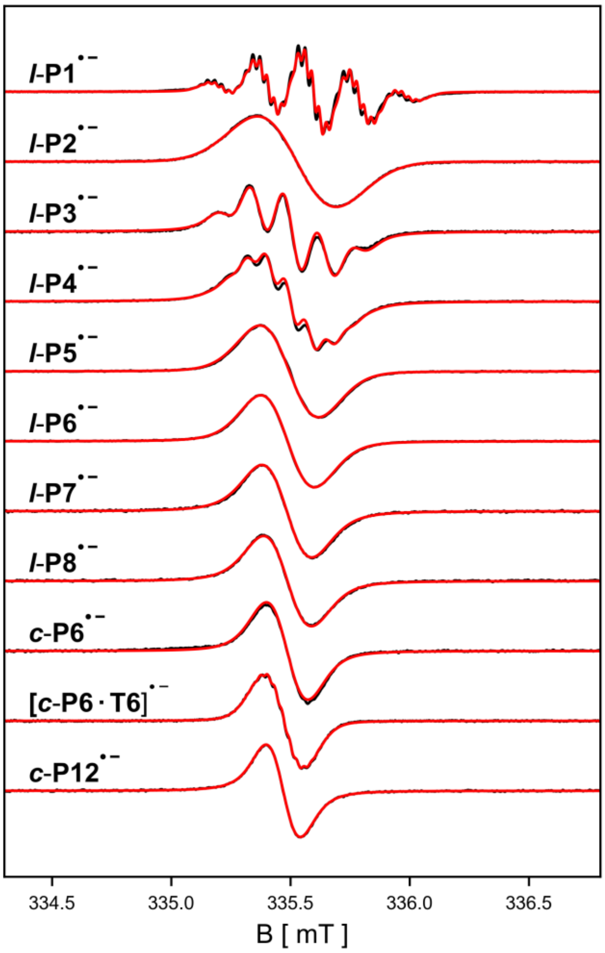

Figure S1-2. CW-EPR spectra (black) recorded in THF with 10 mM $[Bu_4N][PF_6]$ at room temperature at X-band frequencies and simulations (red). Spectra are aligned to 9.4 GHz.

Table S1-1. CW-EPR simulation parameters.

Structure	*g*	Gaussian linewidth / mT	Lorentzian linewidth / mT	Nuclei	*N*	A_{iso} / MHz
***l*-P1•−**	2.0012	0.0014	0.0306	H	4	5.28
				N	4	0.81
***l*-P2•−**	2.0017	0.3245	0.0109			
***l*-P3•−**	2.0018	0.0605	0.0683	H	4	3.79
***l*-P4•−**	2.0018	0.0329	0.0701	H	8	2.24
***l*-P5•−**	2.0018	0.2255	0.0386			
***l*-P6•−**	2.0019	0.1944	0.0559			
***l*-P7•−**	2.0019	0.1615	0.0826			
***l*-P8•−**	2.0019	0.1579	0.0755			
***c*-P6•−**	2.0019	0.1361	0.0663			
**[*c*-P6•T6]•−**	2.0020	0.0182	0.0309	H	24	0.83
***c*-P12•−**	2.0020	0.1012	0.0722			

The hyperfine couplings that could be resolved in **[*c*-P6•T6]$^{•-}$** are very small and it is not immediately clear what the absolute number of nuclei is that the electron spin is coupled to, particularly since the splitting pattern is broadened out further away from the center. **Figure S1-3** shows the RMSD of the best fit for a chosen number of hydrogen nuclei. The best fit is obtained for 24 hydrogens which is consistent with coupling to the four β_1-hydrogens in all six porphyrin units (6x4=24). Only even numbers are shown as all odd numbers of hydrogens gave a notably worse fit.

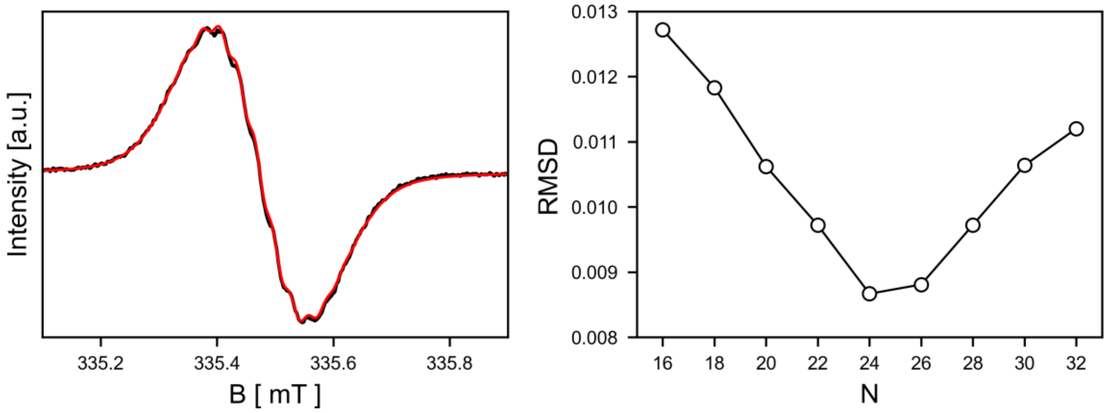

Figure S1-3. a) CW-EPR spectra of **[*c*-P6•T6]$^{•-}$** (black) recorded in THF with 10 mM [Bu_4N][PF_6] at room temperature at X-band frequencies and simulation with best fit (*N* = 24, red). b) RMSD of CW-EPR simulation while varying the number of nuclei.

1.4. Gaussian envelope fit

Spectral envelope widths were obtained by fitting a Gaussian derivative line shape to the experimental spectra and **Table S1-2.** Gaussian spectral envelope widths. lists these widths as they appear in Figure 3c of the main text.

Table S1-2. Gaussian spectral envelope widths.

Structure	ΔB_{pp} [mT]
***l*-P1$^{•-}$**	0.231 ± 4.38e-03
***l*-P2$^{•-}$**	0.166 ± 7.37e-05
***l*-P3$^{•-}$**	0.168 ± 1.99e-03
***l*-P4$^{•-}$**	0.144 ± 4.56e-04
***l*-P5$^{•-}$**	0.126 ± 1.11e-04
***l*-P6$^{•-}$**	0.117 ± 1.40e-04
***l*-P7$^{•-}$**	0.112 ± 2.25e-04
***l*-P8$^{•-}$**	0.107 ± 2.05e-04
***c*-P6$^{•-}$**	0.092 ± 2.17e-04
**[*c*-P6•T6]$^{•-}$**	0.085 ± 4.46e-05
***c*-P12$^{•-}$**	0.079 ± 1.87e-04

1.5. Voigt profile fit

For consistency with previous studies, the spectral widths in this manuscripts have been obtained by fitting a Gaussian derivative function. An alternative is to fit a Voigt profile, which take into account Gaussian as well as Lorentzian contributions to the line shapes and is defined as:

$$V(x;\sigma,\gamma) = \int_{-\infty}^{\infty} G(x';\sigma) * L(x-x';\gamma)\, dx'$$

$$G(x;\sigma) = \frac{1}{\sqrt{2\pi}\,\sigma} e^{-\frac{x^2}{2\sigma^2}}$$

$$L(x;\gamma) = \frac{\gamma}{\pi(\gamma^2 - x^2)}$$

where σ and γ are parameters specifying the widths of the Gaussian and Lorentzian components respectively. **Figure S1-4** b) shows the trend in the Gaussian component of the fitted Voigt profiles (**Figure S1-4** a), which behave similarly to the spectral envelope widths determined from only fitting a Gaussian derivative function. However, unlike in the purely Gaussian fit, the Gaussian components in the Voigt profile fits approach the Norris trend for longer oligomers. As is shown in the paper, the deviation from the Norris trend is likely to arise from incomplete delocalisation of spin density onto the terminal groups and it would therefore be expected that the deviations decrease in longer oligomers where the edges play a smaller role.

The larger linewidth obtained from a purely Gaussian fit accounts for additional line broadening from a larger Lorentzian line shape as can be determined from the trend in the parameter γ (**Figure S1-4** c). This is consistent with a slower rotational correlation time for larger oligomers due to their large size. The monomer and the cyclic structures don’t fit the same trend, which is likely due to their sufficiently different molecular geometry.

Note that the choice of spectral envelope that is fitted to the experimental CW-EPR spectra does not affect the overall interpretation of the data.

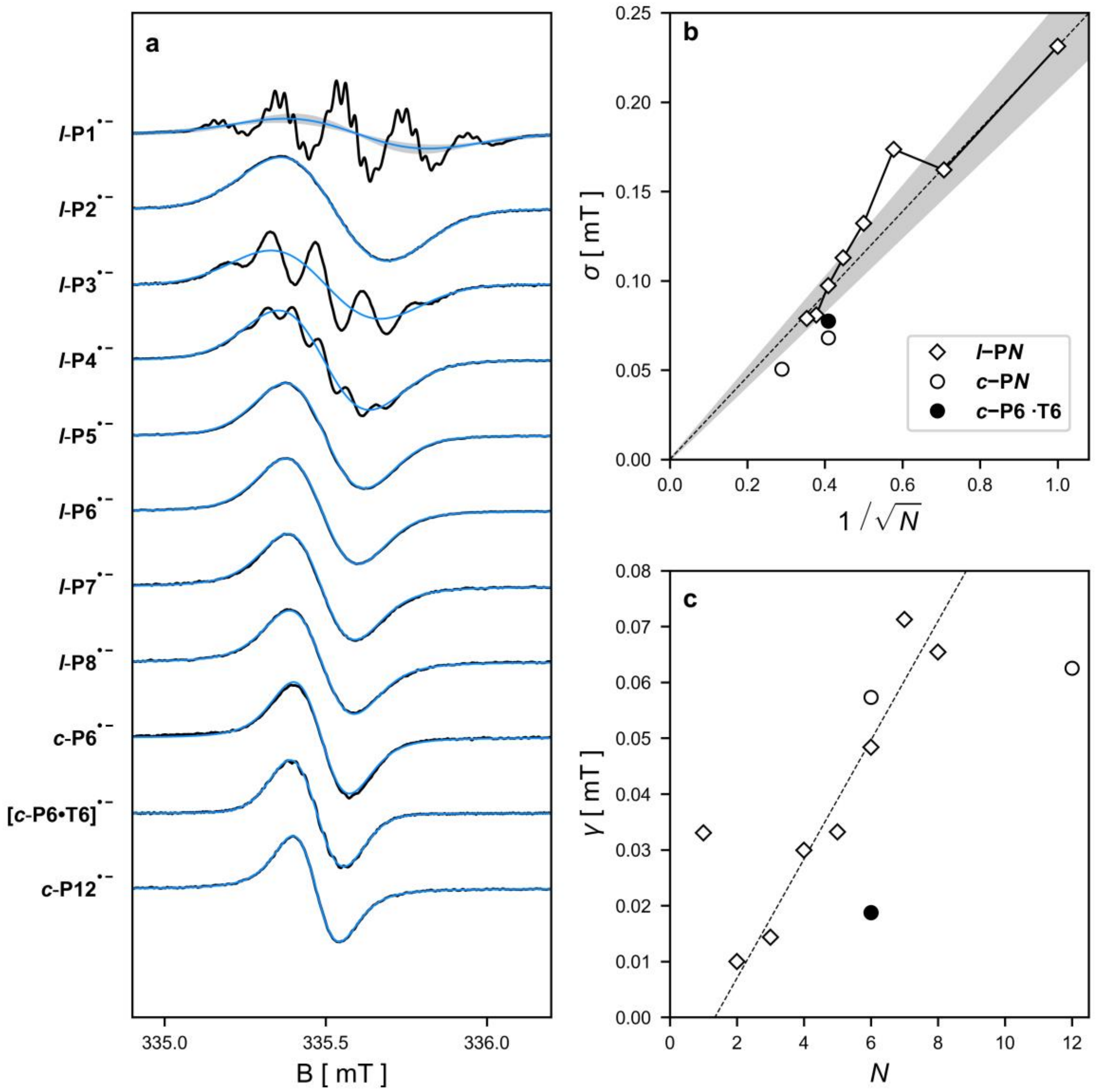

Figure S1-4. a) CW-EPR spectra recorded at 298 K in THF with 10 mM **TBAP** at X-band frequencies (black) and fitted Voigt profiles (blue). b) Trend in Gaussian component of Voigt profiles fitted to CW-EPR spectra. Dotted line shows expected prediction from Norris equation. Shaded area indicates 95% confidence interval and corresponds to the shaded area shown for ***l*-P1$^{•−}$** in (a). c) Trend in Lorentzian component of Voigt profiles fitted to CW-EPR spectra. Dotted trend line was fitted to values of ***l*-P*N*$^{•−}$** (*N* = 2-8), R^2= 0.938.

1.6. Low temperature CW-EPR

CW-EPR of the linear oligomers ***l*-P*N*$^{\bullet-}$** were recorded at 80 K and are shown in **Figure S1-5**. Interpretation of the linewidth is difficult, due to significant broadening arising from g-anisotropy. This prevents any conclusions in terms of the Norris relationship, since the main contribution to the line shape is no longer unresolved hyperfine coupling interactions.

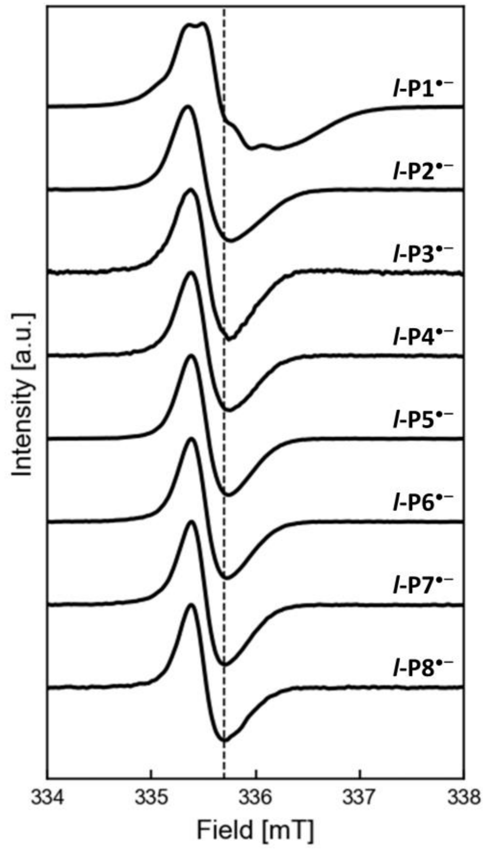

Figure S1-5. CW-EPR spectra recorded in THF with 10 mM [Bu_4N][PF_6] at 80 K at X-band frequencies. Dotted line is provided as a guide to the eye. Spectra are aligned to 9.4 GHz.

2. Pulsed EPR spectroscopy

2.1. Methodology

Samples for pulsed EPR measurements were prepared similarly as described in Section 1.1, but in deuterated toluene. The reducing agent solution and porphyrin sample were mixed directly inside a Q-band tube which was then sealed with vacuum grease before it was cycled out of the glove box and shock frozen. Due to the solvent of the reducing agent solution the overall solvent mixture was 1:10 THF:toluene(d8).

Pulsed EPR measurements were carried out at 80 K at Q-band frequencies using a Bruker E580 spectrometer. Hahn echo detected field sweeps were recorded with pulse lengths of $t_{\pi/2}$ = 16 ns and t_{π} = 32 ns and a delay of τ = 300 ns. Mims ENDOR was recorded at the field position with maximum intensity (corresponding to $g_{\perp}$) and at the shoulder of the spectrum (corresponding to $g_{\parallel}$) with the pulse sequence $\frac{\pi}{2} - \tau - \frac{\pi}{2} - T - \frac{\pi}{2} - \tau - echo$ with $t_{\pi/2}$ = 16 ns and a 12 µs radiofrequency pulse during the delay *T*. The power of the radiofrequency pulse was adjusted to obtain a π pulse for a ^{1}H nucleus. Radiofrequencies were varied between 5 MHz below and 5 MHz above the hydrogen Larmor frequency and measured in stochastic order to avoid rf-heating of the sample. The delay τ was varied between 150 and 330 ns in 60 ns increments and spectra were added together to account for blind-spots that arise in Mims ENDOR measurements.

2.2. Echo detected field sweep spectra

The echo detected field sweep spectra of the anions of porphyrin oligomers are shown in **Figure S2-1**. The spectrum of ***l*-P1$^{•−}$** exhibits a much larger g-anisotropy then the longer oligomers, which all have similar spectra from ***l*-P2$^{•−}$** onwards. The cyclic structures display less anisotropic spectra and in particular the rigid and more symmetric **[*c*-P6•T6]$^{•−}$** is nearly isotropic. The free rings ***c*-P6$^{•−}$** and ***c*-P12$^{•−}$** in comparison lie between the linear structures and the highly symmetric **[*c*-P6•T6]$^{•−}$**, likely due to deformation of the ring and localisation of the spin density on some part of the rings giving a similar environment to that of a free porphyrin chain.

Simulations of the spectra reveal that the spectral shapes cannot be obtained from basic simulations, because the shoulder arising from $g_{\parallel}$ has a lower in intensity than one might expect. This is due to different relaxation rates for different orientations (see **Figure S2-2**) and therefore different echo intensities along the field axis. This was accounted for in the simulations shown in **Figure S2-1** by simulating all orientations separately and subsequently weighing them by a sigmoid function that decreases from $g_{\perp}$ towards $g_{\parallel}$.

The anions of the linear oligomers are noticeably more anisotropic than the corresponding cationic species, with the difference in $g_{\perp}$ and $g_{\parallel}$ being roughly four to five times larger.

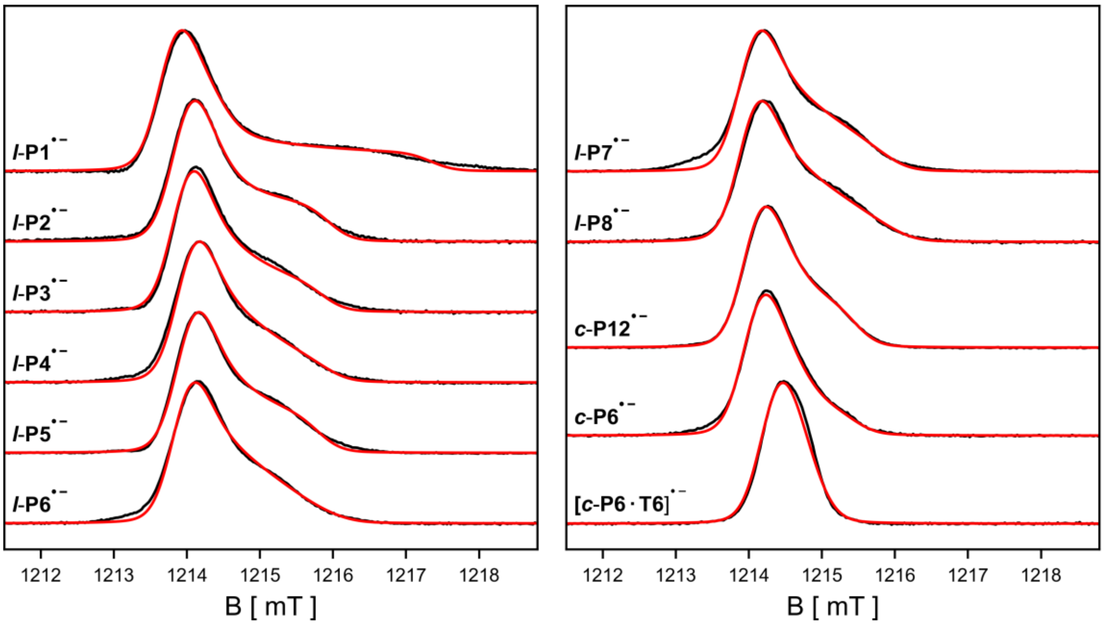

Figure S2-1. Echo detected field sweep spectra (black) recorded at 80 K at Q-band frequencies and simulations (red).

Table S2-1. *g*-values obtained from fits to echo-detected field sweeps corrected with g_{iso} obtained from CW-EPR.

Structure	$g_{\perp}$	$g_{\parallel}$	Δg
***l*-P1$^{\bullet-}$**	2.0032	1.9973	0.0059
***l*-P2$^{\bullet-}$**	2.0028	1.9995	0.0033
***l*-P3$^{\bullet-}$**	2.0029	1.9997	0.0032
***l*-P4$^{\bullet-}$**	2.0029	1.9996	0.0033
***l*-P5$^{\bullet-}$**	2.0028	1.9998	0.0030
***l*-P6$^{\bullet-}$**	2.0032	1.9994	0.0038
***l*-P7$^{\bullet-}$**	2.0030	1.9997	0.0033
***l*-P8$^{\bullet-}$**	2.0031	1.9996	0.0035
***c*-P6$^{\bullet-}$**	2.0028	2.0005	0.0023
**[*c*-P6•T6]$^{\bullet-}$**	2.0023	2.0013	0.0010
***c*-P12$^{\bullet-}$**	2.0027	2.0004	0.0023

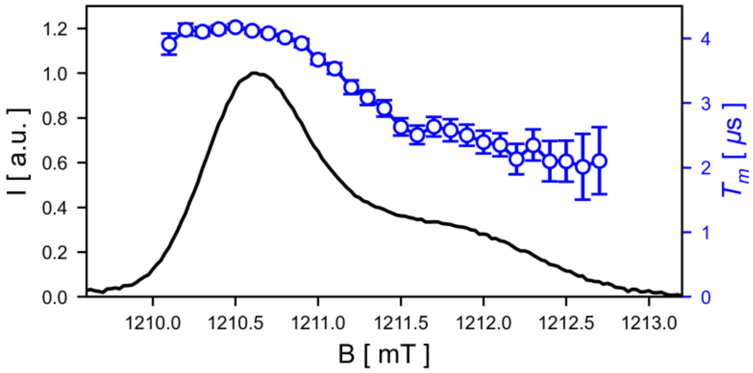

Figure S2-2. Echo detected field sweep spectrum of ***l*-P2$^{\bullet-}$** recorded at 80 K at Q-band frequencies (black) and field dependent T_m relaxation times (blue).

2.3. ENDOR spectra at $g_\perp$ and $g_\parallel$

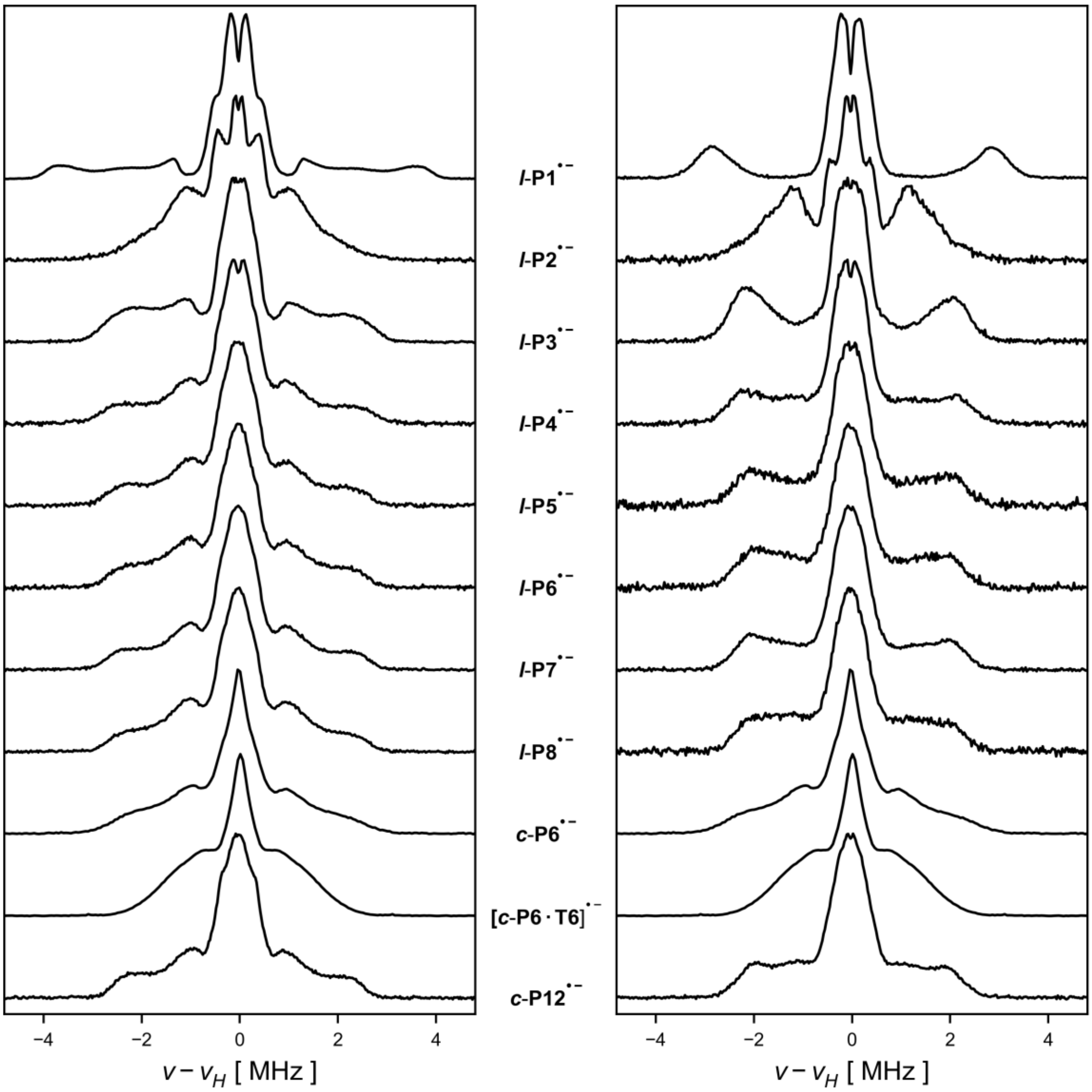

Figure S2-3. ENDOR spectra recorded at field positions corresponding to $g_\perp$ (left) and $g_\parallel$ (right). Note that the same spectrum is plotted twice for ***c*-P6•−** and **[*c*-P6•T6]•−** due to the much smaller anisotropy in these systems.

2.4. ENDOR derivatives used to estimate spectral widths

Extracting the width of the ENDOR spectra is not trivial due to their complex shapes. Instead of picking the full width at half maximum we picked the first turning points of the spectra i.e. the maxima/minima of the derivatives of the spectra. The experimental spectrum was smoothed prior to differentiation to reduce noise levels. This was done using a Savitzky-Golay filter to minimize distortions of the spectral shape. **Figure S2-4** shows that the spectral shape is retained very well except for some minor changes in the central region which are not important for determining the width of the spectrum.

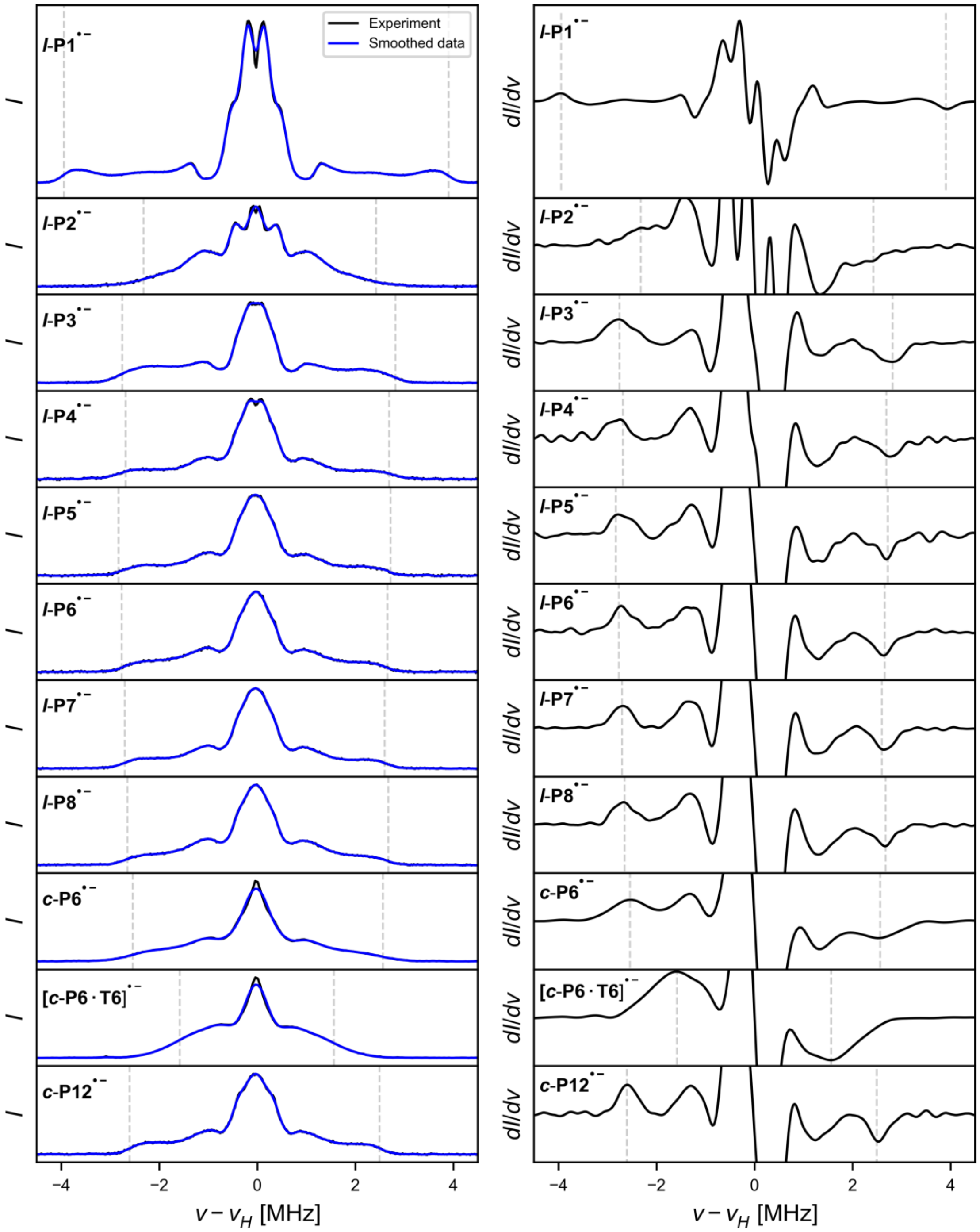

Figure S2-4. Left: Spectra (black) and spectra smoothed using a Savitzky-Golay filter (blue). Right: Derivative of smoothed spectrum. Dotted lines indicate turning points.

The resulting spectral widths are listed in **Table S2-2** below.

Table S2-2. ENDOR spectral widths

	**ENDOR width [MHz]**
***l*-P1$^{\bullet-}$**	7.83
***l*-P2$^{\bullet-}$**	4.50
***l*-P3$^{\bullet-}$**	5.62
***l*-P4$^{\bullet-}$**	5.38
***l*-P5$^{\bullet-}$**	5.53
***l*-P6$^{\bullet-}$**	5.45
***l*-P7$^{\bullet-}$**	5.17
***l*-P8$^{\bullet-}$**	5.35
***c*-P6$^{\bullet-}$**	5.00
**[*c*-P6•T6]$^{\bullet-}$**	3.10
***c*-P12$^{\bullet-}$**	5.23

3. Modelling and Simulation

3.1. Methodology

Structures of the porphyrin oligomers were optimized at the PBE0+D3(BJ)/def2-SVP(H, C, N)/def2-TZVP(Zn) level of theory using Gaussian 16 and a tight convergence threshold. Frequency calculations were attempted to confirm minima but were not successful due to the large system size. All calculations were done with phenyl sidechains instead of the bulky 3,5-bis(trihexylsilyl)phenyl side chains to speed up computation times. Calculations of EPR parameters on the optimized structures were subsequently carried out in ORCA 5.0. The range-separated functional lc-ωPBE with range-separation parameter ω between 0.075 and 0.225 was screened while using the EPR-II basis set for hydrogen atoms, def2-SVP on carbon and nitrogen atoms and def2- TZVP on Zn atoms. No significant change in hyperfine coupling values was observed when using the EPR-II basis set also on C and N (RMSD < 0.1 MHz) and therefore def2-SVP was chosen for those atoms to improve computational efficiency.

Ab initio simulations were carried out using ORCA 5.0 in conjunction with the extended tight binding (xtb) software package as a driver to calculate forces. Simulations were performed with implicit THF solvent at 298 K over 40-50 ps using a time step of 0.5 fs. Simulations were checked for correct conservation of conserved quantities and correct sampling of the target temperature. Subsequently, DFT single point calculations including calculations of hyperfine couplings were performed on frames selected at 0.5 ps intervals using the lc-ωPBE functional with a range-separation parameters ω of 0.2 as this value gave good agreement with low temperature ENDOR experiments.

Simulations of EPR spectra were done in MATLAB using the EasySpin toolbox. CW-EPR spectra recorded at room temperature were simulated using the garlic function. Only hyperfine couplings of β protons on the porphyrins were used in the simulations as the magnitude of the hyperfine couplings on the side chains are negligible. In addition, there would be a too large number of hyperfine couplings for the larger systems and so exclusion of them for the shorter systems gives a more consistent comparison between those. A_{iso} values for single point calculations are directly obtained from the DFT output files. Hyperfine couplings corresponding to equivalent environments due to symmetry were averaged and their recurrence accordingly handled in Easyspin. A_{iso} values corresponding to molecular dynamics simulations were obtained by first averaging the hyperfine coupling over the course of the simulation for each nucleus respectively.

^{1}H Mims ENDOR spectra were simulated using the saffron function. ENDOR spectra for different hydrogen nuclei were simulated separately and summed up subsequently. Simulations used the fitted values for g obtained in section 2.2 and the experimental values for excitation width and τ. Hyperfine coupling tensors (Magnitude and orientation) are directly obtained from the DFT output files. The ENDOR linewidth was adjusted to optimize the fit between experiment and simulations and ranged between 0.4 and 0.6.

3.2. Simulations

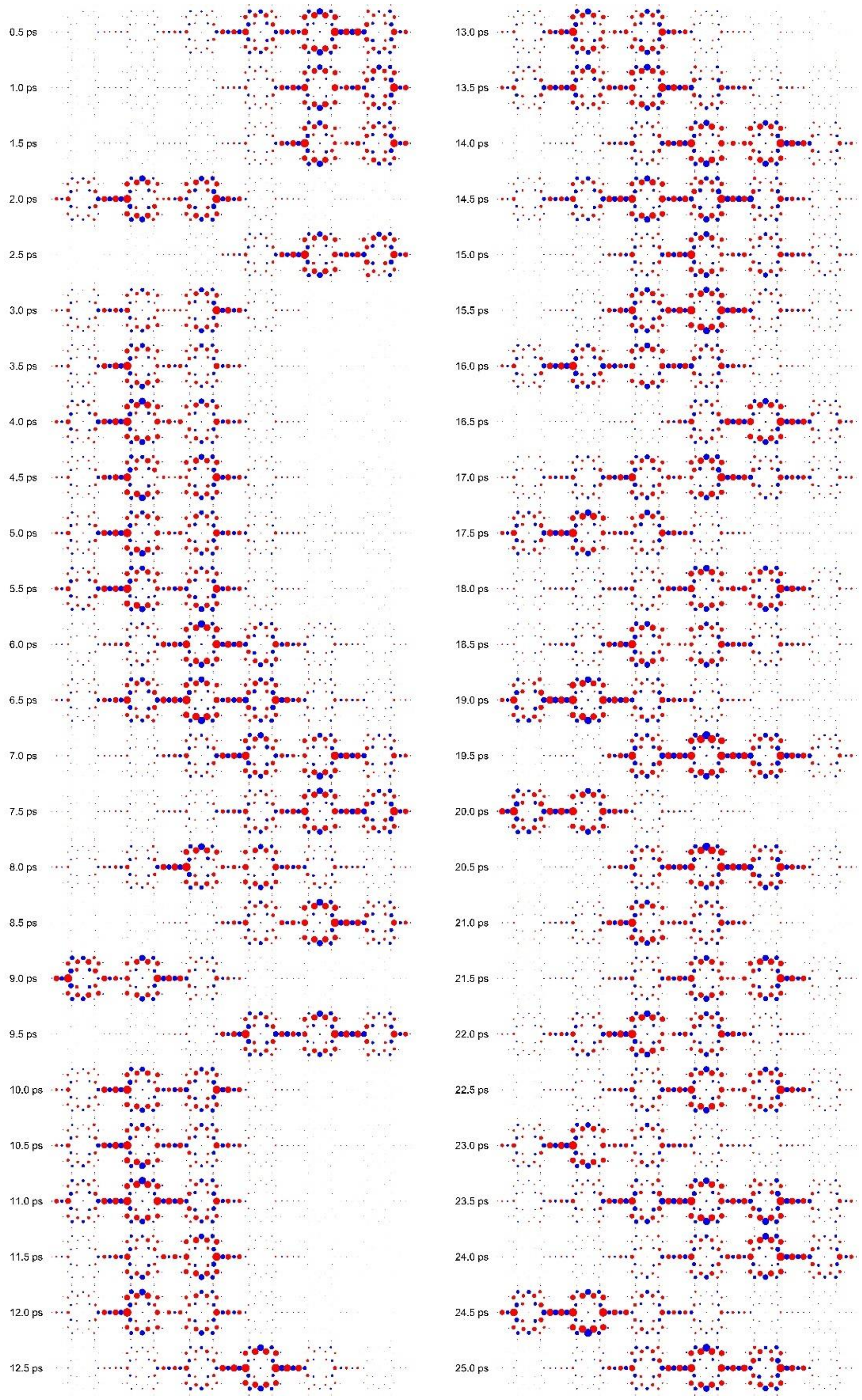

Figure S3-1. Spin density plots of structures sampled during MD simulation of ***l*-P6**$^{\bullet-}$ superimposed on optimized structure for visualisation purposes. Single point calculations were done with lc-ωPBE (ω=0.2) /def2SVP(H,C,N)+def2TZVP(Zn).

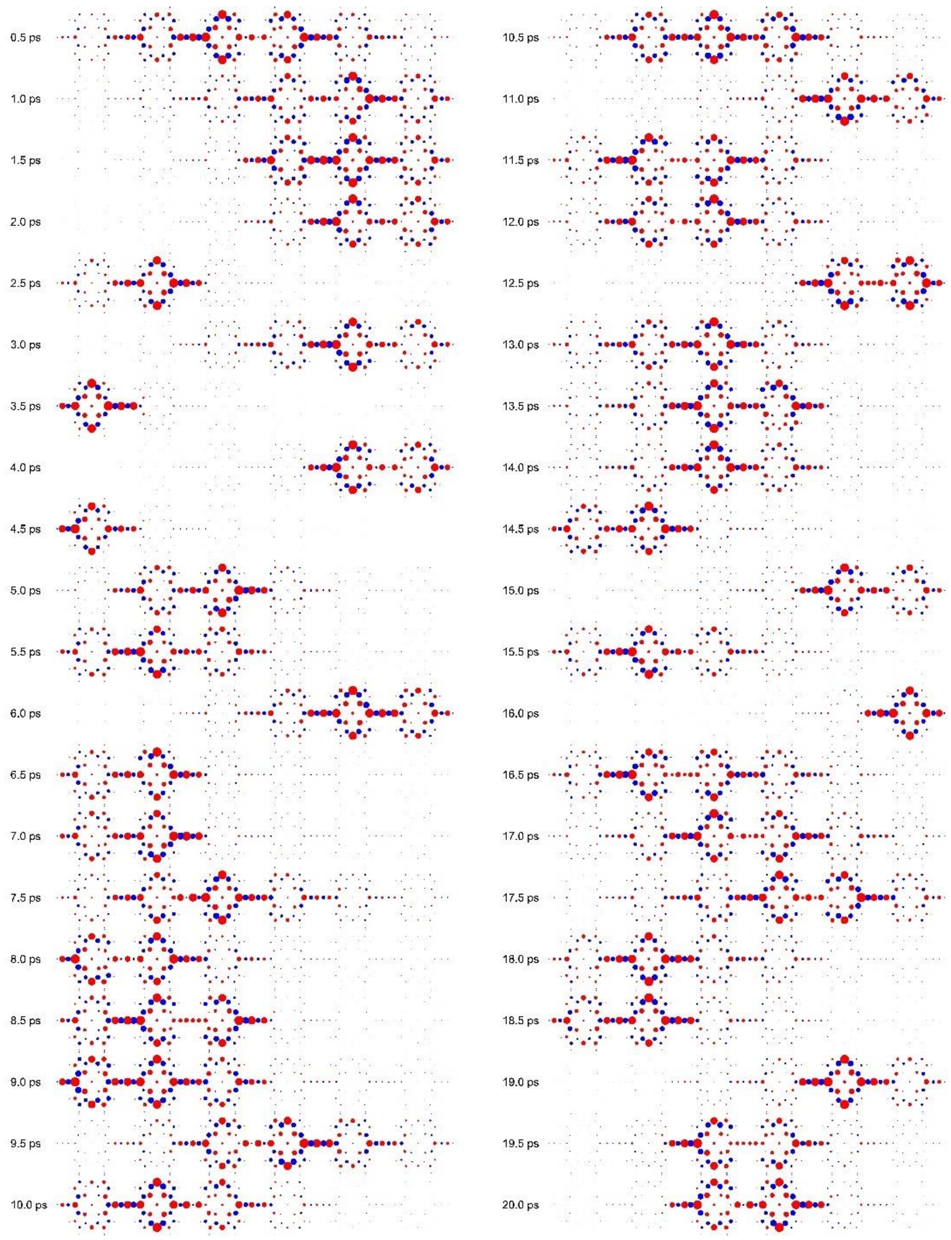

Figure S3-2. Spin density plots of structures sampled during MD simulation of ***l*-P6**$^{\bullet+}$ superimposed on optimized structure for visualisation purposes. Single point calculations were done with lc-ωPBE (ω=0.2) /def2SVP(H,C,N)+def2TZVP(Zn).

3.3. CW-EPR

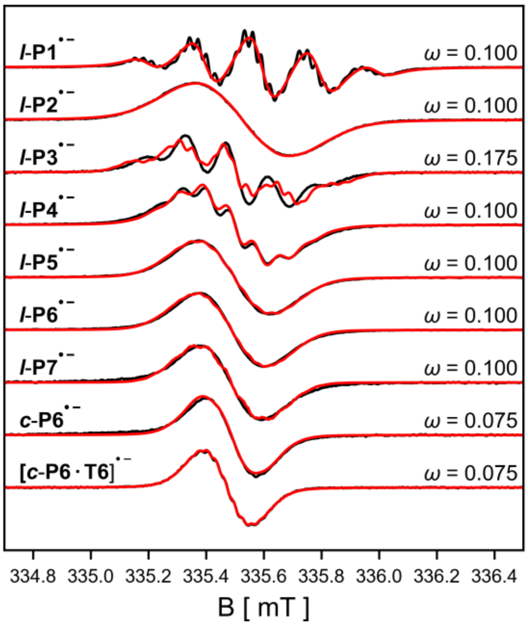

Figure S3-3. CW-EPR spectra of radical anions recorded at room temperature in THF with 10 mM $[Bu_4N][PF_6]$ at X-band frequencies (black) and simulations using hyperfine couplings obtained from DFT calculations with the lc-ωPBE functional (red). The value of ω that gave the best fit with agreement is given on the right.

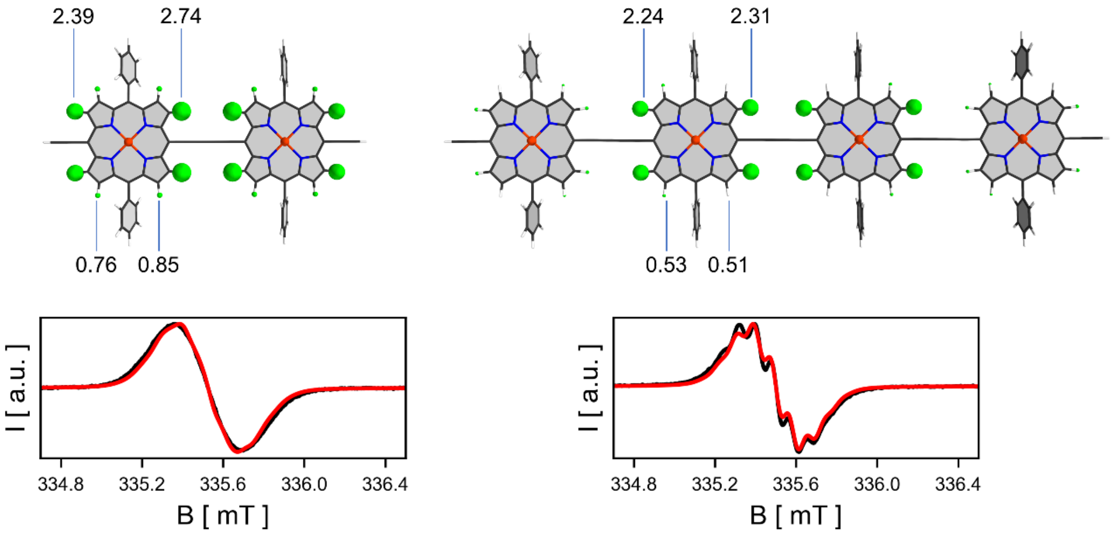

Figure S3-4. Comparison of the 16 largest hyperfine couplings in ***l*-P2**$^{\bullet-}$ and ***l*-P4**$^{\bullet-}$ located on the two central porphyrins (radius of green spheres give isotropic hyperfine coupling) obtained from DFT calculations with the lc-ωPBE functional (ω=0.1 gives best fit for both systems). Hyperfine coupling constants are shown in MHz. Below, the corresponding CW-EPR spectra recorded at room temperature in THF with 10 mM $[Bu_4N][PF_6]$ at X-band frequencies (black) and simulations (red) are shown. Both systems use the same Lorentzian line width of 0.04 mT and a Gaussian line width of 0.03 mT.

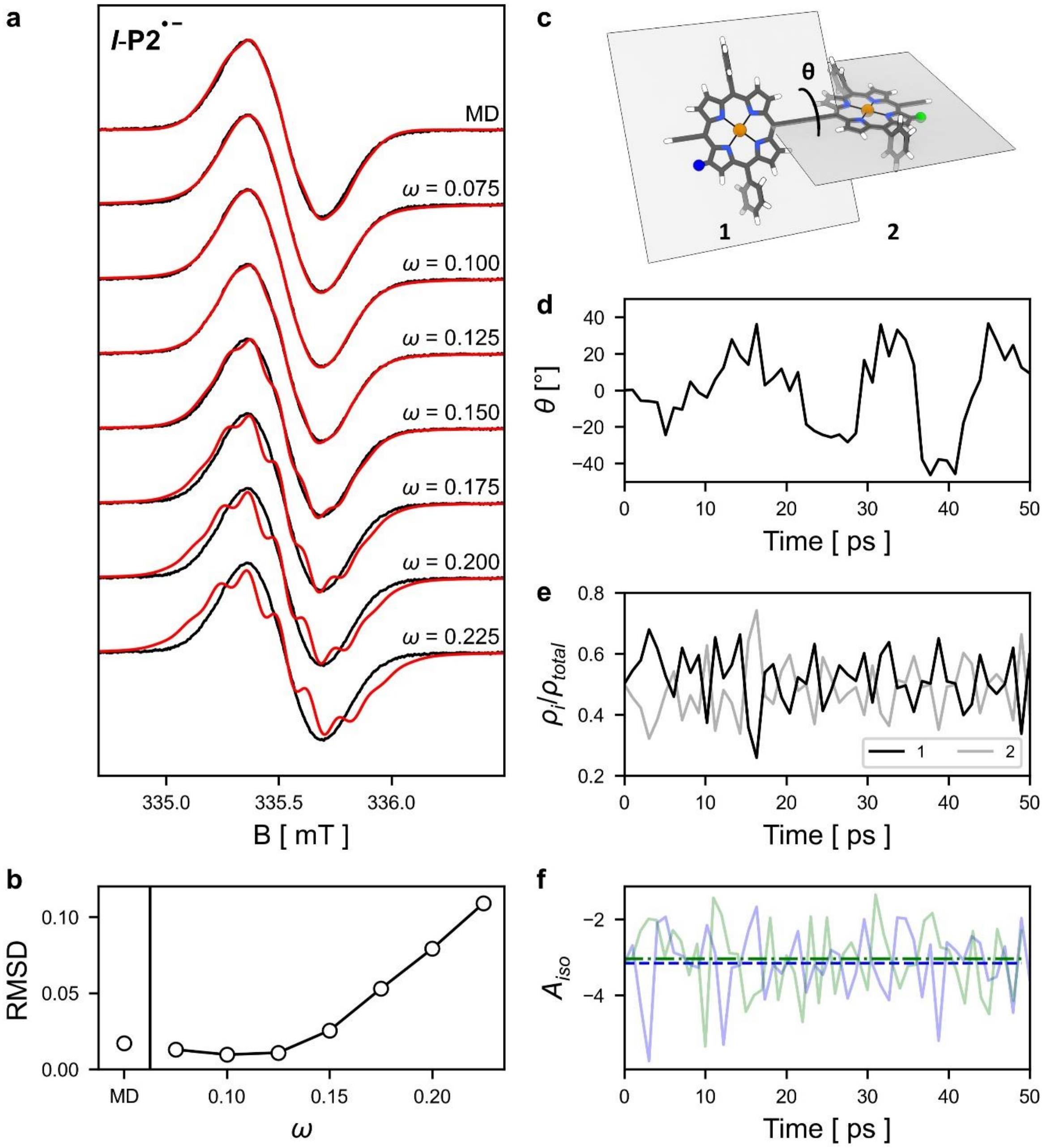

Figure S3-5. (a) CW-EPR spectra of ***l*-P2•−** recorded at room temperature in THF with 10 mM [Bu_4N][PF_6] at X-band frequencies (black) and simulations using hyperfine couplings obtained from either DFT calculations with the lc-ωPBE functional with different ω parameters or from AIMD calculations(red). (b) RMSDs between simulations and experimental spectra. (c) Structure of ***l*-P2•−** illustrating dihedral angle θ. (d) Evolution of dihedral angle during the molecular dynamics simulation. (e) Relative spin densities on each porphyrin during the molecular dynamics simulation. (f) Evolution of selected hyperfine couplings corresponding to hydrogens that are related by symmetry as indicated in c). Faint lines show hyperfine couplings for a given frame while dashed lines show time average.

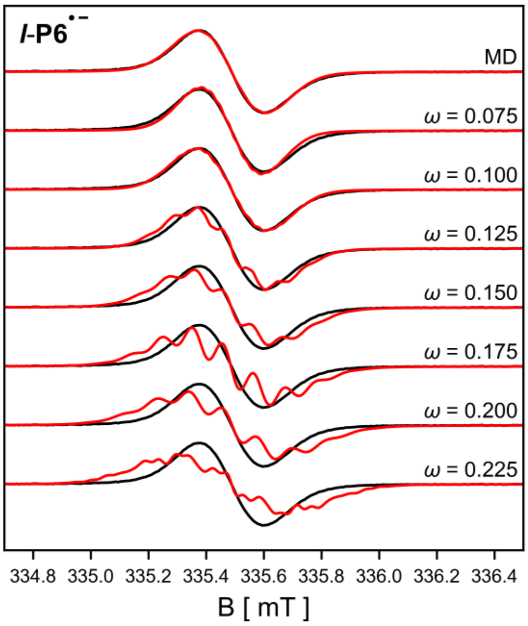

Figure S3-6. CW-EPR spectra of ***l*-P6$^{•-}$** recorded at room temperature in THF with 10 mM $[Bu_4N][PF_6]$ at X-band frequencies (black) and simulations using hyperfine couplings obtained from either DFT calculations with the lc-ωPBE functional with different ω parameters or from AIMD calculations(red).

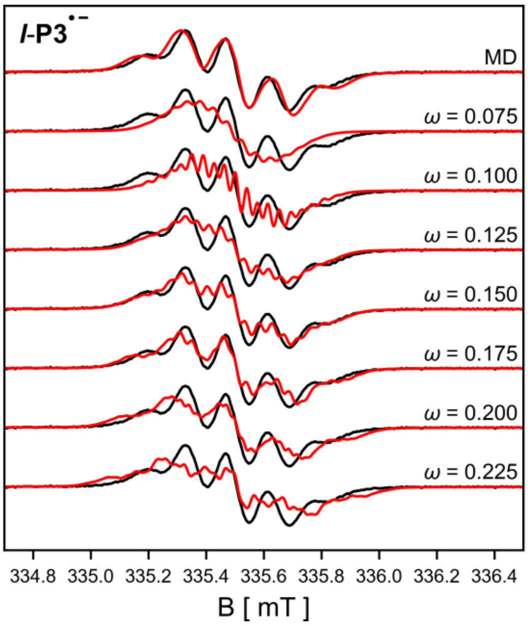

Figure S3-7. CW-EPR spectra of ***l*-P3$^{•-}$** recorded at room temperature in THF with 10 mM $[Bu_4N][PF_6]$ at X-band frequencies (black) and simulations using hyperfine couplings obtained from either DFT calculations with the lc-ωPBE functional with different ω parameters or from AIMD calculations(red).

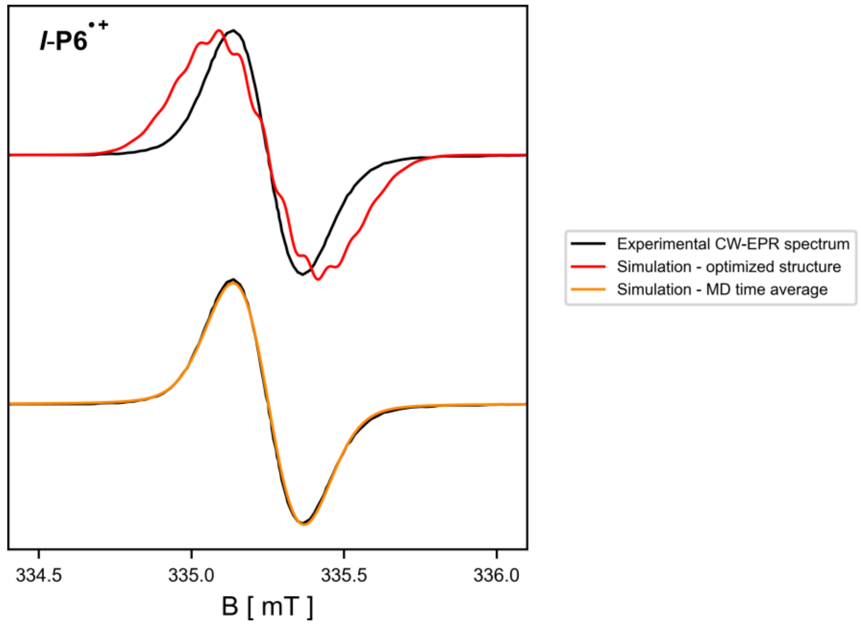

Figure S3-8. Room temperature CW-EPR spectrum of ***l*-P6**$^{•+}$ reproduced from reference [X] and simulations using hyperfine couplings obtained from either DFT calculations (red) with the lc-ωPBE functional (ω = 0.2) or from MD calculations (orange).

3.4. ENDOR

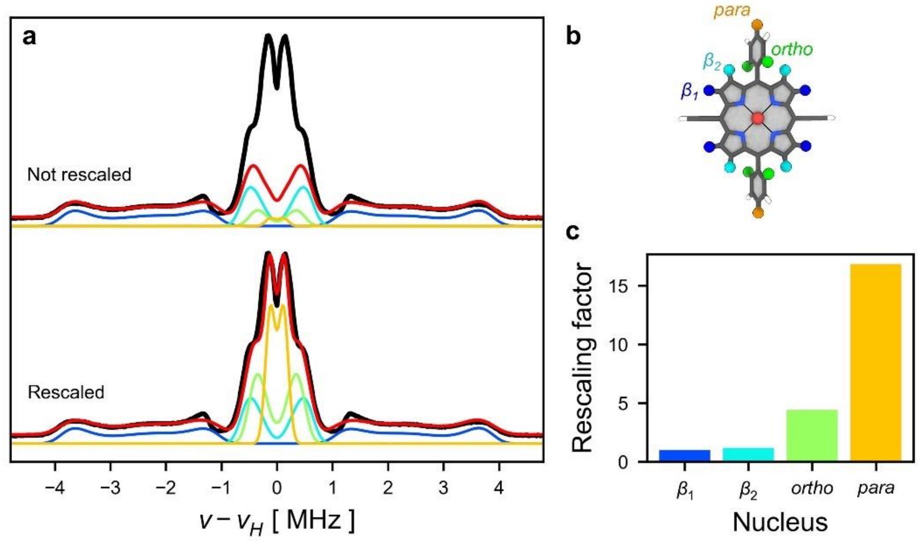

Figure S3-9. (a) ENDOR spectrum of ***l*-P1•−** recorded at 80 K at Q-band frequencies at field position corresponding to $g_{\perp}$(black) and simulations (red) where intensities have (bottom) or have not (top) been rescaled. Contributions from individual nuclei are offset for clarity. (b) Structure of ***l*-P1•−** with hydrogen nuclei contributing to the spectrum highlighted. (c) Relative rescaling factors for best fit simulation shown in (a) bottom.

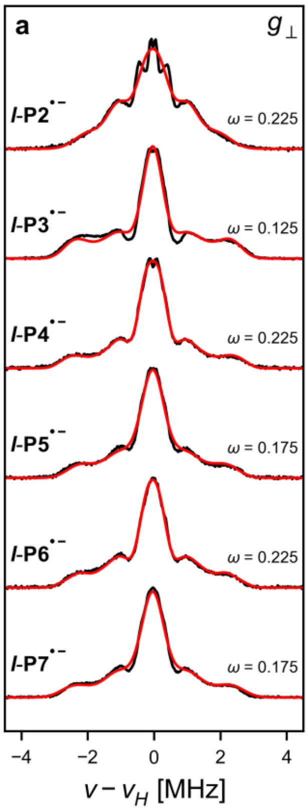

Figure S3-10. [1]H Mims ENDOR spectra of ***l*-P*N*•−** recorded at 80 K at Q-band frequencies (black) and simulations (red) using hyperfine couplings obtained from DFT calculations with the lc-ωPBE functional. Intensities were freely rescaled to fit to spectrum. The value of ω that gave the best fit is given on the right.

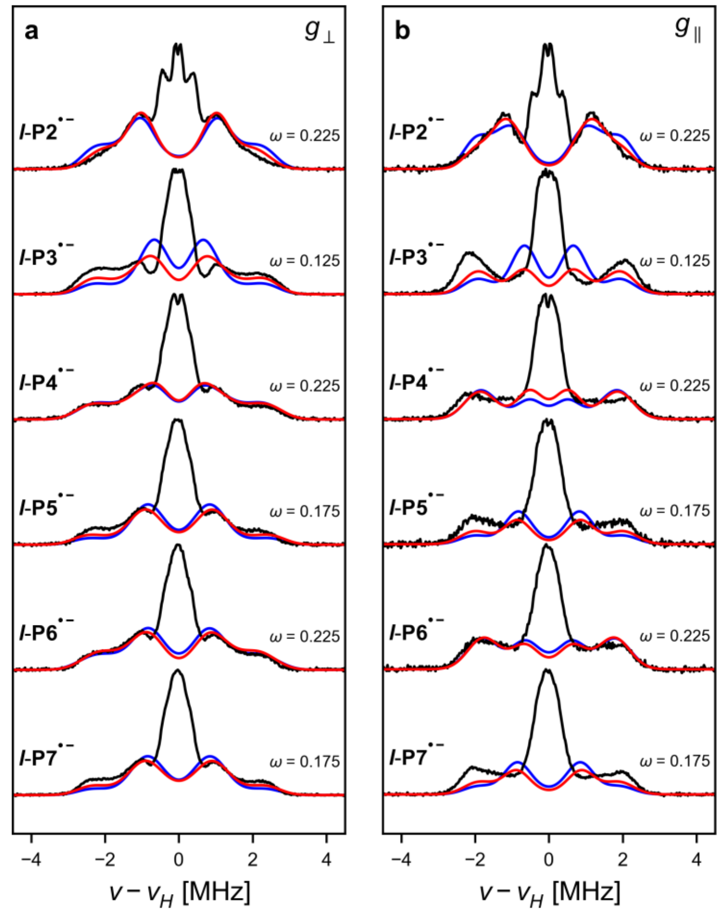

Figure S3-11. [1]H Mims ENDOR spectra of ***l*-P*N*•−** recorded at 80 K at Q-band frequencies (black) and simulations using β-H hyperfine couplings obtained from DFT calculations with the lc-ωPBE functional. Simulations with no rescaling are shown in blue and simulations with a 30% rescaling range in red. The value of ω that gave the best fit with agreement is given on the right.

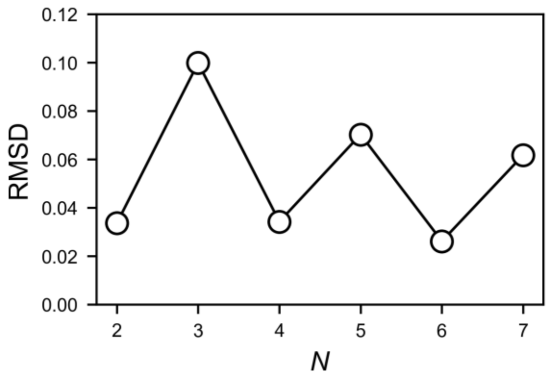

Figure S3-12. Root mean square deviation (RMSD) between experimental ^{1}H ENDOR spectra and simulations for linear oligomers ***l*-P2•−** to ***l*-P7•−**.

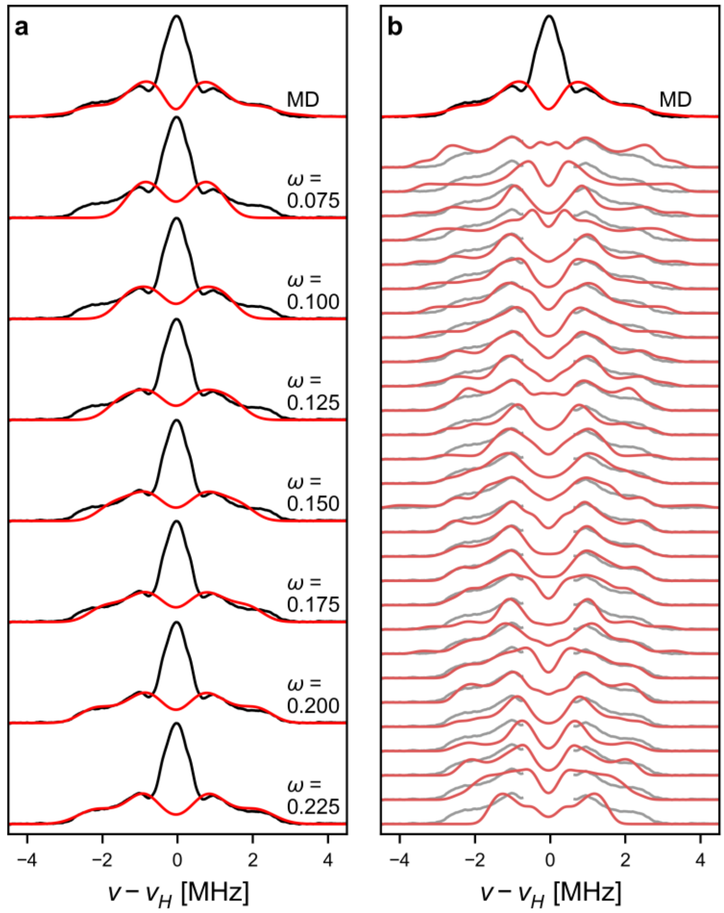

Figure S3-13. (a) [1]H Mims ENDOR spectrum of ***l*-P6**$^{\bullet-}$ recorded at 80 K at Q-band frequencies (black) and simulations (red) using β-H hyperfine couplings obtained from DFT calculations with the lc-ωPBE functional with different values of ω as indicated on the right. Spectrum for AIMD simulation is the average spectrum of all single point calculations on the structures sampled by MD. (b) Average spectrum of structures sampled from MD simulation (top) and individual spectra (arbitrarily sorted by largest hyperfine coupling for better visualisation).

3.5. Quantifying Delocalization

The expression in equation (1) is used to quantify the delocalization length N_{deloc} based on the spin densities on each porphyrin. It is defined as the sum of the ratios of actual spin densities ρ_i and hypothetical spin densities for full and uniform delocalization (1/*N*). If the spin density on a given porphyrin is larger than it would be for full uniform delocalization, it only adds 1 to the sum. This definition ensures that N_{deloc} is equal to *N* (number of units) for full and uniform delocalization and equal to 1 for full localisation.

$$N_{deloc} = \sum_{i=1}^{N} \min\left(\frac{\rho_i}{1/N}, 1\right) \tag{1}$$

The resulting delocalization lengths based on the DFT calculations shown above (using ω = 0.2) are plotted in **Figure S3-14** for the oligomers up to *N* = 6. Only the dimer shows full delocalization after which the delocalization length N_{deloc} deviates from *N* and converges towards ***l*-P6$^{\bullet-}$** at N_{deloc} = 3.33. **Figure S3-15** shows how the delocalization length varies for the structures sampled during the MD simulation of ***l*-P6$^{\bullet-}$**. While there is a slight decrease (average N_{deloc} = 2.93), the delocalization length is not significantly affected by the structural disorder. The similar extent of delocalization in optimized and structurally distorted structures shows that the delocalization is not limited by conformational disorder.

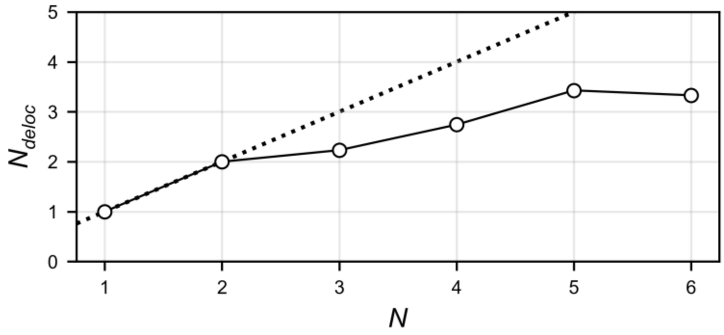

Figure S3-14. Delocalization length as defined in equation (1) for ***l*-P*N*$^{\bullet-}$** calculated from spin densities computed with lc-ωPBE (ω=0.2).

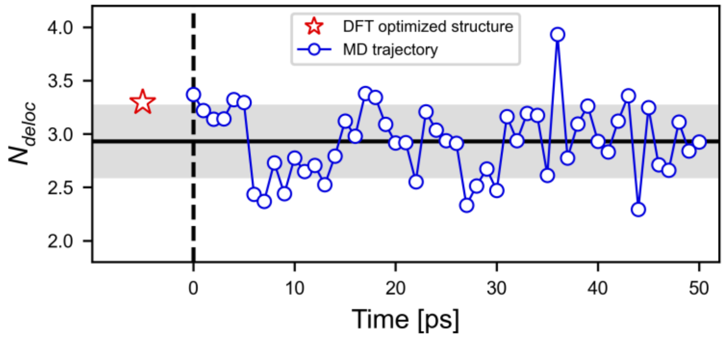

Figure S3-15. Delocalization length as defined in equation (1) for ***l*-P6$^{\bullet-}$** during MD simulation trajectory and for DFT optimized structure.

3.6. Electronic structure of the neutral molecules

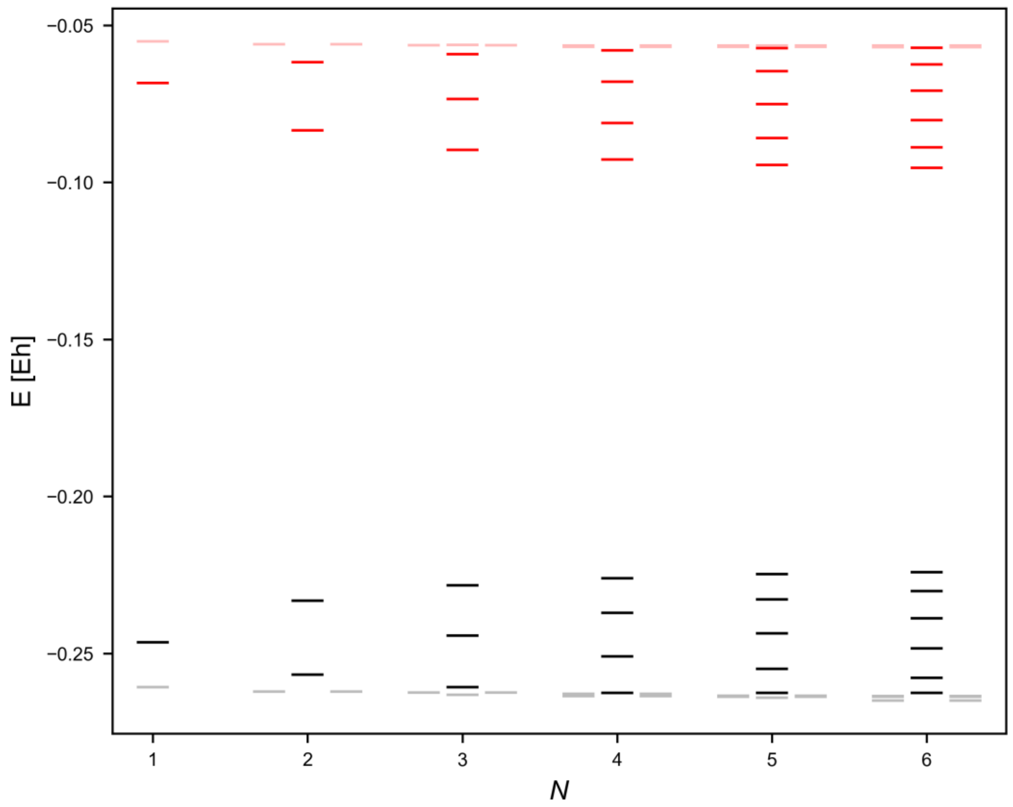

Figure S3-16. Energy of frontier orbitals for ***l*-P*N*•−** (*N* = 1-6) calculated with lc-ωPBE(ω=0.2)/ def2SVP(H,C,N)+def2TZVP(Zn). Occupied orbitals are shown in black and unoccupied orbitals in red. The first *N* orbitals are plotted as solid lines and the next *N* orbitals as faint lines.

4. Radical anions of partially deuterated porphyrins

4.1. Synthesis

4.1.1. Synthesis of *d*-H_2P1$_{THS}$

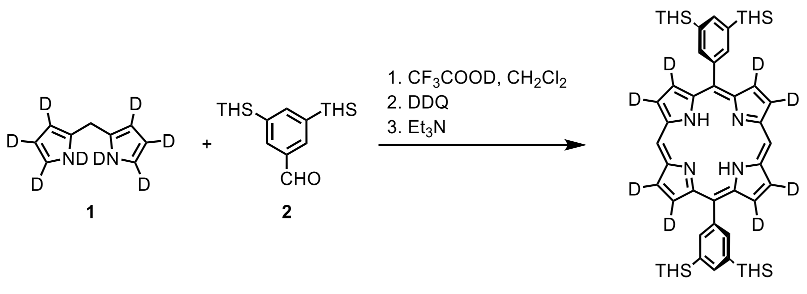

***d*-H_2P1$_{THS}$**

Bis(1*H*-pyrrol-2-yl)methane-*d* **1** [5] and bis(trihexylsilyl)benzaldehyde **2** [6] were synthesized as reported previously. A mixture of bis(1*H*-pyrrol-2-yl)methane-*d* **1** (0.23 g, 1.5 mmol, 1 equiv.), 3,5-bis(trihexylsilyl)benzaldehyde **2** (1.0 g, 1.5 mmol, 1 equiv.) in dry CH_2Cl_2 (400 mL) was degassed by bubbling with argon for 15 min. After that, trifluoroacetic acid-d (0.21 mL, 2.7 mmol, 1.8 equiv.) was added and the reaction mixture was stirred at 25 °C in the dark for 3 h. 2,3-dichloro-5,6-dicyano-1,4-benzoquinone (DDQ, 0.51 g, 2.2 mmol, 1.5 equiv.) was then added and the reaction mixture stirring continued for 20 min. Triethylamine (Et_3N, 1.5 mL) was added to quench the reaction. The resulting mixture was concentrated, and separated by flash column chromatography (SiO_2) using pentane/CH_2Cl_2 (9:1) as eluent to give the desired product ***d*-H_2P1$_{THS}$** (0.78 g, 65%).

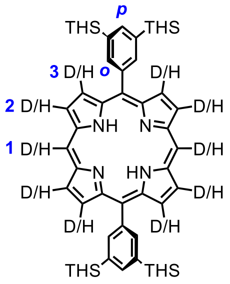

^{1}H NMR (600 MHz, $CDCl_3$, 298 K) δ 10.35 (s, 2H, **H_1**, 41% D), 9.41 (s, 4H, **H_2**, 83% D), 9.10 (s, 4H, **H_3**, 83% D), 8.39 (d, *J* = 1.2 Hz, 4H, **H_o**), 8.04 (t, *J* = 1.2 Hz, 2H, **H_p**), 1.59 – 0.85 (m, 156H, **H_{THS}**), –3.01 (s, 2H, **H_{NH}**).

MALDI-ToF: m/z = 1600.094 ($C_{104}H_{166}D_8N_4Si_4$, $M^{+\bullet}$ requires 1600.335).

UV–vis–NIR ($CDCl_3$, 298 K) λ_{max} / nm (ε / mM^{-1} cm^{-1}): 411 (425), 506 (17.8), 541 (7.37), 578 (5.67), 633 (2.69).

4.1.2. Synthesis of ***d*-ZnP1$_{THS}$**

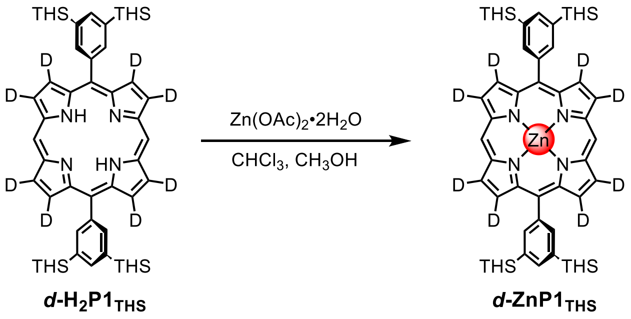

To a solution of ***d*-H$_2$P1$_{THS}$** (0.75 g, 0.47 mmol, 1.0 equiv.) in $CHCl_3$ (40 mL), a solution of $Zn(OAc)_2$· $2H_2O$ (0.51 g, 2.3 mmol, 5.0 equiv.) in CH_3OH (10 mL) was added. The reaction mixture was stirred at 40 °C for 15 min. After reaction, the resulting mixture was separated by flash column chromatography (SiO_2) using pentane/CH_2Cl_2 (9:1) as eluent to give the desired product ***d*-ZnP1$_{THS}$** (0.78 g, >99%).

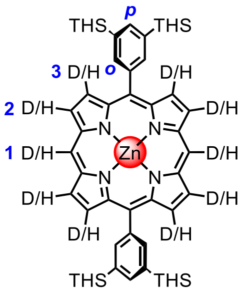

***d*-ZnP1$_{THS}$**

^{1}H NMR (600 MHz, $CDCl_3$, 298 K) δ 10.38 (s, 2H, **H$_1$**, 41% D), 9.47 (s, 4H, **H$_2$**, 83% D), 9.17 (s, 4H, **H$_3$**, 83% D), 8.38 (d, J = 1.2 Hz, 4H, **H$_o$**), 8.04 (t, J = 1.2 Hz, 2H, **H$_o$**), 1.64 – 0.71 (m, 156H, **H$_{THS}$**).

MALDI-ToF: m/z = 1663.015 ($C_{104}H_{164}D_8N_4Si_4Zn$, $M^{+\bullet}$ requires 1663.247).

UV–vis–NIR ($CDCl_3$, 298 K) λ_{max} / nm (ε / mM^{-1} cm^{-1}): 411 (438), 536 (23.0), 571 (2.78).

4.1.3. Synthesis of ***d*-ZnP1$_{THS}$-Br$_2$**

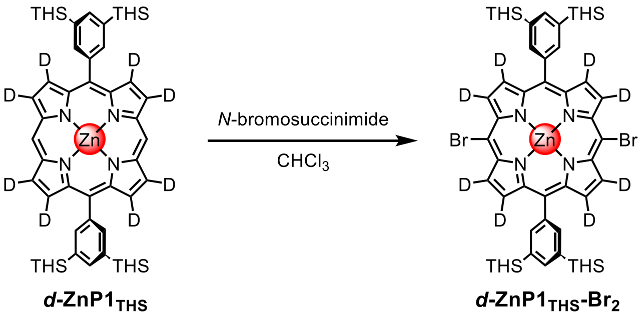

To a solution of ***d*-ZnP1$_{THS}$** (0.75 g, 0.45 mmol, 1.0 equiv.) in $CHCl_3$ (40 mL), a solution of *N*-bromosuccinimide (NBS, 0.17 g, 0.95 mmol, 2.1 equiv.) in $CHCl_3$ (10 mL) was added. The reaction mixture was stirred at 0 °C for 15 min. After that, the reaction was quenched with acetone (0.2 mL), and the resulting mixture was separated by flash column chromatography (SiO_2) using pentane/CH_2Cl_2 (9:1) as eluent to give the desired product ***d*-ZnP1$_{THS}$-Br$_2$** (0.81 g, 99%).

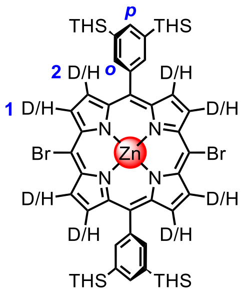

***d*-ZnP1$_{THS}$-Br$_2$**

^{1}H NMR (600 MHz, $CDCl_3$, 298 K) δ 9.75 (s, 4H, **H$_1$**, 83% D), 8.96 (s, 4H, **H$_2$**, 83% D), 8.26 (s, 4H, **H$_o$**), 8.03 (s, 2H, **H$_p$**), 1.91 – 0.62 (m, 156H, **H$_{THS}$**).

MALDI-ToF: m/z = 1820.950 ($C_{104}H_{162}D_8Br_2N_4Si_4Zn$, $M^{+\bullet}$ requires 1821.066).

UV–vis–NIR ($CDCl_3$, 298 K) λ_{max} / nm (ε / μM^{-1} cm^{-1}): 428 (447), 557 (26.3), 596 (9.54).

4.1.4. Synthesis of *d-ZnP1$_{THS}$*-CPDIPS$_2$

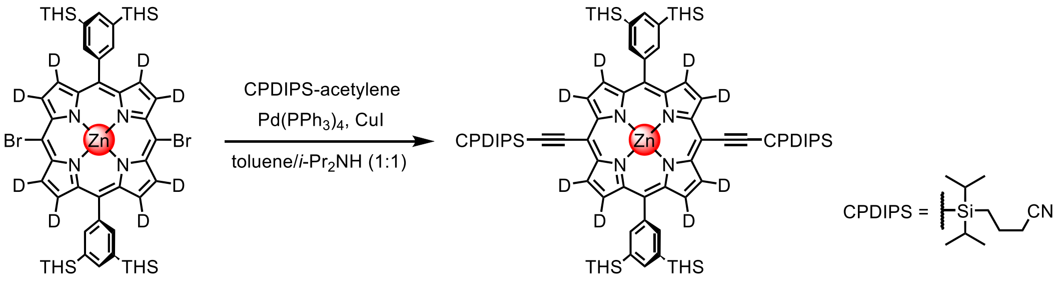

***d*-ZnP1$_{THS}$-Br$_2$** ***d*-ZnP1$_{THS}$-CPDIPS$_2$**

A mixture of ***d*-ZnP1$_{THS}$-Br$_2$** (0.70 g, 0.38 mmol, 1.0 equiv.), $Pd(PPh_3)_4$ (89 mg, 77 μmol, 20 mol%), CuI (15 mg, 77 μmol, 20 mol%) in dry toluene (5.0 mL) and *i*-Pr_2NH (5.0 mL) was degassed by three freeze-pump-thaw cycles. After that, CPDIPS-acetylene (0.22 mL, 0.96 mmol, 2.5 equiv.) was added, and the reaction mixture was stirred at 50 °C under argon for 3 h. After reaction, the resulting mixture was concentrated and purified by flash column chromatography (SiO_2) using pentane/CH_2Cl_2 (7:3) as eluent to give the desired product ***d*-ZnP1$_{THS}$-CPDIPS$_2$** (0.71 g, 89%).

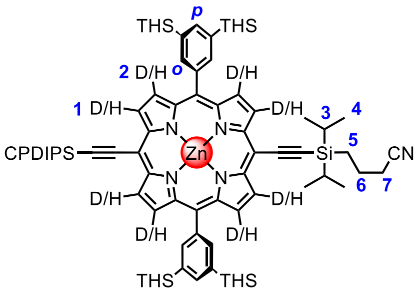

***d*-ZnP1$_{THS}$-CPDIPS$_2$**

^{1}H NMR (600 MHz, $CDCl_3$, 298 K) δ 9.72 (s, 4H, **H$_1$**, 83% D), 8.94 (s, 4H, **H$_2$**, 83% D), 8.25 (d, J = 1.1 Hz, 4H, **H$_o$**), 8.02 (t, J = 1.1 Hz, 2H, **H$_p$**), 2.56 (t, J = 6.8 Hz, 4H, **H$_7$**), 2.34 – 2.12 (m, 4H, **H$_6$**), 1.68 – 0.69 (m, 188H, **H$_{3,4,5,THS}$**).

MALDI-ToF: m/z = 2073.266 ($C_{128}H_{202}D_8N_6Si_6Zn$, $M^{+\bullet}$ requires 2074.505).

UV–vis–NIR ($CDCl_3$, 298 K) λ_{max} / nm (ε / mM^{-1} cm^{-1}): 437 (528), 445 (347), 576 (20.0), 623 (48.3).

4.1.5. Synthesis of ***d*-*lb*-ZnP*N*$_{THS}$-*e*$_1$**

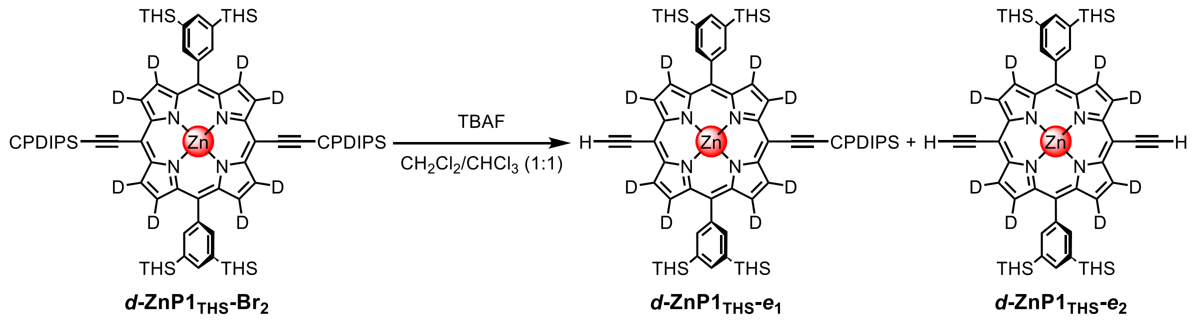

To a solution of ***d*-ZnP1$_{THS}$-Br$_2$** (200 mg, 96 µmol, 1.0 equiv.) in CH_2Cl_2 (15 mL), $CHCl_3$ (10 mL) and pyridine (0.25 mL), tetrabutylammonium fluoride solution (TBAF, 1.0 M in THF, 0.15 mL, 1.0 equiv.) was added. The reaction mixture was stirred at 25 °C for 5 min and the progress was carefully monitored by TLC (SiO_2, eluent: pentane/CH_2Cl_2 = 7:3). CH_3COOH (10 µL) was then added to quench the reaction, and the resulting mixture was passed through a short plug of silica gel using CH_2Cl_2 as eluent. The resulting mixture was further separated by flash column chromatography (SiO_2) using pentane/CH_2Cl_2 (from 9:1 to 4:1) as eluent to give the desired products ***d*-ZnP1$_{THS}$-*e*$_1$** (76 mg, 41%) and ***d*-ZnP1$_{THS}$-*e*$_2$** (62 mg, 38%).

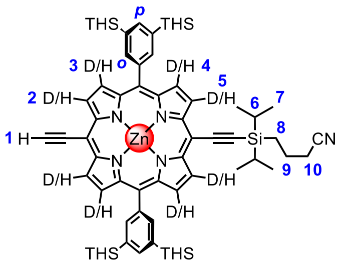

***d*-ZnP1$_{THS}$-*e*$_1$**

^{1}H NMR (600 MHz, $CDCl_3$, 298 K) δ 9.74 (s, 2H, **H$_2$**, 83% D), 9.71 (s, 2H, **H$_5$**, 83% D), 8.95 (s, 2H, **H$_3$**, 83% D), 8.92 (s, 2H, **H$_4$**, 83% D), 8.25 (d, J = 1.1 Hz, 8H, **H$_o$**), 8.00 (t, J = 1.1 Hz, 4H, **H$_p$**), 4.18 (s, 1H, **H$_1$**), 2.55 (t, J = 6.8 Hz, 4H, **H$_{10}$**), 2.32 – 2.08 (m, 4H, **H$_9$**), 1.70 – 0.66 (m, 172H, **H$_{6,7,8,THS}$**).

MALDI-ToF: m/z = 1892.299 ($C_{118}H_{183}D_8N_5Si_5Zn$, $M^{+\bullet}$ requires 1892.376).

UV–vis–NIR ($CDCl_3$, 298 K) λ_{max} / nm (ε / mM^{-1} cm^{-1}): 434 (539), 442 (399), 573 (24.3), 617 (41.4).

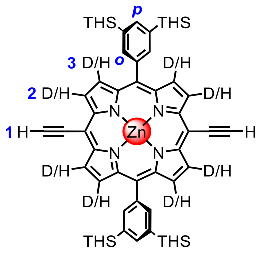

***d*-ZnP1$_{THS}$-*e*$_2$**

^{1}H NMR (600 MHz, $CDCl_3$, 298 K) δ 9.75 (s, 4H, **H$_2$**, 83% D), 8.96 (s, 4H, **H$_3$**, 83% D), 8.27 (d, J = 1.2 Hz, 4H, **H$_o$**), 8.01 (t, J = 1.2 Hz, 2H, **H$_p$**), 4.19 (s, 2H, **H$_1$**), 1.56 – 0.81 (m, 156H, **H$_{THS}$**).

MALDI-ToF: m/z = 1711.101 ($C_{108}H_{164}D_8N_4Si_4Zn$, $M^{+\bullet}$ requires 1711.247).

UV–vis–NIR ($CDCl_3$, 298 K) λ_{max} / nm (ε / mM^{-1} cm^{-1}): 430 (329), 439 (265), 567 (18.5), 611 (21.1).

4.1.6. Synthesis of ***d-lb*-ZnP*N*$_{THS}$-CPDIPS**

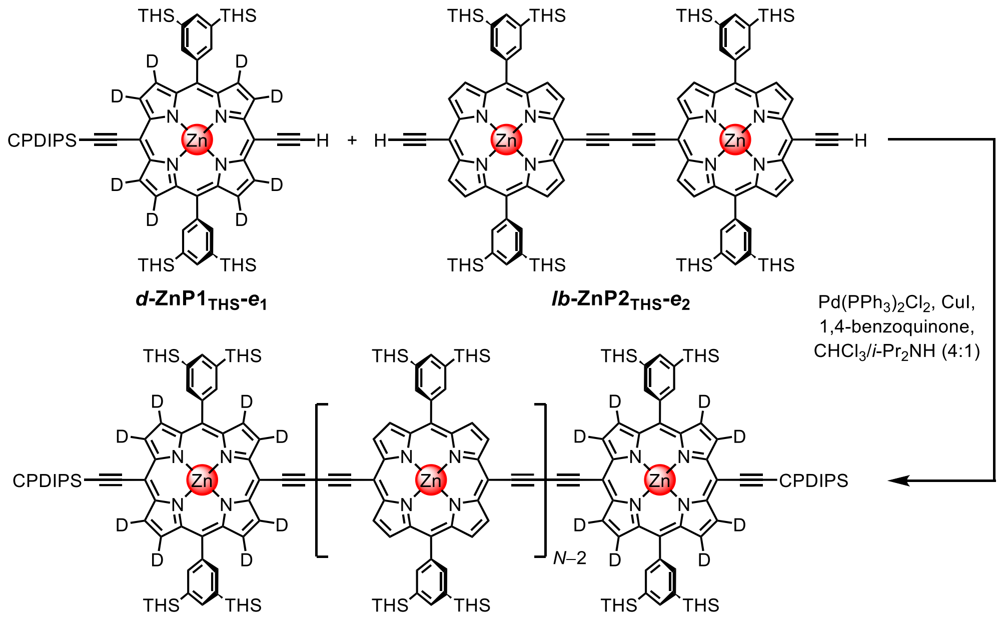

To a solution of ***d*-ZnP1$_{THS}$-*e*$_1$** (56 mg, 29 µmol, 5.0 equiv.) and ***lb*-ZnP2$_{THS}$-*e*$_2$** [2] (20 mg, 5.9 µmol, 1.0 equiv.) in dry $CHCl_3$ (5.0 mL), a catalyst mixture of $Pd(PPh_3)_2Cl_2$ (1.7 mg, 2.5 µmol, 0.4 equiv.), CuI (3.4 mg, 18 µmol, 3.0 equiv.) and 1,4-benzoquinone (7.6 mg, 71 µmol, 16 equiv.) in dry $CHCl_3$ (4.0 mL) and *i*-Pr_2NH (1.0 mL) was added. The reaction mixture was stirred at 25 °C for 1 h. After reaction, the resulting mixture was passed through a short plug of silica gel using CH_2Cl_2 as eluent to give a crude mixture of different oligomers. The crude mixture was further subjected to recycling GPC with toluene/pyridine (99:1) as eluent to separate the desired products ***d-lb*-ZnP4$_{THS}$-CPDIPS$_2$** (21 mg, 50%), ***d-lb*-ZnP6$_{THS}$-CPDIPS$_2$** (7.2 mg, 23%) and ***d-lb*-ZnP8$_{THS}$-CPDIPS$_2$** (2.5 mg, 9%).

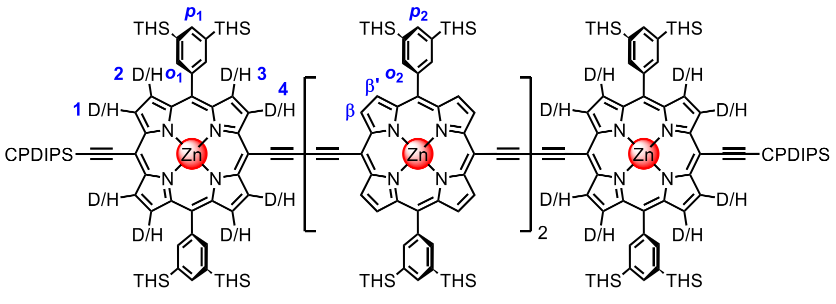

***d-lb*-ZnP4$_{THS}$-CPDIPS$_2$**

^{1}H NMR (600 MHz, $CDCl_3$, 298 K) δ 10.02 – 9.97 (m, 12H, **$H_{4,\beta}$** 83% D for **H_4**), 9.73 (s, 4H, **H_1**, 83% D), 9.09 – 9.05 (m, 8H, **$H_{\beta'}$**), 9.04 (s, 4H, **H_3**, 83% D), 8.97 (s, 4H, **H_2**, 83% D), 8.38 (s, 8H, **H_{o2}**), 8.32 (s, 8H, **H_{o1}**), 8.08 (s, 4H, **H_{p2}**), 8.05 (s, 4H, **H_{p1}**), 2.72 – 0.62 (m, 664H, **$H_{CPDIPS,THS}$**).

MALDI-ToF: m/z = 7189.490 ($C_{452}H_{704}D_{16}N_{18}Si_{18}Zn_4$, $M^{+\bullet}$ requires 7189.098).

UV–vis–NIR ($CDCl_3$, 298 K) λ_{max} / nm (ε / mM^{-1} cm^{-1}): 454 (438), 493 (292), 580 (36.3), 735 (191).

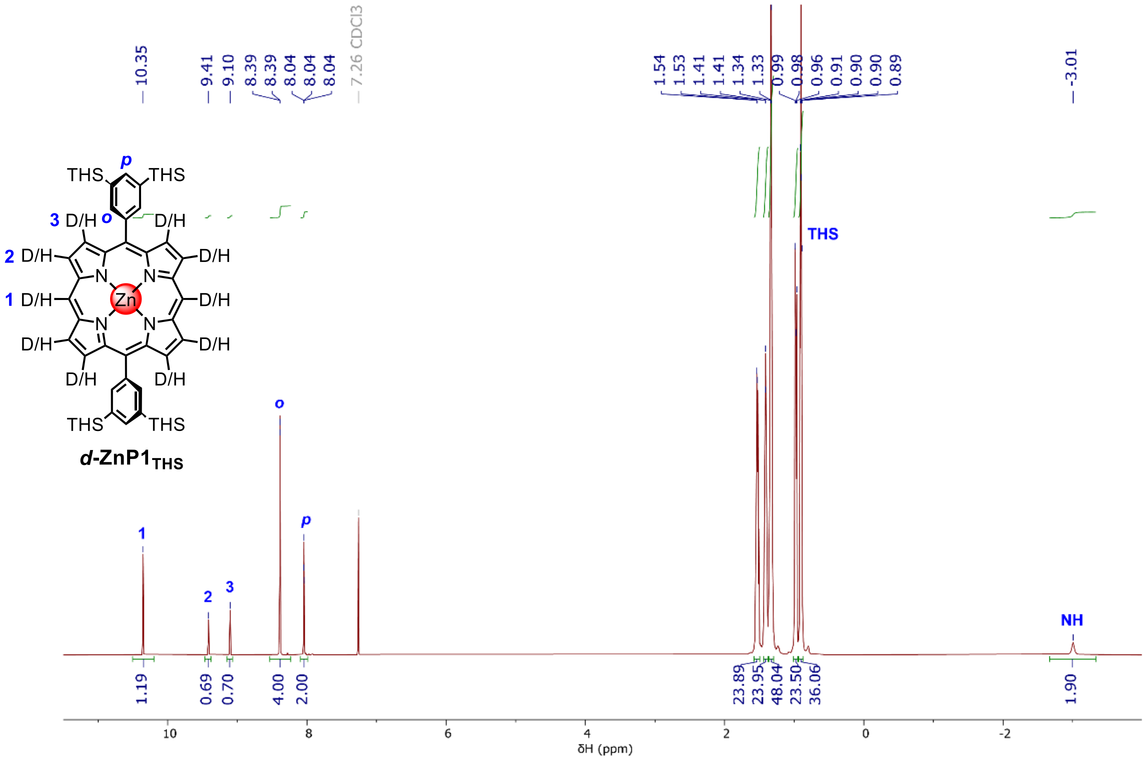

Figure S4-1. ^{1}H NMR spectrum of ***d*-H_2P1$_{THS}$** (600 MHz, $CDCl_3$, 298 K).

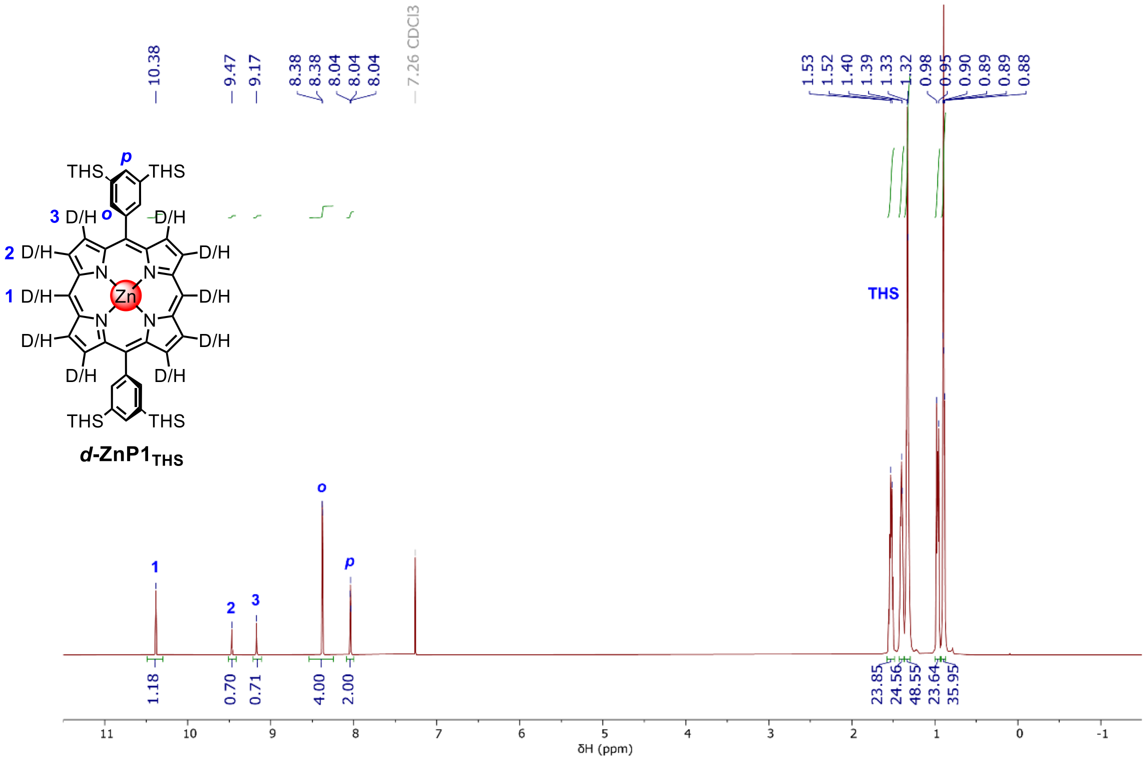

Figure S4-2. ^{1}H NMR spectrum of ***d*-ZnP1$_{THS}$** (600 MHz, $CDCl_3$, 298 K).

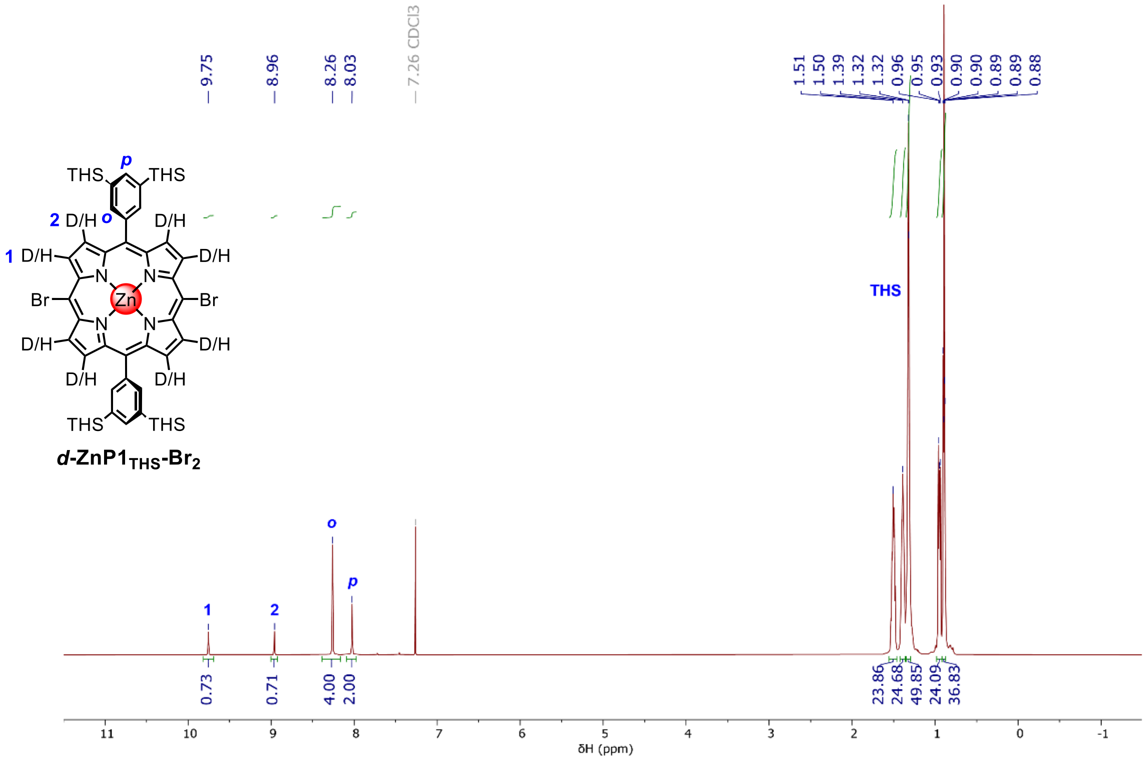

Figure S4-3. ^{1}H NMR spectrum of ***d*-ZnP1$_{THS}$-Br$_2$** (600 MHz, $CDCl_3$, 298 K).

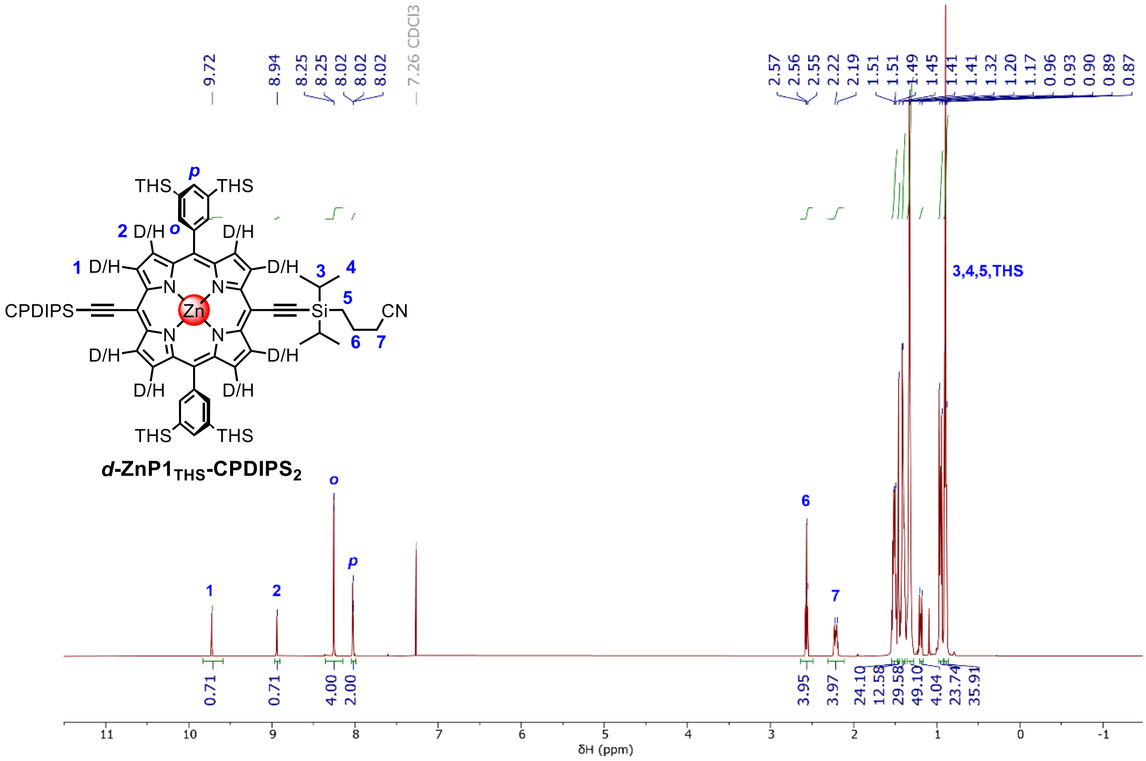

Figure S4-4. ^{1}H NMR spectrum of ***d*-ZnP1$_{THS}$-CPDIPS$_2$** (600 MHz, $CDCl_3$, 298 K).

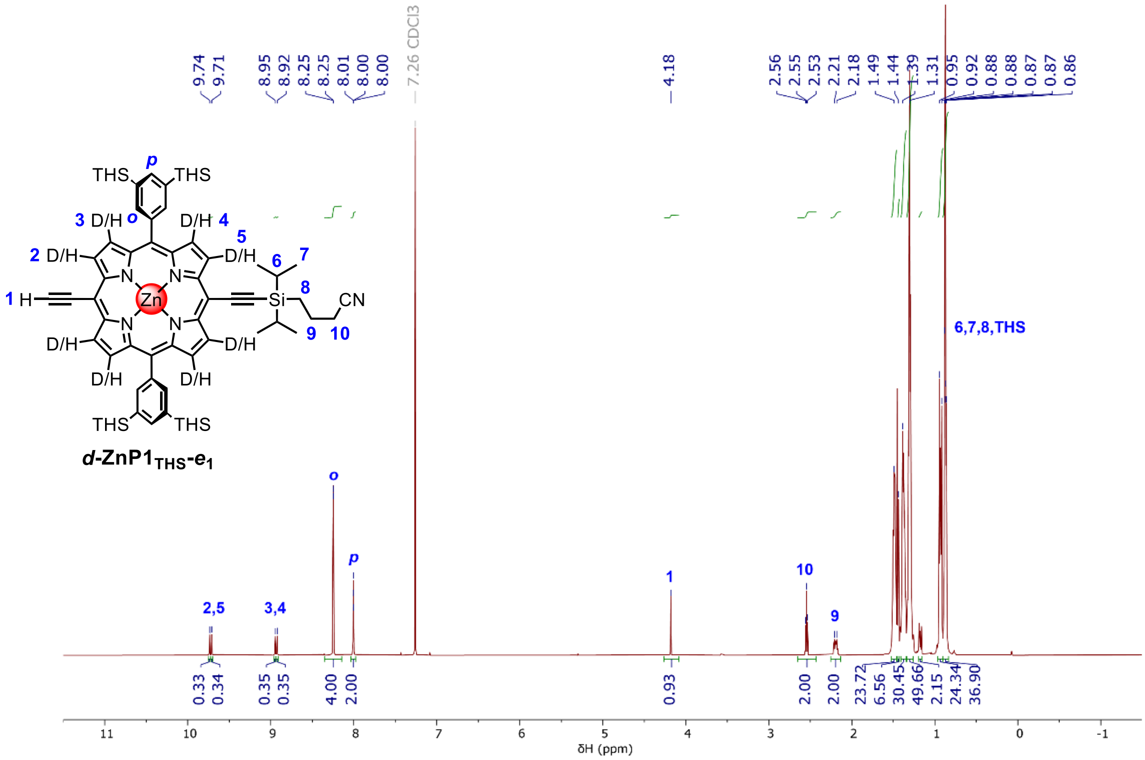

Figure S4-5. ^{1}H NMR spectrum of ***d*-ZnP1$_{THS}$-*e*$_1$** (600 MHz, $CDCl_3$, 298 K).

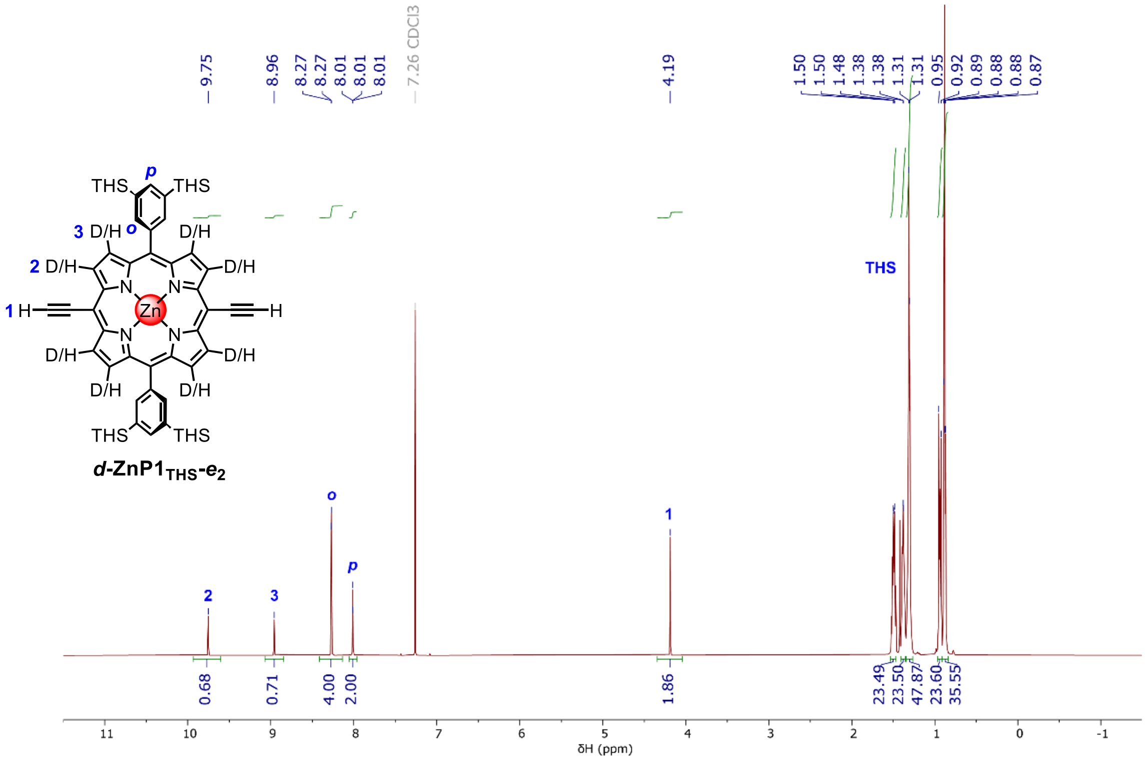

Figure S4-6. ^{1}H NMR spectrum of ***d*-ZnP1$_{THS}$-*e*$_2$** (600 MHz, $CDCl_3$, 298 K).

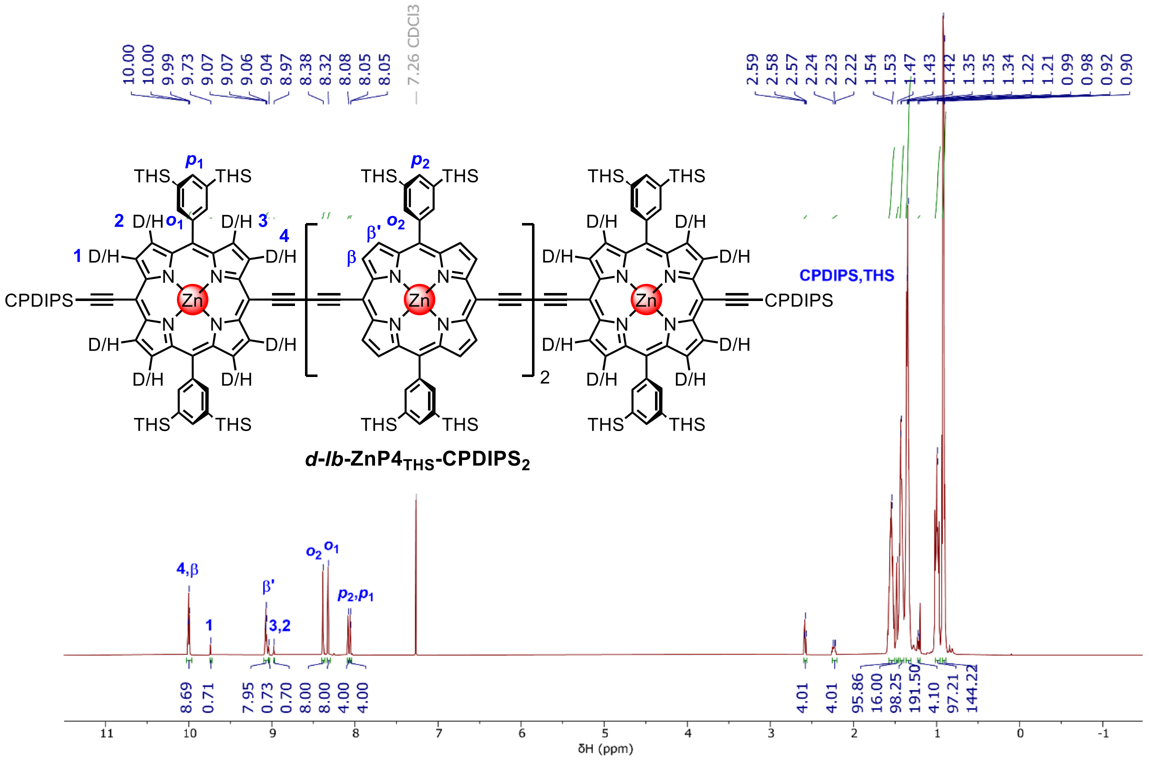

Figure S4-7. ^{1}H NMR spectrum of ***d-lb*-ZnP4$_{THS}$-CPDIPS$_2$** (600 MHz, $CDCl_3$, 298 K).

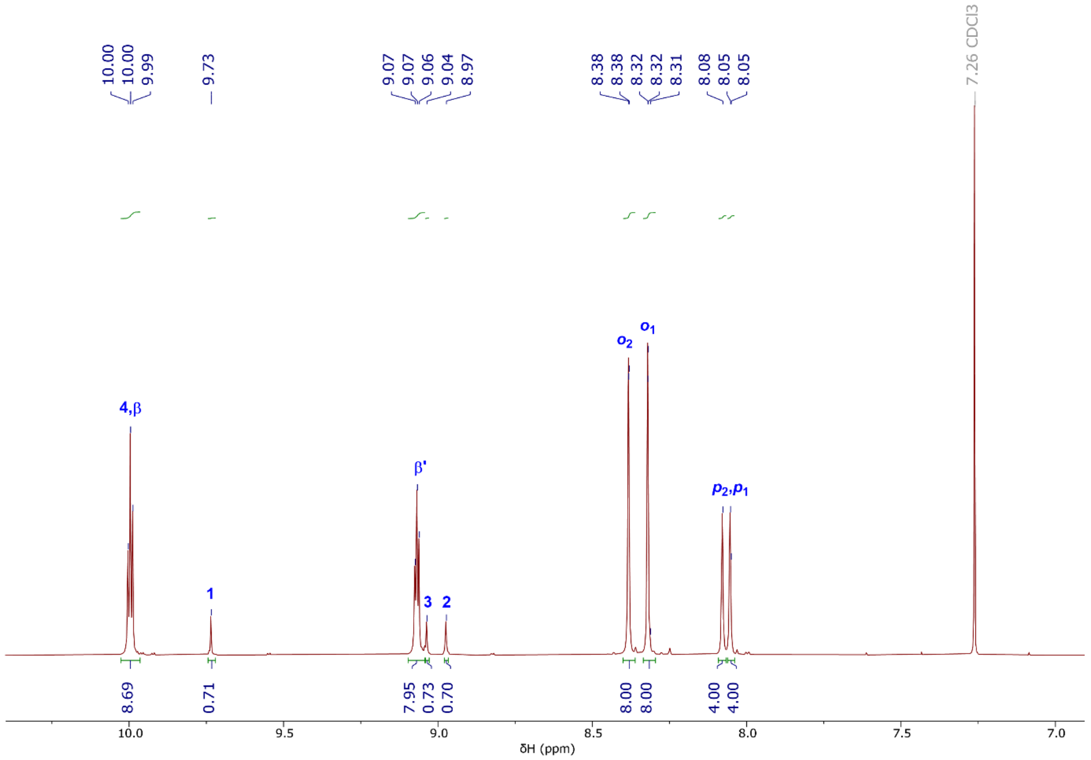

Figure S4-8. Selected region of ^{1}H NMR spectrum of ***d-lb*-ZnP4$_{THS}$-CPDIPS$_2$** (600 MHz, $CDCl_3$, 298 K).

4.2. EPR measurements

A partially deuterated monomer where all beta hydrogens are replaced with deuterium was measured to further investigate the signal shape of the ENDOR spectra and echo detected field sweeps and to explore potential increases in relaxation times. The echo detected field sweeps of the deuterated and non-deuterated monomers are shown in **Figure S4-9** (a). The deuterated compound has a slightly narrower spectral shape and particularly the shoulder arising from $g_{\parallel}$ is less broadened out. While the relaxation times at $g_{\perp}$ are increased by about 15% from deuteration **Figure S4-9** (b), the relaxation times at $g_{\parallel}$ are significantly longer with a more than two-fold increase **Figure S4-9** (c). The less broadened feature arising from $g_{\parallel}$ due to a smaller difference in relaxation times is consistent with the analysis of the spectral shape of the echo detected field sweeps in Section 2.2. In addition, the ^{1}H Mims ENDOR spectra in **Figure S4-9** (d) and (e) show a reduction of the hyperfine couplings and support the assignment of the broad outer features and the shoulders of the central features to the β-hydrogens of the porphyrin anion. Some component at the frequencies arising from those nuclei is still present due to some extent of incomplete deuteration.

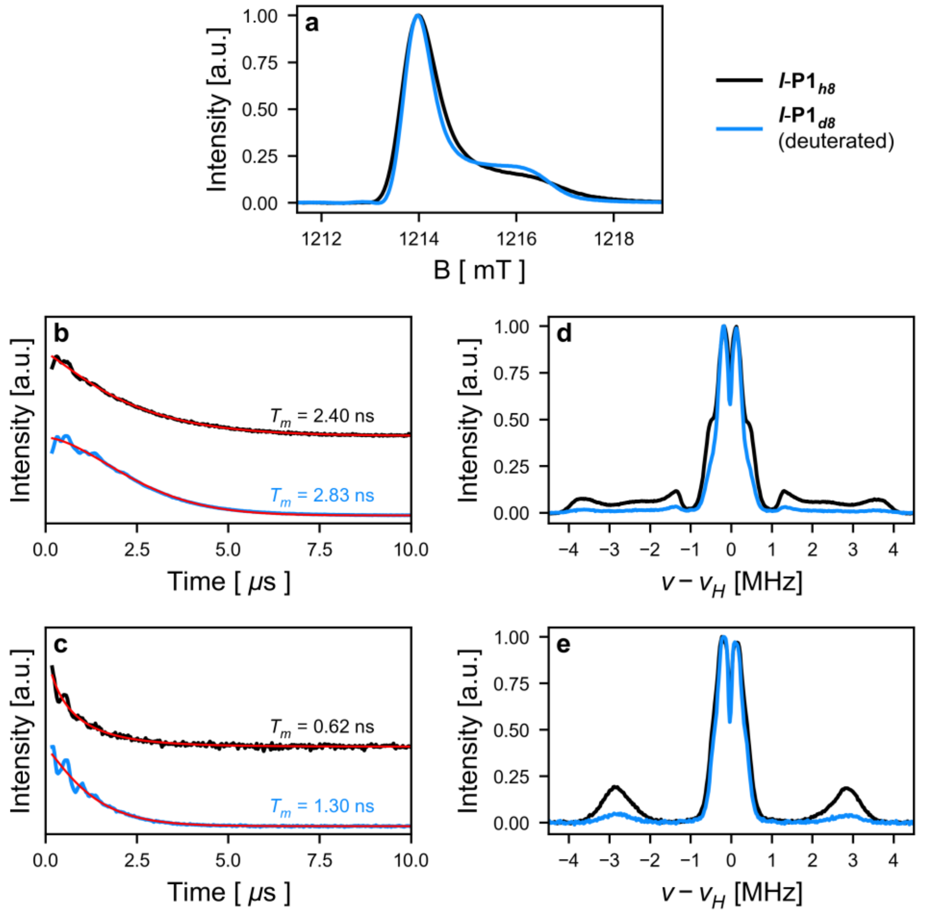

Figure S4-9. (a) Echo detected field sweep spectra recorded at 80 K in deuterated toluene at Q-band frequencies. (b) Echo decay experiment done at field position corresponding to $g_{\perp}$ (at signal maximum) and (c) $g_{\parallel}$ (at shoulder). A stretched exponential decay is fitted (red) to the experimental data and relaxation times shown in the figure. (d) ^{1}H Mims ENDOR spectra recorded at field position corresponding to $g_{\perp}$ (at signal maximum) and (e) $g_{\parallel}$ (at shoulder).

To test the influence of the terminal porphyrins, a partially deuterated ***l*-P4**$^{\bullet-}$ was measured in which the β-H of the terminal porphyrins had been deuterated. **Figure S4-10** shows the ^{1}H ENDOR spectra as well as the residual difference. The difference plot has features even at large hyperfine coupling values, which shows that hydrogens on the terminal porphyrins still significantly contribute to the ENDOR spectrum. If only structures similar to the optimized structure of ***l*-P4**$^{\bullet-}$ would contribute to the spectrum, there would be no signal at large hyperfine couplings as the optimized structure only have very small amounts of spin density on the terminal porphyrins (with hyperfine couplings < 1.3 MHz). These spectra therefore support the analysis that polarons can get localized on different parts of the porphyrin chain due to structural dynamics.

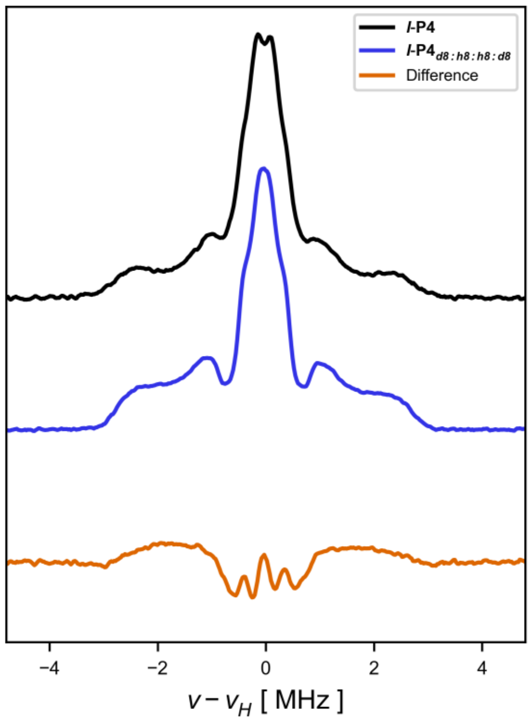

Figure S4-10. ^{1}H ENDOR spectrum of ***l*-P4**$^{\bullet-}$ and a partially deuterated ***l*-P4**$^{\bullet-}$ in which the terminal porphyrins have been deuterated, recorded at field position corresponding to $g_{\perp}$ at 80 K in toluene-d8 at Q-band frequencies.

5. Square wave voltammetry

Square-wave voltammograms were recorded under inert atmosphere inside a glovebox using a PalmSens EmStat3+ Blue and a standard three electrode-setup with a glassy carbon working electrode, platinum wire counter electrode and Ag/AgCl reference electrode. Solutions were prepared with a sample concentration of 0.5 mM in THF using $[Bu_4N][PF_6]$ as a supporting electrolyte at a concentration of 0.1 M. Solvent was filtered over dry alumina and purged of oxygen prior to cycling it into the glove box. The square wave measurements were done with a potential step of 10 mV, a modulation amplitude of 20 mV and a frequency of 2 Hz. The first reduction potentials are estimated from the centre of a Gaussian profile fitted to the square wave voltammograms.

While the exact values of the reduction potentials of the ***l*-PN** are not important in this study, it can be seen from the square wave voltammograms that the reducing agent **CoCp*$_2$** is strong enough to reduce any of the oligomers at least once to the radical anions ***l*-PN$^{\bullet-}$**.

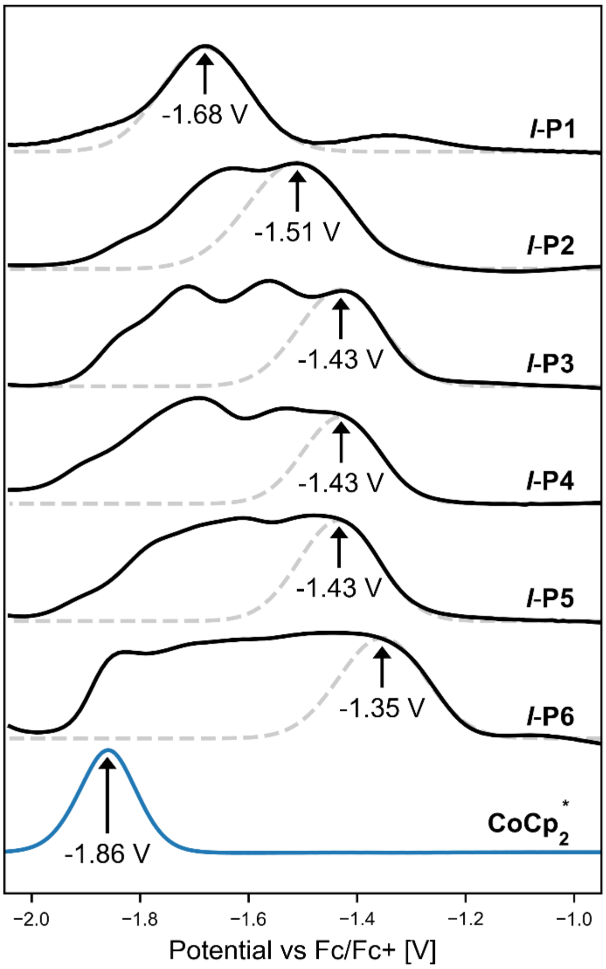

Figure S5-1. Square wave voltammetry of ***l*-PN** (*N*=1-8) and **CoCp*$_2$** recorded in THF with 0.1 M $[Bu_4N][PF_6]$. Arrows indicate estimates of the first reduction potentials.

6. Spectroelectrochemistry

6.1. Methodology

NIR/MIR absorption spectra of reduced porphyrin oligomers were obtained from spectroelectrochemical measurements. Spectra were recorded while simultaneously varying the electrochemical potential using an optically transparent thin layer electrochemistry (OTTLE) cell, which was purchased from Prof Frantisek Hartl (University of Reading, UK). [7] The cell has a path length of 0.2 mm and contains platinum gauze working and counter electrodes and an Ag/AgCl wire reference electrode. Samples were prepared under an inert N_2 atmosphere inside a glove box in 0.1 M solutions of $[Bu_4N][PF_6]$ in THF with oligomer concentrations between 150 to 500 µM. Spectra were recorded on a Bruker Vertex 80 FT-IR spectrometer with a HeNe laser probe at an interval of about 1.2 s while performing a slow cyclic voltammetry with a scan rate of 3.5 mV s^{-1}. NIR measurements were done with a CaF_2 beam splitter, tungsten source, and RT-InGaAS detector; MIR spectra with a KBr beam splitter, Globar source and LN2 cooled-MCT detector. Before both measurements, the optical path was purged with dry N_2 for about 30 minutes. All spectra were background corrected with spectrum of the electrolyte solution.

The raw data were cut to remove noisy regions on the edges of the FT-IR recorded spectra and to only contain the forward sweep of the CV. NIR and MIR regions were combined into one data set and processed together. The first data slice was subtracted from the remaining spectrum as an additional background correction. Since the neutral oxidation states of the porphyrin oligomers investigated here do not have any absorption bands in the NIR and only weak bands in the MIR, this had little effect on the data except subtracting peaks arising from e.g. the solvent or electrolyte due to slightly different concentrations between measurements. Components were extracted using multivariate curve resolution as implemented in pyMCR. Concentration profiles were restricted to sum to one and to have gaussian or semi-sigmoidal shapes. It was possible to fit the data for the ***l*-P*N*** oligomers to N+1 components up to N=6; ***l*-P8** could only be fit to a maximum of eight components. The discussion in the main text is limited to the 1+ oxidation states as the interpretation of the spectra of higher oxidation states is beyond the scope of this paper.

6.2. Spectra

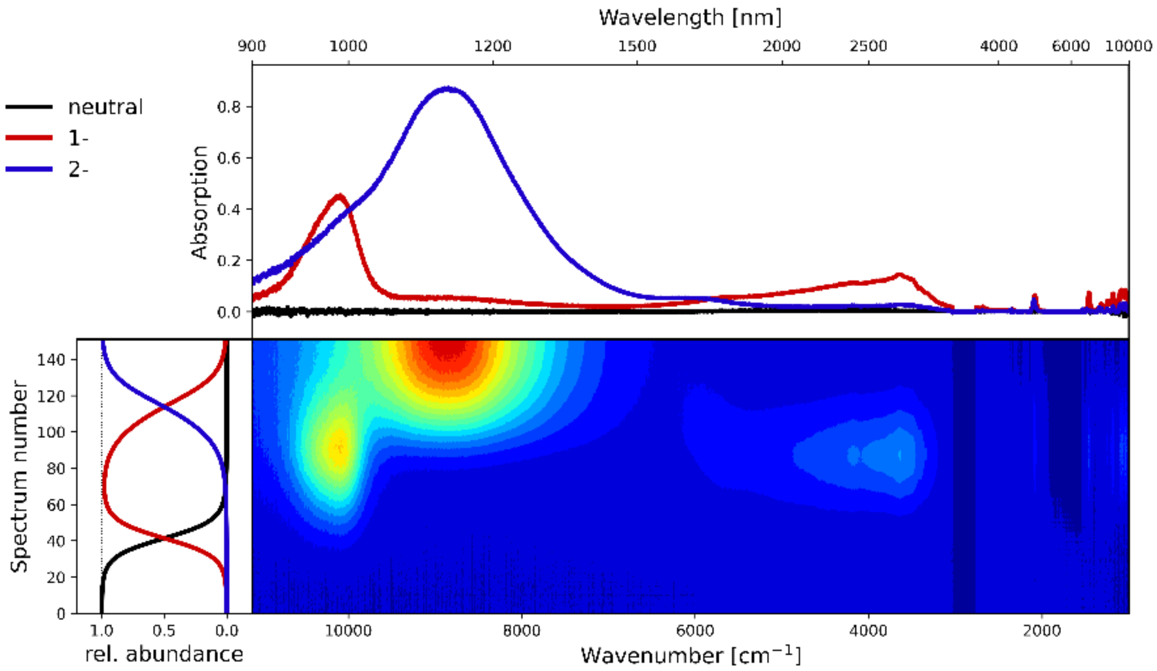

Figure S6-1. Spectroelectrochemical measurements of ***l*-P2**.

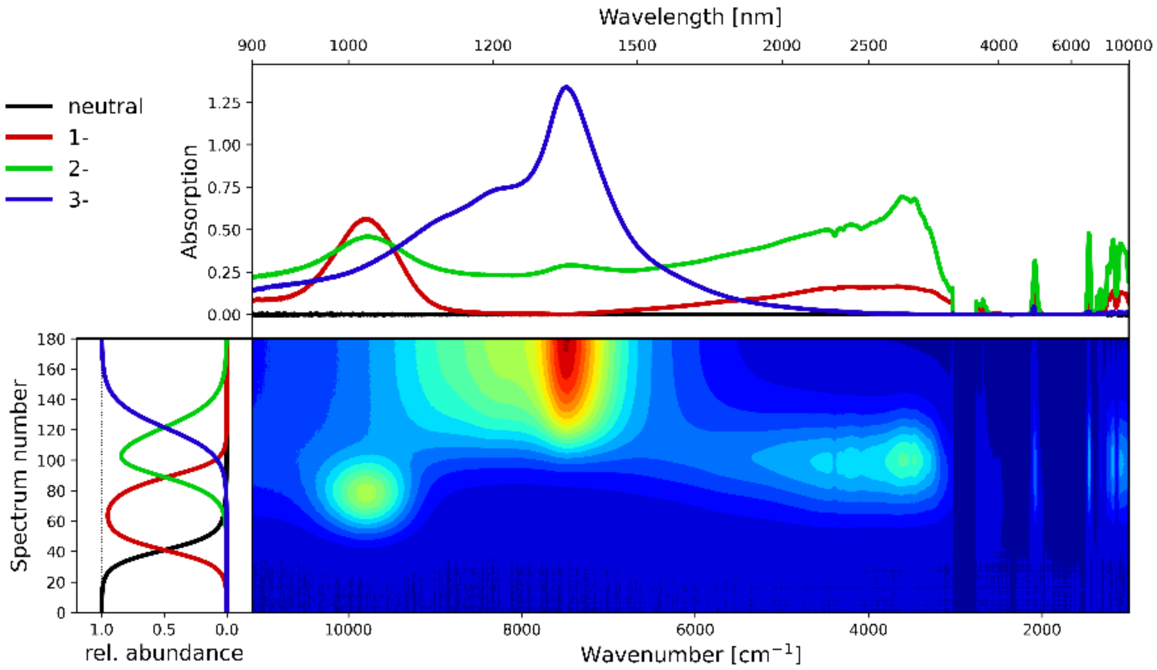

Figure S6-2. Spectroelectrochemical measurements of ***l*-P3**.

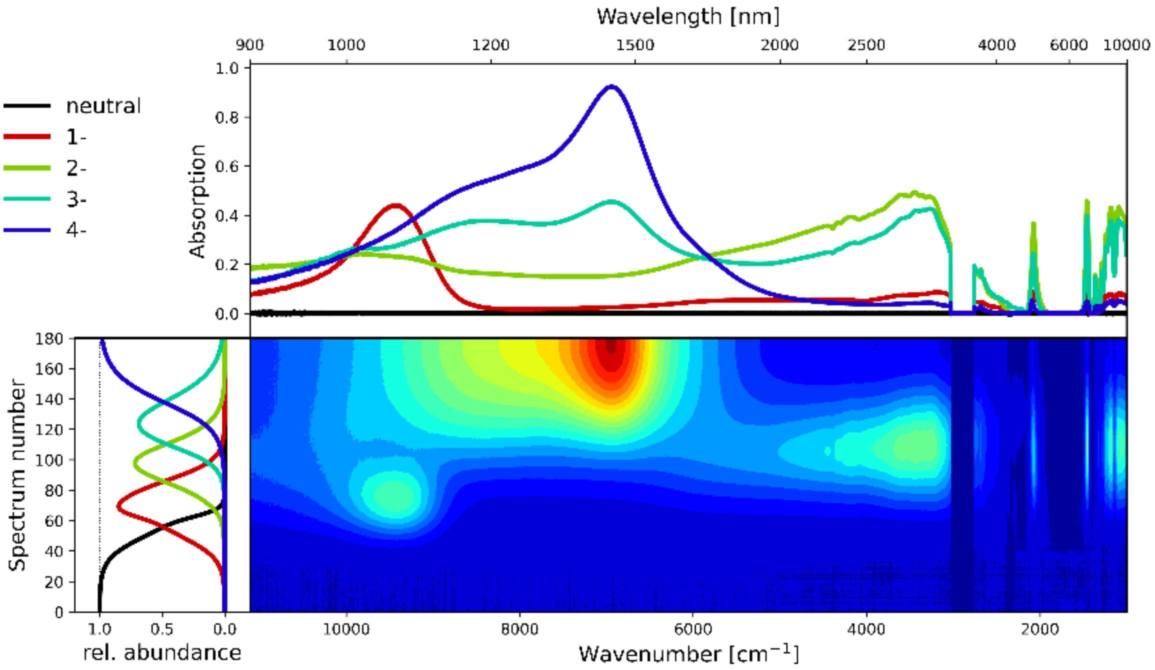

Figure S6-3. Spectroelectrochemical measurements of ***l*-P4**.

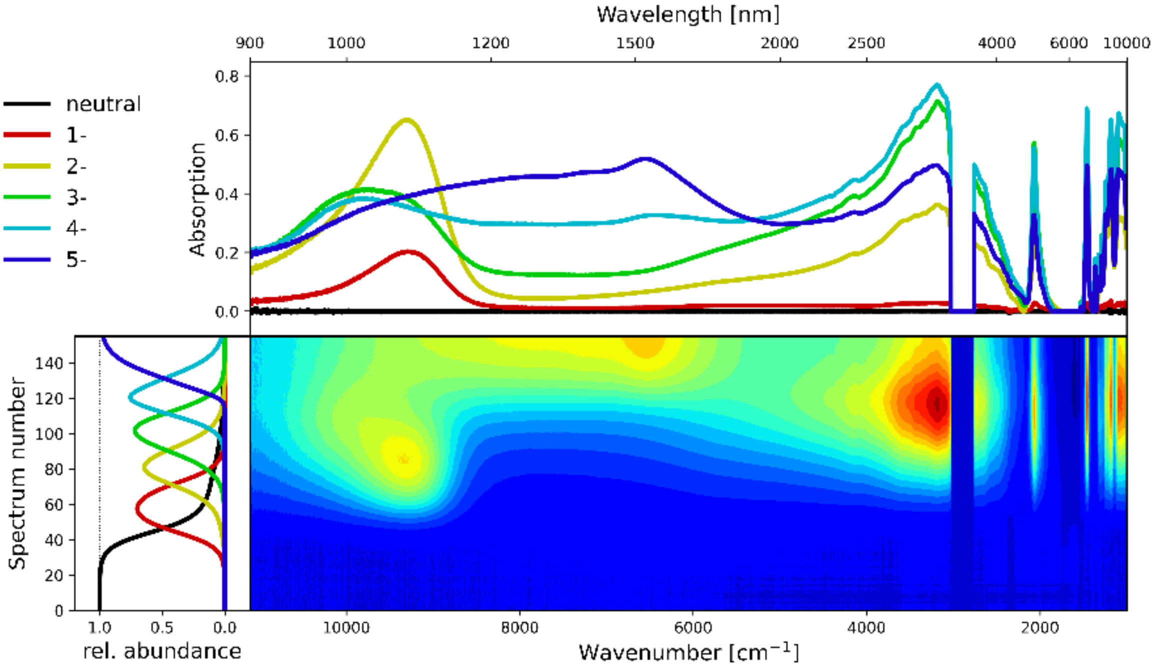

Figure S6-4. Spectroelectrochemical measurements of ***l*-P5**.

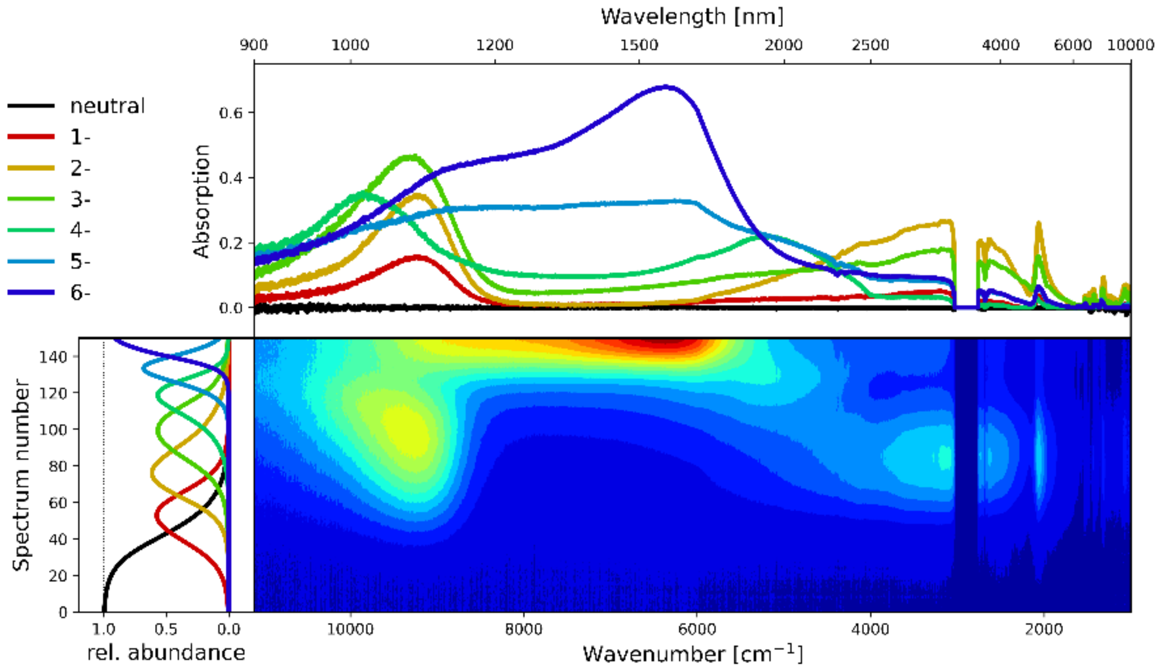

Figure S6-5. Spectroelectrochemical measurements of ***l*-P6**.

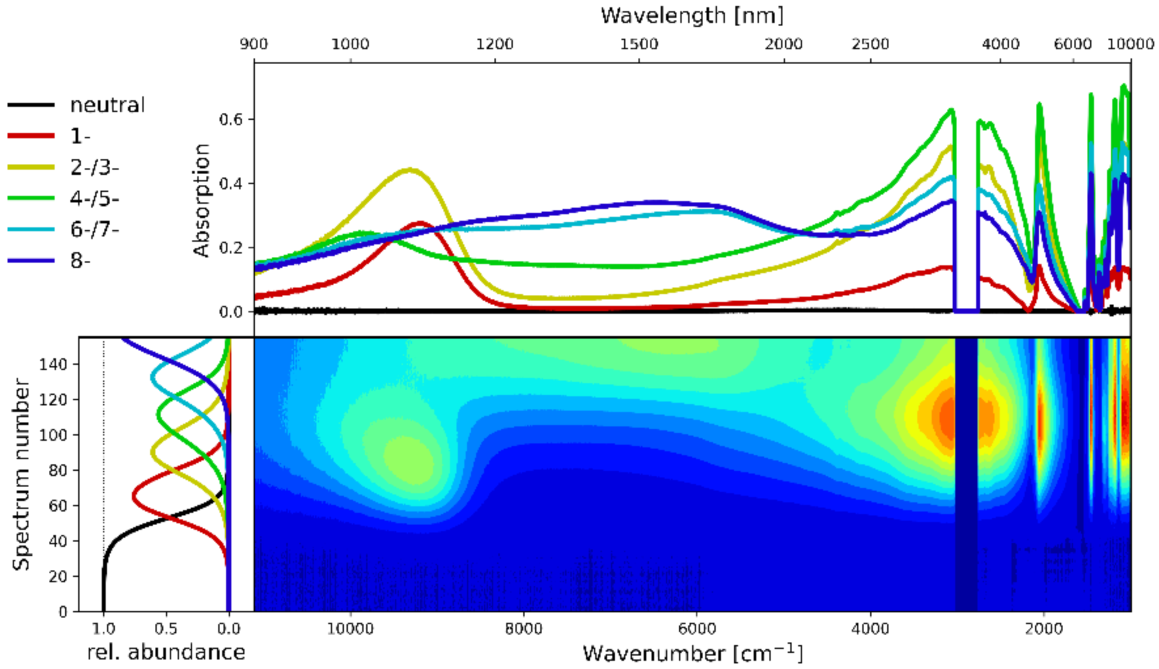

Figure S6-6. Spectroelectrochemical measurements of ***l*-P8**.

Table S6-1. NIR/MIR transition energies of ***l*-P*N*$^{•-}$** (*N* = 2-8)

Structure	P_1 / cm^{-1}	P_2 / cm^{-1}	C≡C / cm^{-1}
***l*-P2$^{•-}$**	3643 ± 44	10086 ± 75	2083 ± 7
***l*-P3$^{•-}$**	3668 ± 285	9803 ± 106	2077 ± 6
***l*-P4$^{•-}$**	3190 ± 75	9432 ± 129	2067 ± 9
***l*-P5$^{•-}$**	3211 ± 139	9293 ± 129	2063 ± 11
***l*-P6$^{•-}$**	3197 ± 70	9253 ± 136	2062 ± 10
***l*-P8$^{•-}$**	-	9211 ± 137	2058 ± 11

6.3. Comparison of transitions in *l*-P*N*$^{•-}$ and *l*-P*N*$^{•+}$

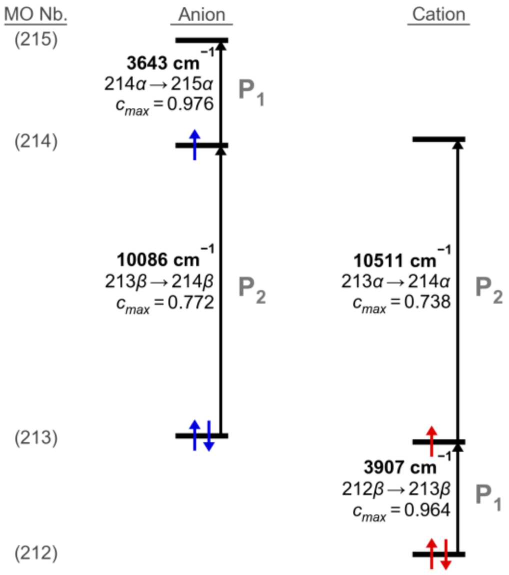

Figure S6-7. Experimental transition energies for the anion and cation of ***l*-P2**. Transitions are assigned based on TD-DFT calculations and list the orbital transitions with the largest coefficient. TD-DFT calculations were done on optimized structures in Gaussian at the lc-ωPBE(ω=0.2)/6-31G* level of theory as described in reference [8].

Table S6-2. TD-DFT excited state information for the lowest two excited states with non-zero oscillator strength in ***l*-P2**$^{\bullet-}$. Orbital transitions with highest coefficient are shown in bold.

**TD-DFT excited state information**		**Assigned to transition**
Orbital transition	Coefficient	
210A ->219A	0.10007	P1
213A ->218A	-0.16134	
**214A ->215A**	**0.97622**	
213B ->214B	0.16078	
214A <-215A	0.14449	
210A ->215A	-0.16534	P2
209B ->218B	-0.13294	
210B ->217B	-0.40364	
211B ->216B	0.29898	
212B ->215B	0.29909	
**213B ->214B**	**0.77231**	
210B <-217B	-0.10818	
211B <-216B	0.11223	
212B <-215B	0.11383	
213B <-214B	0.11738	

Table S6-3. TD-DFT excited state information for the lowest two excited states with non-zero oscillator strength in ***l*-P2**$^{\bullet+}$. Orbital transitions with highest coefficient are shown in bold.

**TD-DFT excited state information**		**Assigned to transition**
Orbital transition	Coefficient	
213A ->214A	0.17183	P1
209B ->214B	-0.19828	
**212B ->213B**	**0.96362**	
212B <-213B	0.14390	
207A ->218A	-0.12125	P2
210A ->215A	-0.40661	
211A ->217A	0.31858	
212A ->216A	0.31884	
**213A ->214A**	**0.73813**	
210B ->216B	0.11105	
211B ->217B	0.11060	
212B ->213B	-0.11826	
212B ->215B	-0.17794	
210A <-215A	-0.11325	
211A <-217A	0.11434	
212A <-216A	0.11579	
213A <-214A	0.11933	

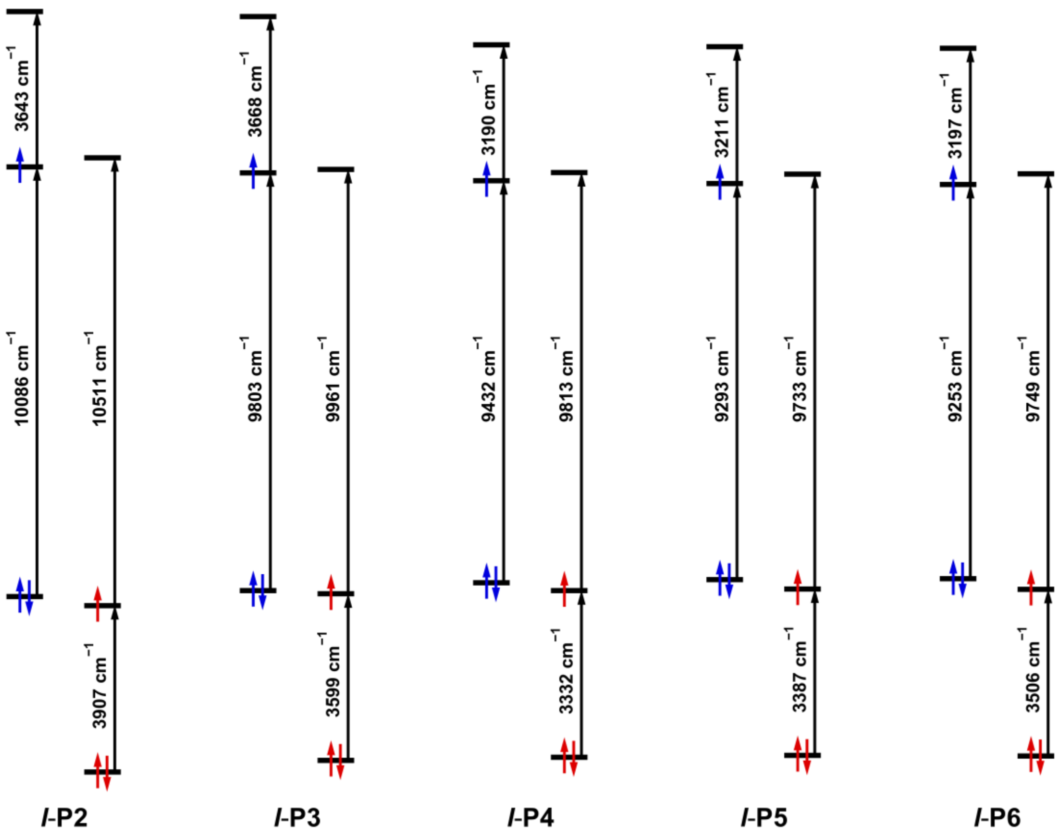

Figure S6-8. Experimental transition energies for the anions and cations of ***l*-PN** (*N* = 2-6). Orbital transitions are assigned in analogy to ***l*-P2**.

7. References

[1] P. N. Taylor, J. Huuskonen, R. T. Aplin, H. L. Anderson, J. Huuskonen, G. Rumbles and E. Williams, "Conjugated porphyrin oligomers from monomer to hexamer." *Chem. Comm.*, **1998**, 8, 909-910.

[2] C. E. Tait, P. Neuhaus, M. D. Peeks, H. L. Anderson and C. R. Timmel, "Transient EPR Reveals Triplet State Delocalization in a Series of Cyclic and Linear π-Conjugated Porphyrin Oligomers", *J. Am. Chem. Soc.*, **2015**, 137, 8284–8293.

[3] J. K. Sprafke, D. V. Kondratuk, M. Wykes, A. L. Thompson, M. Hoffmann, R. Drevinskas, W. H. Chen, C. K. Yong, J. Karnbratt, J. E. Bullock, M. Malfois, M. R. Wasielewski, B. Albinsson, L. M. Herz, D. Zigmantas, D. Beljonne and H. L. Anderson, "Belt-shaped pi-systems: relating geometry to electronic structure in a six-porphyrin nanoring", *J. Am. Chem. Soc.*, **2011**, 133, 17262-17273.

[4] M. Rickhaus, M. Jirasek, L. Tejerina, H. Gotfredsen, M. D. Peeks, R. Haver, H. W. Jiang, T. D. W. Claridge and H. L. Anderson, "Global aromaticity at the nanoscale", *Nat. Chem.*, **2020**, 12, 236-241.

[5] M. J. Frampton, G. Accorsi, N. Armaroli, J. E. Rogers, P. A. Fleitz, K. J. McEwan and H. L. Anderson, "Synthesis and Near-Infrared Luminescence of a Deuterated Conjugated Porphyrin Dimer for Probing the Mechanism of Non-Radiative Deactivation", *Org. Biomol. Chem.*, **2007**, 5, 1056-1061.

[6] F. C. Grozema, C. Houarner-Rassin, P. Prins, L. D. A. Siebbeles and H. L. Anderson, "Supramolecular Control of Charge Transport in Molecular Wires", *J. Am. Chem. Soc.*, **2007**, 129, 13370–13371.

[7] M. Krejčik, M. Deněk and F. Hartl, "Simple construction of an infrared optically transparent thin-layer electrochemical cell: Applications to the redox reactions of ferrocene, Mn2(CO)10 and Mn(CO)3(3,5-di-t-butyl-catecholate)−", *J. Electroanal. chem. interfacial electrochem.*, **1991**, 317, 179-187.

[8] M. D. Peeks, C. E. Tait, P. Neuhaus, G. M. Fischer, M. Hoffmann, R. Haver, A. Cnossen, J. R. Harmer, C. R. Timmel and H. L. Anderson, "Electronic Delocalization in the Radical Cations of Porphyrin Oligomer Molecular Wires", *J. Am. Chem. Soc.*, **2017**, 139, 10461-10471.
